# Do We Adopt the Intentional Stance Toward Humanoid Robots?

**DOI:** 10.3389/fpsyg.2019.00450

**Published:** 2019-03-15

**Authors:** Serena Marchesi, Davide Ghiglino, Francesca Ciardo, Jairo Perez-Osorio, Ebru Baykara, Agnieszka Wykowska

**Affiliations:** ^1^Social Cognition in Human-Robot Interaction Unit, Istituto Italiano di Tecnologia, Genoa, Italy; ^2^School of Computer Science, Faculty of Science and Engineering, Manchester University, Manchester, United Kingdom; ^3^Dipartimento di Informatica, Bioingegneria, Robotica e Ingegneria dei Sistemi, Università di Genova, Genoa, Italy

**Keywords:** social cognition, intentional stance, human–robot interaction, mentalizing, mental states, humanoid robots

## Abstract

In daily social interactions, we need to be able to navigate efficiently through our social environment. According to Dennett (1971), explaining and predicting others’ behavior with reference to mental states (adopting the *intentional stance*) allows efficient social interaction. Today we also routinely interact with artificial agents: from Apple’s *Siri* to GPS navigation systems. In the near future, we might start casually interacting with robots. This paper addresses the question of whether adopting the intentional stance can also occur with respect to artificial agents. We propose a new tool to explore if people adopt the intentional stance toward an artificial agent (humanoid robot). The tool consists in a questionnaire that probes participants’ stance by requiring them to choose the likelihood of an explanation (mentalistic vs. mechanistic) of a behavior of a robot iCub depicted in a naturalistic scenario (a sequence of photographs). The results of the first study conducted with this questionnaire showed that although the explanations were somewhat biased toward the mechanistic stance, a substantial number of mentalistic explanations were also given. This suggests that it is possible to induce adoption of the intentional stance toward artificial agents, at least in some contexts.

## Introduction

Over the last decades, new technologies have entered our houses inexorably, becoming an integral part of our everyday life. Our constant exposure to digital devices, some of which seemingly “smart,” makes the interaction with technology increasingly more smooth and dynamic, from generation to generation (Baack et al., 1991; Gonzàles et al., 2012; Zickuhr and Smith, 2012). Some studies support the hypothesis that this exposure is only at its beginning: it seems likely that technologically sophisticated artifacts, such as humanoid robots, will soon be present in our private lives, as assistive technologies and housework helpers (for a review see Stone et al., 2016).

Despite the fact that we are becoming increasingly habituated to technology, little is known about the social cognitive processes that we put in place during the interaction with machines and, specifically, with humanoid robots. Several authors have theorized that humans possess a natural tendency to anthropomorphize what they do not fully understand. Epley et al. (2007) for instance, defined anthropomorphism as the attribution of human-like characteristics and properties to non-human agents and/or objects, independently of whether they are imaginary or real. The likelihood of spontaneous attribution of anthropomorphic characteristics depends on three main conditions (Epley et al., 2007; Waytz et al., 2010): first, the availability of characteristics that activate existing knowledge that we have about humans; second, the need of social connection and efficient interaction in the environment; and, finally, individual traits (such as the need of control) or circumstances (e.g., loneliness, lack of bonds with other humans). In the Vol. I of the Dictionary of the History of Ideas, Agassi (1973) argues that anthropomorphism is a form of parochialism allowing projecting our limited knowledge into a world that we do not fully understand. Some other authors claim that we, as humans, are the foremost experts in what it means to be human, but we have no phenomenological knowledge about what it means to be non human (Nagel, 1974; Gould, 1996). For this reason, when we interact with entities for which we lack specific knowledge, we commonly choose “human” models to predict their behaviors.

A concept similar, but not identical, to anthropomorphism is the *intentional stance*. Intentional stance is a narrower concept than anthropomorphism, as the latter seems to be involving attribution of various human traits, while adopting the intentional stance refers more narrowly to adopting a strategy in predicting and explaining others’ behavior with reference to mental states. The concept of intentional stance has been introduced by Daniel Dennett who proposed that humans use different strategies to explain and predict other entities’ (objects, artifacts, or conspecifics) behaviors (Dennett, 1971, 1987). Dennett defines three main strategies or “stances” that humans use. Consider chemists or physicists in their laboratory, studying a certain kind of molecules. They try to explain (or predict) the molecules’ behavior through the laws of physics. This is what Dennett calls the *physical instance*. There are cases in which laws of physics are an inadequate (or not the most efficient) way to predict the behavior of a system. For example, when we drive a car, we can fairly predict that the speed will decrease if we push the brake pedal, since the car itself is designed this way. To make this kind of prediction, we do not need to know the precise physical mechanisms behind all atoms and molecules in the braking system of the car, but it is sufficient to rely on our experience and knowledge of how the car is designed. Dennett describes this as the *design stance*. Dennett proposes the existence of also a third strategy, the *intentional stance*. *Intentional stance* relies on the ascription of beliefs, desires, intentions and, more broadly, mental states to a system, in order to explain and predict its behavior: “(…) there is yet another stance or strategy that one can adopt: the intentional stance. Here is how it works: first you decide to treat the object whose behavior is to be predicted as a rational agent; then you figure out what beliefs that agent ought to have, given its place in the world and its purpose. Then you figure out what desires it ought to have, on the same considerations, and finally you predict that this rational agent will act to further its goals in the light of its beliefs. A little practical reasoning from the chosen set of beliefs and desires will in many – but not in all – instances yield a decision about what the agent ought to do; that is what you predict the agent will do” (Dennett, 1987, p. 17).

Please note that the concept of intentional stance, following Dennett’s description, can be distinguished from the concept of Theory of Mind (ToM) in the sense in which it has been used in developmental psychology (e.g., Baron-Cohen, 1997). Although the two concepts are very tightly linked, and often subsumed under a common conceptual category (e.g., Baron-Cohen, 1997), they do differ with respect to the context in which they have been introduced in literature, and, what follows, in the empirical ways of addressing the concepts. As described above, the intentional stance has been introduced by Dennett in the context of two other stances (or strategies) that allow predicting and explaining behavior of an observed system. Therefore, if an empirical test is set out to examine whether one adopts the intentional stance toward a system, the contrasting conditions should be either the design stance or the physical stance. On the contrary, the ToM has been introduced (Leslie, 1994; Baron-Cohen, 1997) to denote a capacity of understanding mental states of other humans that explain and predict their behavior, but that might be different from one’s own mental states (perspective taking) and might misrepresent reality (false beliefs). In this context, empirical tests that address the concept of ToM will not contrast the ToM condition with design or physical stance, but rather will make a contrast between different mental states (e.g., true vs. false beliefs). This implies that if one has a ToM of another human’s behavior, one has adopted the intentional stance, but not necessarily vice versa. In the “Sally and Anne” test (Wimmer and Perner, 1983; Baron-Cohen et al., 1985), I can adopt intentional stance toward Sally (explain her behavior with reference to mental states) but attribute incorrect mental states to her (that is, not understand that her perspective is different from mine, or from reality). So I can fail in ToM test, but I can still adopt the intentional stance. In this way, with reference to human agents, the intentional stance is a necessary condition for ToM, but not a sufficient one.

Even though we distinguish intentional stance from ToM, the idea of adopting the intentional stance toward others shares with the ToM the reference to mental states during social cognition processes. Therefore, it might fall under the same criticism as the ToM accounts of social cognition. In literature, there has been a heated debate regarding ToM accounts of social cognition (e.g., Gopnik and Wellman, 1992). Some authors (e.g., Gallagher, 2001; Zahavi and Gallagher, 2008) proposing the “direct perception” account deny the core assumption of ToM accounts, which is based on the implication: from the observed behavior of others, we infer the unobservable mental states to explain and predict the behavior. The authors propose that there is no distinction between observable behavior and unobservable mental states, as the observed behavior contains already cognitive/mental processes, and is perceived as such. Another line of criticism is embedded in the enactivist or interactionist accounts of social cognition (Zahavi and Gallagher, 2008; De Jaegher et al., 2010; Michael, 2011). The criticism of ToM based on those approaches is that ToM account is grounded too much in spectatorial, individualist, and cognitivist assumptions, which rely on the observer passively viewing behaviors of others and making inferences about mental states. The interactionists propose that social cognition is rather a participatory and interactive process allowing humans to understand behavior of others without mindreading, through “making sense of the situation together” (Bohl and van den Bos, 2012, p. 3), based on the interaction processes themselves, at the supra-individual level (e.g., Reddy and Morris, 2004, see also Michael, 2011; Bohl and van den Bos, 2012 for a review). Some authors (e.g., Bohl and van den Bos, 2012) postulate that neither of the accounts (neither ToM nor the interactionist/enactivist accounts) is sufficient to explain all domains of social cognition. This is because they address different type of processes: while interactionists accounts are better suited to explain Type I (fast, efficient, stimulus-driven and inflexible) processes of social cognition, ToM accounts for Type II (slow, cognitively laborious, flexible, and often conscious) processes (Bohl and van den Bos, 2012, p. 1).

In either case, the aim of this paper is ***not*** to defend ToM accounts of social cognition. In our view, addressing the question of whether humans adopt the intentional stance toward artificial agents is of interest independent of the debate regarding various accounts of social cognition. This is because, even if ToM accounts cannot explain various aspects of social cognition, humans do sometimes use mentalistic vocabulary when describing behavior of others. Except for radical interactionists, it is rather agreed upon that ToM accounts for at least a set of processes occurring during social interaction. Bohl and van den Bos (2012), for example, point out that interactionism cannot explain situations when interactions are not going smooth, when we try to explain others’ behavior with reference to mental states because of competition, disagreement, or conflict. Therefore, as long as one does not postulate that ToM is the foundation for *all* social cognitive processes, it remains justified to propose ToM as one aspect of social cognition. In this context, it is theoretically interesting to ask whether humans could potentially also use mentalistic vocabulary toward artificial agents, and if so, under what conditions. Perhaps this is not the only factor that would have an impact on social engagement or interaction with those agents, but certainly one that does play a role, along with other factors. Furthermore, it is an interesting question from the point of view of artificial intelligence, as adopting the intentional stance toward artificial agents is some form of the Turing test (Turing, 1950). Therefore, given the above considerations, we set out to explore whether humans can adopt the Intentional Stance toward artificial agents, by contrasting mentalistic interpretations of behavior with mechanistic ones.

In this context, it needs to be noted, however, that adopting the *intentional stance* toward an artifact (such as a humanoid robot) does not necessarily require that the artifact itself possesses true intentionality. Adopting the *intentional stance* might be a useful or default way to explain a robot’s behavior. Perhaps we do not attribute mental states to robots but we treat them ***as if*** they had mental states (Thellman et al., 2017). Breazeal and Scassellati (1999) highlighted that this process does not require endowing machines with mental states in the human sense, but the user might be able to intuitively and reliably explain and predict the robot’s behavior in these terms. *Intentional stance* is a powerful tool to interpret other agents’ behavior. It leads to interpreting behavioral patterns in a general and flexible way. Specifically, flexibility in changing predictions about others’ intentions is a pivotal characteristic of humans. Adopting the intentional stance is effortless for humans, but of course, it is not the perfect strategy: if we realize that this is not the best stance to make predictions, we can refer to the design stance or even the physical stance. The choice of which stance to adopt is totally free and might be context-dependent: it is a matter of which explanation works best. Let us consider our interactions with smartphones. The way we approach them is fluid: we adopt the design stance when we hear a “beep” that notifies us about a text message or an e-mail, but we might switch to the intentional stance when voice recognition such as Apple’s Siri is not responding to us adequately, and gives us an incorrect answer to our question. In fact, it might even happen that we become frustrated and ask our smartphone a rhetorical question “why are you so stupid?”. In this context, it is important to consider also cultural differences: the likelihood of adopting the intentional stance toward artifacts might differ from culture to culture (Dennett, 1987).

Taken together, it is intriguing whether humans do sometimes adopt the intentional stance toward humanoid robots, at least in some contexts.

## Aims of Study

In order to address the question of whether humans adopt the intentional stance toward a robot, we created a tool (the Intentional Stance Questionnaire, ISQ) that should probe the adoption of intentional stance toward a specific robot platform, the iCub robot (Metta et al., 2010; Natale et al., 2017).

The aims of this study were the following:

### Aim 1

Developing a *tool* that would allow for measuring whether humans would sometimes (even if only in some contexts) adopt the intentional stance toward a robot. The study reported in this paper aimed at providing a baseline “intentional stance” score (ISS). As such, it could subsequently serve as a score against which other experimental conditions may be compared (in future research). When other conditions in which participants’ likelihood of adopting the intentional stance are manipulated experimentally (for example, through robot’s appearance, behavior, a specific mode of interaction, etc.) a given ISS measured with ISQ, might then be compared to the baseline ISS reported here. This should allow for evaluating whether the experimental factors up- or down-modulate baseline ISS. Please note that the study reported here focused only on the baseline score.

### Aim 2

Exploring if humans *would sometimes* adopt the intentional stance toward robots. We were interested in whether some contexts can evoke mentalistic explanations of behavior of a humanoid robot. Please note that we focused on only one type of mental states (and, what follows, intentionality) attributed to artificial agents, namely the more explicit, propositional-attitudes mental states carrying the inherent “*aboutness*” (e.g., belief that…, desire that…, see Brentano, 1874; Churchland, 1981). Our study did not address more implicit forms of adopting the intentional stance.

As argued above, our aim was not to defend the ToM accounts of social cognition. Furthermore, we also did not aim at determining how appearance of a robot influences adoption of the intentional stance, or whether the degree of adoption of the intentional stance is smaller or larger as compared to other agents (humans or non-anthropomorphic robots). On the contrary, we intended a more modest aim: our goal was only to establish a baseline ISS toward a specific humanoid robot iCub.

We created 34 fictional scenarios, in which iCub appeared (in a series of photographs) engaged in different activities in a daily life context. Each scenario consisted of three aligned pictures, depicting a sequence of events. For each scenario, participants had to rate (by moving a slider on a scale) if iCub’s behavior is motivated by a mechanical cause (referring to the design stance, such as malfunctioning, calibration, etc.) or by a mentalistic reason (referring to the intentional stance, such as desire, curiosity, etc.).

## Materials and Methods

### Sample

One hundred and six Italian native speakers with different social and educational backgrounds (see Table 1 for demographical details) completed our InStance questionnaire. Data collection was conducted in accordance with the ethical standards laid down in the Code of Ethics of the World Medical Association (Declaration of Helsinki), procedures were approved by the regional ethics committee (Comitato Etico Regione Liguria).

**Table 1:** Demographic details of the sample (N = 106).

Demographic characteristic	
Age (years), mean (SD) [min, max]	33.28 (12.92) [18,72]
Female, n (%)	68 (64.2)
Education (years), mean (SD) [min, max]	16.43 (3.04) [8, 24]


### Questionnaires

#### InStance Questionnaire (ISQ)

**FIGURE 1:**
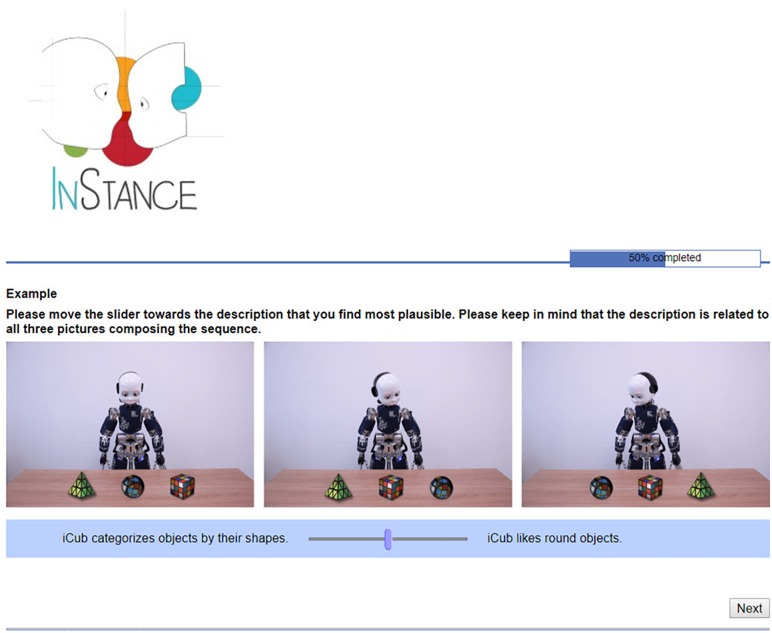
Screenshot from the InStance Questionnaire in English.

Each item of ISQ was composed of a scenario and two sentences with a bipolar scale and a slider between the two sentences (one of the sentences was positioned on the left, and the other one on the right extreme of the scale), see Figure 1 for an example. For a complete list of images and sentences included in the questionnaire see the Supplementary Material (English version), or the following link (for English and Italian version): https://instanceproject.eu/publications/rep.

We created 34 scenarios depicting the iCub robot interacting with objects and/or humans. Each scenario was composed of three pictures (size 800 × 173.2 pixels). Out of the 34 scenarios, 13 involved one (or more) human interacting with the robot; 1 scenario showed a human arm pointing to an object; 20 scenarios depicted only the iCub robot. Ten scenarios included pictures digitally edited (Adobe Photoshop CC 2018). The types of action performed by iCub depicted in the scenarios were: grasping, pointing, gazing, and head movements.

Each item included two sentences, in addition to the scenario. One of the sentences was always explaining iCub’s behavior referring to the design stance (i.e., mechanistic explanation), whereas the other was always describing iCub’s behavior referring to mental states (i.e., mentalistic explanation). Mentalistic and mechanistic sentences were equally likely to appear either on the left or on the right side of the scale, as the mapping between type of sentence and position was counterbalanced across items. Moreover, we kept iCub’s emotional expression constant across the scenarios to avoid bias toward mentalistic explanations.

In order to be certain that the sentences were describing the action in a mechanistic or mentalistic way, prior to data acquisition, we distributed the sentences (only sentences alone, with no associated pictures) to 14 volunteers who had a degree in philosophy (all Italian native speakers) to rate on a 10-point Likert scale how much they understood each sentence as mechanistic or mentalistic (0 = totally mechanistic, 10 = totally mentalistic). As no scenario was presented with the sentences, the raters were not informed that the sentences were created to describe the behavior of a humanoid robot. In addition, the subject of each sentence was named as a general “Agent A” to avoid any bias arising from the name of the robot. The mean score given was 8.2 for the mentalistic sentences and 4.3 for the mechanistic sentences. Based on the responses in this survey, we modified the sentences that were not matching our intent. In particular, we modified sentences that obtained an average score between 4 and 5 (meaning that they were not clearly evaluated as mechanistic or mentalistic), using as cut-off 4.3 since most of the critical sentences were from the mechanistic group (14 out of 15 sentences). We modified 15 out of the 35 initial pairs of sentences to match our intended description (mentalistic or mechanistic).

#### Other Questionnaires

In addition to the InStance questionnaire, we administered the Italian version of the Reading the Mind in the Eyes (Baron-Cohen et al., 1999, 2001; Serafin and Surian, 2004) and the ToM subscale of the questionnaire developed by Völlm et al. (2006). These tests were used as a control to check for outliers in ToM abilities.

##### Reading the Mind in the Eyes

The Reading the Mind in the Eyes test was developed by Baron-Cohen et al. (1999, 2001) to test the abilities of infering other’s mental states through only looking at others’ eyes. In this questionnaire, participants are asked to view 36 photos of people’s eyes. Below the photographs of the eyes, four adjectives are presented. Participants are asked to choose one adjective that describes best the photograph they are looking at. We selected this test, as it is one of the most used tests to measure ToM abilities.

##### A test of Völlm et al. (2006)

Völlm et al. (2006) developed a questionnaire to evaluate the neural correlates of ToM and empathy in an fMRI study. In this test, the presented stimuli are non-verbal cartoon stripes of three frames: participants are instructed to look at the stripes and choose the frame that, according to them, would best conclude the depicted story. For the purpose of this study, we presented only the 10 items included in the ToM subscale of the test. We selected this test as a non-verbal test of mentalising abilities, complementary to the *Reading the Mind in the Eyes* test.

##### Data acquisition for the InStance questionnaire

All the questionnaires were administered via SoSci survey^1^. Participants received the URL addresses of all the questionnaires. Participants were asked to fill out the questionnaires in the order they were provided: a generic information questionnaire, ISQ, the Mind in the Eyes and finally the Völlm et al. (2006) questionnaire. The generic information questionnaire collected demographic information of participants (see Table 1) and whether they were familiar with robots or not (see Supplementary Material). The ISQ was composed of 34 items and 1 example item. Only one item at a time was presented (cf. Figure 1).

In each item, participants were explicitly instructed to move a slider on a bipolar scale toward the sentence that, in their opinion, was a more plausible description of the story depicted in the scenario. As illustrated in Figure 1, the two statements (mentalistic and mechanistic) were placed at the two bonds of the scale. The cursor was initially always placed at the center of the scale (i.e., the null value). For 50% of the items, the mechanistic sentence was presented on the left side of the slider, while the mentalistic was presented on the right side. For the other 50%, the location of mechanistic and mentalistic sentences was reversed. The order of presentation of the items was randomized.

### Data Analysis and Results

All statistical analyses were performed in R (version 3.4.0, available at http://www.rproject.org).

Data analysis was conducted on a sample including responses collected from 106 participants. For each participant we calculated the *InStance Score (ISS)*. To this end, we converted the bipolar scale into a 0–100 scale where 0 corresponded to completely mechanistic and 100 to a completely mentalistic explanation. The null value of the scale, i.e., the starting position of the slider that was equally distant from both the two limits, corresponded to the 50. The ISS score was computed as the average score of all questions. Scores under 50 meant the answer was mechanistic’, scores above 50 meant they were ‘mentalistic.’

The overall average score for the ISQ was 40.73 (with 0 value indicating the most mechanistic score and 100 indicating the most mentalistic score). We tested the distribution of ISS for normality with the Shapiro–Wilk test. Results showed that Average ISS were distributed normally, *W* = 0.99, *p* > 0.05. We analyzed scores of each item of the ISQ for outliers, values that lie outside 1.5 ^∗^ Inter Quartile Range (IQR, the difference between 75th and 25th quartiles), and did not find any outlier item. In order to compare if the average ISS significantly differed from a completely mechanistic bias, we run one-sample *t*-tests against a critical value of 0 (i.e., the value corresponding to a mechanistic bond). Results showed that the average ISS significantly differed from 0, *t*(105) = 28.80, *p* < 0.0001.

**FIGURE 2:**
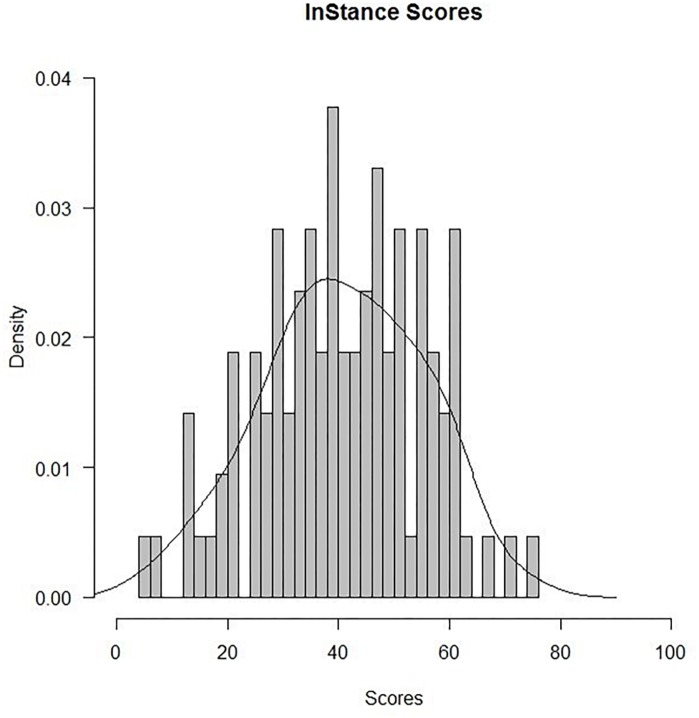
ISS distribution (individual averages, *N* = 106).

**FIGURE 3:**
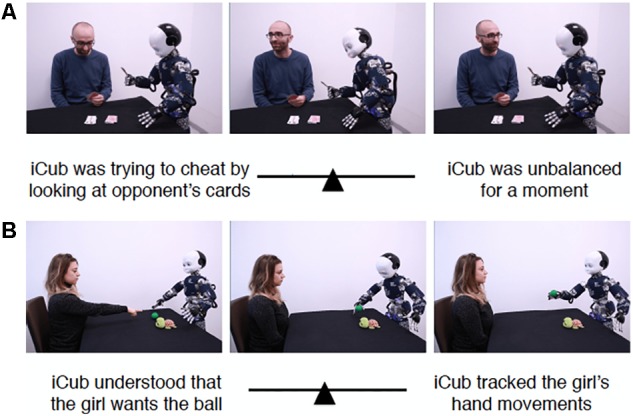
Two scenarios with highest mentalistic scores. **(A)** Shows Item 25 which received the score 78.16 on average, while **(B)** depicts Item 24 which received the score of 68.83 on average.

**FIGURE 4:**
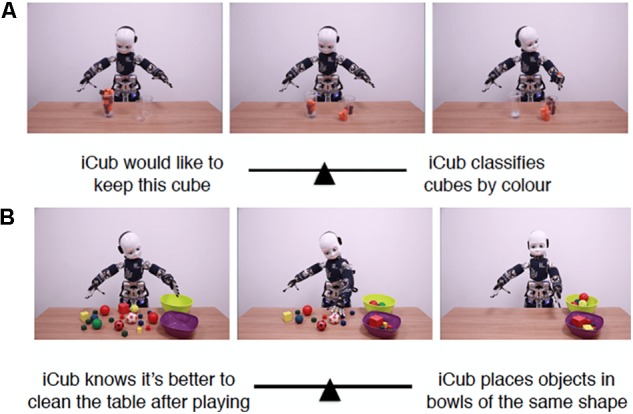
Two scenarios with highest mechanistic scores. **(A)** Shows Item 28 which received the score 10.07 on average, while **(B)** depicts Item 31 which received the score of 11.97 on average.

The average ISS for each item are summarized in Table 2; the ISS distribution of the individual averages is reported in Figure 2. Two scenarios that scored highest in mentalistic descriptions (78.16 and 68.83 on average, respectively) are presented in Figure 3; two scenarios with the most mechanistic scores (10.07 and 11.97 on average, respectively) are presented in Figure 4.

**Table 2:** Average score and standard deviation for each item of the ISQ (*N* = 106).

Item	N° humans in the scenario	Mean	*SD*	Item	N° humans in the scenario	Mean	*SD*
1	0	35.25	39.72	18	1	38.09	38.33
2	0	46.37	41.91	19	0	24.71	31.91
3	1	50.28	41.71	20	1	56.58	39.13
4	1	31.51	35.74	21	2	24.42	33.80
5	0	31.45	38.51	22	0	34.34	37.02
6	0	43.46	40.89	23	1	52.42	42.11
7	2	33.27	38.11	24	1	68.83	38.41
8	0	22.84	30.21	25	1	78.16	30.59
9	0	55.72	41.15	26	1	57.61	38.94
10	1	32.35	37.37	27	0	45.08	40.81
11	1	66.05	39.47	28	0	10.07	20.62
12	0	28.56	35.14	29	0	48.55	41.41
13	0	42.65	40.26	30	0	25.10	34.34
14	0	41.85	40.47	31	0	11.97	25.22
15	0	33.79	38.01	32	0	34.21	38.33
16	0	33.28	36.97	33	0	46.40	41.53
17	1	65.04	38.54	34	0	34.40	36.91


In order to test internal consistency of the responses to the ISQ, we calculated Cronbach’s alphas, which yielded a result of 0.83 for 34 items, indicating high internal consistency of the ISQ items. To evaluate the contribution of each item to the internal consistency of the ISQ, we run an item analysis (Ferketich, 1991). Thus, we re-estimated the α coefficient when an item was deleted. If the value of the alpha coefficient increases after the exclusion of the item, this means that the item is inconsistent with the rest of the test (Gliem and Gliem, 2003). As reported in Table 3, results of the Item Analysis clearly indicate that none of the items is inconsistent with the rest of the questionnaire.

**Table 3:** Results of the item analysis.

Item	Scale Mean if Item Deleted	Scale Variance when item Deleted	Cronbach’s Alpha when Item Deleted
1	1349.41	229722.72	0.82
2	1338.29	224066.30	0.82
3	1334.38	224588.31	0.82
4	1353.15	238581.33	0.83
5	1353.21	234277.14	0.83
6	1341.20	225788.88	0.82
7	1351.39	229548.98	0.82
8	1361.82	237512.03	0.83
9	1328.94	226282.11	0.82
10	1352.31	229355.11	0.82
11	1318.61	227443.13	0.82
12	1356.10	234238.61	0.83
13	1342.01	224117.95	0.82
14	1342.81	236687.81	0.83
15	1350.87	232598.90	0.83
16	1351.38	232130.07	0.83
17	1319.62	228192.90	0.82
18	1346.57	234567.60	0.83
19	1359.95	233798.25	0.83
20	1328.08	235541.72	0.83
21	1360.25	230697.02	0.82
22	1350.32	234925.42	0.83
23	1332.24	226723.12	0.82
24	1315.83	229252.24	0.82
25	1306.50	236004.61	0.83
26	1327.05	232772.24	0.83
27	1339.58	223211.94	0.82
28	1374.59	241566.89	0.83
29	1336.11	236128.94	0.83
30	1359.56	241206.73	0.83
31	1372.69	236999.57	0.83
32	1350.45	231757.64	0.83
33	1338.26	229756.04	0.82
34	1350.26	236259.80	0.83


To explore possible latent traits underlying the variance of the ISS, we conducted a principal-components analysis (PCA, varimax method). We assumed that the number of humans present in the scenario (one, two, or zero) might have introduced a latent factor explaining the variability of the ISS. To this end, the number of components to be extracted was limited to three. If the number of humans depicted in the scenarios represented a latent trait of the variance, then items depicting the same number of humans were expected to be significantly correlated (*r* < -0.30 or *r* > 0.30) with the same component. Results showed that the three components together accounted for only 30.34% of the variance. The variance accounted for by each component was only 13.74, 9.88, and 6.71% for component 1, 2, and 3 respectively. Moreover, as reported in Table 4 items involving the same number of humans (e.g., Items 3, 4, 10, 11, 18, 20) did not significantly score *r* < -0.30 or *r* > 0.30 on the same component. Thus, the three components are not related to the number of humans depicted in each item, and the presence or absence of humans depicted in the items is not a latent factor underlying the variance of our data.

**Table 4:** Correlation scores of the InStance items with the three components.

	Component				Component		
			
Item	1	2	3	Item	1	2	3
1	-0.393	0.448	0.451	18		0.402	
2	0.348	-0.686		19	0.369		
3			-0.534	20	0.437		
4	0.318	-0.318		21	0.278	0.377	
5	0.512			22		0.261	
6	0.582			23	0.363		
7	0.268	0.381	-0.400	24		0.359	0.450
8	0.360			25	0.431	0.278	
9	0.266			26	0.345		
10	0.440	0.251		27	0.524	-0.380	
11	0.338	0.319	-0.288	28	0.522	-0.262	
12	0.413			29	0.482		
13	0.311			30	0.339	0.259	
14	0.605			31		0.677	0.310
15		0.506	-0.463	32	0.265		0.549
16	0.628			33		0.252	0.432
17		0.434	-0.255	34		0.385	


*Extraction Method: Principal Component Analysis, Varimax Rotation. Scores between -0.25 and 0.25 are not reported*.

We evaluated the associations between the ISS scores and gender, age, education, number of children, and siblings of the respondents using multiple linear regression. Results showed no significant associations between the ISS and the respondents’ characteristics of age, gender, education, number of children, or siblings (all *p*s > 0.32). To assess the relationship between the ISS and scores in Reading the Mind in the Eyes and Völlm questionnaires, Pearson product-moment correlation coefficients were calculated. Results showed no correlation between the ISS and the scores in the Reading the Mind in the Eyes or Völlm questionnaires (all *p*s > 0.06).

From 106 respondents, 84% reported that they were completely unfamiliar with robots (*N* = 89) while the remaining 16% reported various levels of familiarity (*N* = 17). In order to check whether the ISS were associated with the degree of familiarity of the respondents with humanoid robots, we performed linear regression analysis. No effect of familiarity with robots on the ISS was observed, *p* = 0.21. The average scores for the ISQ were 36.69 and 41.50 for familiar and not familiar with robots respondents, respectively. The average ISS significantly differed from the critical value of 0 (i.e., the value associated with a completely mechanistic bias), *t*(16) = 9.79, *p* < 0.0001 and *t*(88) = 29.30, *p* < 0.0001, for the familiar and not familiar with robots respondents, respectively.

### Analyses on the Group Not Familiar With Robots

**FIGURE 5:**
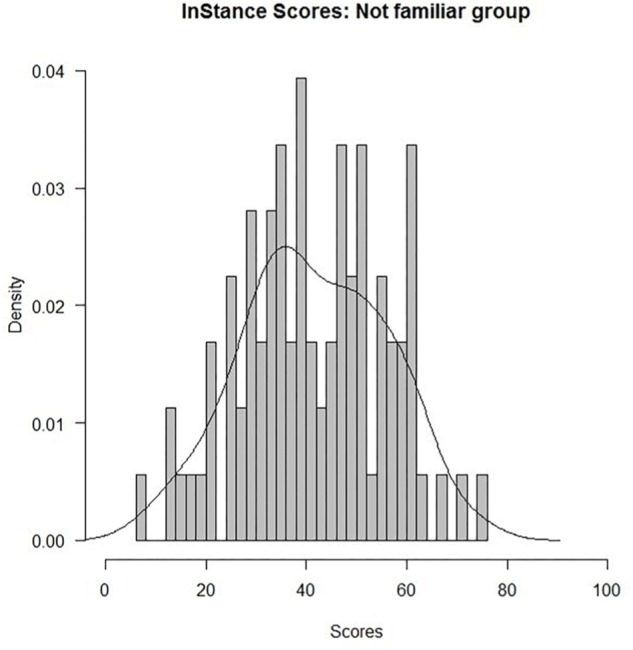
ISS (individual averages) distribution for the not familiar group (*N* = 89).

In addition to the analyses of data from the entire sample, we conducted further analyses on data from participants who reported no familiarity with robots. As outlined above, our main aim was to investigate the likelihood of adopting the intentional stance for a given humanoid iCub in the general population – that is, people with no previous experience with robots. For the group of respondents who were not familiar with robots, the ISS were also distributed normally (Figure 5), Shapiro–Wilk test: *W* = 0.99. *p* > 0.05.

**FIGURE 6:**
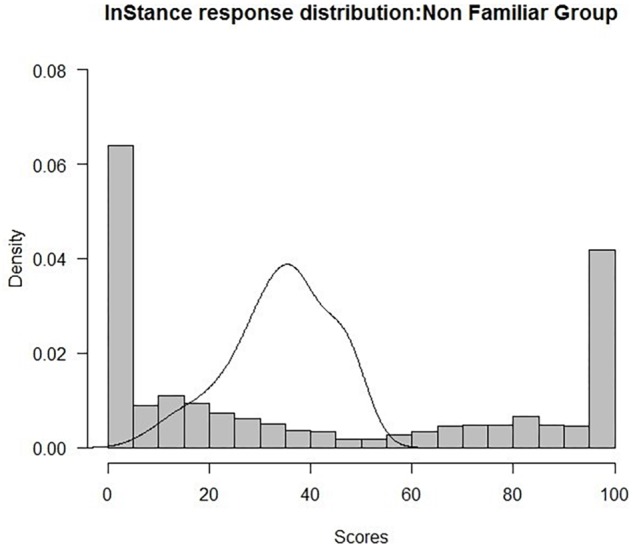
Plot of raw data for the not familiar group (*N* = 89, Shapiro–Wilk test: *W* = 0.81 *p* < 0.001).

Results also showed no significant associations between the ISS and the respondent characteristics of age, gender, education, number of children, and siblings, for the group of respondents who were not familiar with robots (all *p*s > 0.16). Similarly, no correlations between the ISS and the scores in the Reading the Mind in the Eyes and Völlm et al. (2006) questionnaires were found (all *p*s > 0.16). In order to explore if responses to the questionnaire were polarized, we conducted a polynomial curve-fitting analysis on the density of the *raw* scores. Results showed a significant quadratic trend (*t* = 5.59; *p* < 0.001, *R*^2^ = 0.83), supporting the polarization hypothesis (Figure 6).

**FIGURE 7:**
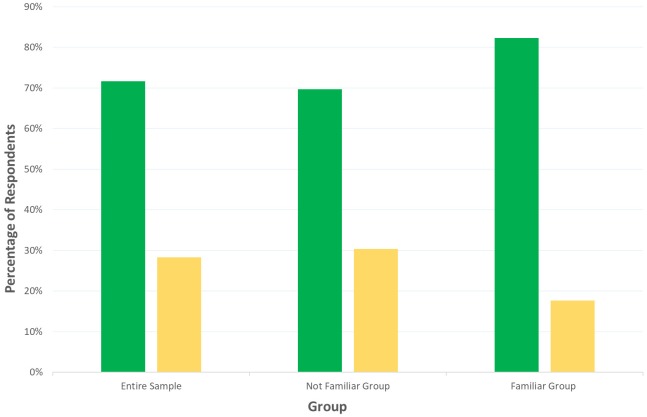
Percentage of Mechanistic (green bars) and Mentalistic (yellow bars) respondents for the entire sample (*N* = 106) on the left, the Not Familiar group (*N* = 89), and the Familiar group (*N* = 17) on the right.

In addition, we estimated the percentage of participants who attributed ‘mechanistic’ or ‘mentalistic’ descriptions according to their average ISS. Participants were classified into two groups according to their ISS. Participants who scored below 50 (0 – 50 in our scale) were assigned to the Mechanistic group (*N* = 62), whereas participants with an ISS above 50 (50 – 100) were classified as the Mentalistic group (*N* = 27). To check whether the percentage of respondents in the Mechanistic and Mentalistic group differed from chance level (i.e., expected frequency of 0.5), we performed a chi-square test. Results revealed that the frequency of participants who scored Mechanistic (69.7%) and the frequency of participants who scored Mentalistic (30.3%) were both different from the chance level, *X*^2^(1. *N* = 89) = 13.76. *p* < 0.001, Figure 7. In order to compare if the mean ISS of the two groups (Mechanistic and Mentalistic) significantly differed from the null value of our scale (i.e., 50, which corresponded to the position at which the slider was equally distant from both statements), we run one-sample *t*-tests against a critical value of 50 (i.e., the null value of our scale). Results showed that the mean ISS significantly differed from the null value of 50 both for the Mechanistic [*M* = 34.19, SEM = 1.28, *t*(61) = -12.33, *p* < 0.0001] and the Mentalistic group [*M* = 58.27, SEM = 1.18, *t*(26) = 7.03, *p* < 0.0001].

## General Discussion

The aim of the present study was to develop a method for assessing whether humans would sometimes (at least in some contexts) adopt the intentional stance toward the humanoid robot iCub. To meet this aim, we developed a questionnaire (Intentional Stance Questionnaire, ISQ) that consisted of 34 items. Each item was a sequence of three photographs depicting iCub involved in a naturalistic action. Below each sequence, there was a slider scale on which participants could move the cursor in the direction of one of the extremes of the scale. On one of the extremes, there was a mentalistic description of what was depicted in the photographs, on the other extreme there was a mechanistic description. We administered the ISQ online and received responses from 106 participants. The high internal consistency (α = 0.83) of mean scores (Intentional Stance Scores, ISS) of the items revealed that the questionnaire we developed was uniform. This is consistent also with the result of the principal component analysis (PCA). Specifically, no differences were found between “social” (iCub interacting with one or two other agents) and “individual” (iCub alone) scenarios. Thus, we can conclude that our tool is reliable.

No correlation was found between sociodemographic characteristics of participants and ISS. Adopting the intentional or design stance was not affected by socioeconomic factors (i.e., education) for our questionnaire. Furthermore, we found no correlation with the two tests used for the assessment of ToM abilities, namely the Reading Mind in the Eyes (Golan et al., 2006) and the Völlm et al. (2006) comic-strip questionnaire. The main reason behind this might be that error rates in the healthy adult population in these two tests are typically very low, and, in our sample, participants were at ceiling performance and there was almost no variance in terms of accuracy. Arguably, testing psychiatric population could lead to a higher variance of accuracy between participants, and could provide additional information regarding the relationship between ToM abilities and adoption of intentional stance toward non-human agents. Importantly for our purposes, however, using these tests showed that we did not have any outliers in terms of ToM abilities.

Overall, our results indicate that participants showed a slight bias toward a mechanistic explanation when they were asked to evaluate robot actions (mean overall score = 40.73). This might be due to the fact that participants were observing a robot agent. This is also in line with previous studies emphasizing that a lower degree of intentionality is attributed to machines when explaining their actions, while a higher degree is attributed when perceiving conspecifics’ behaviors (Krach et al., 2008; Chaminade et al., 2010; for review, see Wiese et al., 2017). The results are also in line with the essence of the concept of the intentional stance (Dennett, 1987). We can speculate that, in order to understand a behavior of a robot, humans are more prone to adopt the design, rather than the intentional, stance – and this is in spite of the natural tendency to anthropomorphize unknown entities (Epley et al., 2007). Perhaps nowadays, humanoid robots are not unknown entities anymore: in the Western world, some of them are already used at airports, shops or cultural events to provide information to visitors (Aaltonen et al., 2017; for a review see Mubin et al., 2018).

However, and interestingly, the design stance descriptions were not always chosen in order to explain iCub’s actions, as the average score was significantly different from zero. This clearly indicates that participants have at times also chosen mentalistic explanations of the given scenarios. This choice could have depended on specific scenarios (some were more likely to be interpreted mentalistically than mechanistically), or also on individual differences among participants (there was a sub-sample that was more likely to adopt the intentional stance, and a sub-sample that was more likely to adopt the design stance). This shows that in principle it might be possible to induce adoption of intentional stance toward artificial agents. The likelihood of adopting the intentional stance might depend on the context in which the robot is observed, its behavioral characteristics (e.g., contingency of its behavior on participant’s behavior, cf. Willemse et al., 2018), cultural background (attitude toward humanoid robots is strongly associated with culture (for a review see Haring et al., 2014) and also individual differences of participants.

In order to further investigate potential factors contributing to the choice between the mentalistic vs. mechanistic rating, we asked participants whether they had previous experience with robots. Our data did not show any effect of familiarity with robots (*p* > 0.05) on the ratings, but it is important to point out that the majority of our sample consisted of people not familiar with this kind of technology (*N* = 89). To analyze a more homogenous sample with respect to familiarity with robots, we conducted follow-up analyses only on the sample not familiar with robots.

By analyzing the differences in scores between items in the “non-familiar” group, we noticed that some scenarios strongly elicited a mentalistic explanation. Interestingly, when we examined mentalistic (*M* = 58.27) and mechanistic (*M* = 34.19) evaluations between participants, we found that both types of scores were significantly different from the null value of our scale (for both, one-sample *t*-test revealed significant difference from 50, *p* < 0.001). Together with the results implying polarization in the scores (Figure 6), this suggests that participants were clearly choosing for each scenario either a mechanistic or a mentalistic explanation, neither answering randomly nor relying on the middle point of the scale. Davidson (1999) has proposed one possible explanation for this phenomenon. The author pointed out that humans possess many types of vocabulary for describing nature of mindless entities, and for describing intentional agents, but might lack a way of describing what is between the two (Davidson, 1999). This is also in line with Dennett’s proposal (Dennett, 1981) of intentional stance: when we find a model that is most efficient to explain a behavior, we take its prototypical explanation, and do not necessarily search for explanations that are in the fuzzy zones of in-between models. In each scenario of our questionnaire, the robot was always the same, but the action and the environment/context around it changed across conditions, modulating participants’ ratings. However, the rating became quite polarized once there was a bias toward either the mentalistic or the mechanistic explanation. This might be a consequence of a general tendency of people to form discrete categories rather than continuous fuzzy concepts (Dietrich and Markman, 2003) but it does also suggest the existence of a certain degree of flexibility that allows humans to shift between such categories. Based on our results, we can argue that the adoption of such mentalistic or mechanistic models does not rely only on intrinsic properties of the agent, but also on contingent factors (Waytz et al., 2010) and on the observer’s dispositions (Dennett, 1990), in a similar way as it occurs for human-likeness and anthropomorphism (Fink, 2012).

### Limitations

Despite the novelty of our questionnaire, some level of caution needs to be assumed when interpreting the results. The main aim of our study was to develop a tool for exploring whether humans would sometimes adopt the intentional stance toward a specific robot iCub. Therefore, for each scenario, we created two alternative explanations equally plausible (and equally ambiguous so that one would not be chosen over the other due to its higher level of accuracy in description). We asked 14 volunteers (with philosophy background) to rate whether the sentences we created were falling in the mechanistic or mentalistic category as we intended them to be. The final version of our questionnaire demonstrated high internal consistency, but a deeper analysis of the effective ambiguity between the proposed sentences is needed. When dealing with questionnaires, items are often open to multiple interpretations, which might change from one individual to another. Future studies should address how individual differences affect the interpretation of our items. Similarly, some scenarios we created might appear difficult to interpret. It could therefore be argued that the coherence of the story lines might affect participants’ ratings. However, the item analysis demonstrated that there is no significant difference between items in terms of reliability, thereby reassuring that items were uniformly coherent. Nevertheless, a future investigation on internal coherence of the scenarios might be needed in order to apply the ISQ to social robotics research.

One further limitation could be the lack of possibility to design a proper control condition. A control condition with a human agent is almost impossible, as mechanistic descriptions of a human behavior are very unnatural. It is very strange to provide participants with mechanistic descriptions of human behavior, for example, descriptions such as “calibration of motors,” and it might even contradict the key concept of intentional stance. On the other hand, modifying descriptions to make them more plausible for human agents would not provide a proper control. In fact, our attempt of designing a control condition with a human agent (see Supplementary Materials) showed that participants found the task very strange, and they experienced the agent as robotic and unnatural (open comments after completion of the questionnaire). What could have happened in this case is that presumably the depicted human agent became dehumanized due to the restrictions on the expressiveness and posture (to match the robot condition) and due to the mechanistic descriptions.

On the other hand, a non-anthropomorphic control condition would not be viable, as many mentalistic descriptions of ISQ actually refer to the behavioral repertoire that is human-like (gaze direction, being “surprised to see”). A non-anthropomorphic comparison should not have any human-like features (e.g., eyes). Without eyes, however, such descriptions are senseless.

However, comparing the intentional attributions toward a humanoid robot to a human agent or to a non-anthropomorphic robot was not the aim of the present study. We did not intend to ask the question of whether the degree of intentional stance adopted to a humanoid robot would be comparable with respect to another type of agent (human or non-anthropomorphic robot). The aim of our study was to examine if people sometimes adopt the intentional stance toward humanoid robots, and the results of our questionnaire showed that this is indeed the case.

First step toward a comparison between a robot and a human agent in terms of adoption of intentional stance was made by Thellman et al. (2017). The authors designed a study in which they presented a series of images and verbal descriptions of different behaviors exhibited either by a person or by a humanoid robot. Verbal statements described an outcome, event, action or state either in positive or in negative terms (i.e., “Ellis makes a fantastic cake” or “Ellis burns the cake”). Participants were asked to rate the intentionality, controllability and desirability of the behavior, and to judge the plausibility of seven different types of explanations (derived from a psychological model of lay causal explanation of human behavior, Malle and Knobe, 1997). Results showed that the scores were very similar for humans and robots, meaning, people explained the behavior of both agents in terms of mental causes. Interestingly, participants were also asked to rate how confident they were with their score. The confidence ratings revealed lower confidence when rating robot behavior relative to rating human behavior. This shows that despite explaining the behavior of the robot in intentional terms, such interpretation perhaps caused some degree of cognitive dissonance. Further studies might need to explore more in depth on the origins of such effects.

Our study is, however, somewhat different from the study of Thellman et al. (2017), as it provided participants with the direct choice of mentalistic and mechanistic explanations of robot behavior, thereby probing the adoption of intentional stance, and contrasting it with design stance more directly. Moreover, as argued above, we did not aim to compare the degree of adoption of the intentional stance toward a humanoid robot, with respect to another human. Finally, we also did not aim at answering the question of whether anthropomorphic physical appearance of the robot plays a role in adoption of intentional stance (in this case, a comparison with a non-anthropomorphic robot would be needed). Our aim was solely to present a tool that we developed, and explore whether humans would at times choose a mentalistic description/interpretation of behaviour of a humanoid robot.

### Future Directions

In general, it is of interest – not only for theoretical but also practical reasons – to ask whether adoption of intentional stance is a factor in social acceptance. Robots are considered as future ***assistive*** technologies, with potential applications from healthcare to industry. However, some studies brought to light potential issues with acceptance of artifacts in the human social environments (Bartneck and Reichenbach, 2005). Furthermore, pop-culture has induced a substantial amount of skepticism toward robots, associating them with threats for humanity (Kaplan, 2004). For these reasons, it is crucial to understand what factors contribute to acceptance of robots in human environments, and whether adoption of the intentional stance is one of those factors. It has been argued that robots, and specifically humanoids, have the potential to trigger attribution of mental states, as long as they display observable signs of intentionality, such as human-like behaviors (Wykowska et al., 2014, 2015a,b) and appearance (i.e., biological motion or human-like facial traits, Chaminade et al., 2007, 2012; Wiese et al., 2012, 2017; Özdem et al., 2017).

## Conclusion

In summary, the present study used a novel method to explore whether the intentional stance is at times adopted toward a humanoid robot iCub. Our results show that it is possible to induce adoption of the intentional stance toward the robot at times, perhaps due to its human-like appearance. Further research needs to explore what are the exact factors (individual difference, cultural context, specific characteristics of robot appearance or behavior) that influence the adoption of intentional stance.

## Author Contributions

SM, DG, FC, EB, and AW designed the study. SM and JP-O developed the experimental materials. EB, FC, and DG analyzed the data. SM, DG, FC, JP-O, and AW wrote the manuscript.

## Conflict of Interest Statement

The authors declare that the research was conducted in the absence of any commercial or financial relationships that could be construed as a potential conflict of interest.

## Footnotes

1 www.soscisurvey.de
