# Supplementary material for: Do We Adopt the Intentional Stance Toward Humanoid Robots?
Source: Front Psychol. 2019 Mar 15;10:450. doi: 10.3389/fpsyg.2019.00450 (PMC6428708; doi:10.3389/fpsyg.2019.00450)
Supplement: Supplementary file 1 [file Presentation_1.pdf]

Supplementary Material 1

Do we adopt the Intentional Stance toward humanoid robots?

Serena Marchesi[1,3], Davide Ghiglino[1,2], Francesca Ciardo[1], Jairo Perez-Osorio[1], Ebru Baykara[1], Agnieszka Wykowska[1]*

[1] Social Cognition in Human-Robot Interaction unit, Istituto Italiano di Tecnologia, Genoa, Italy

[2]DIBRIS, Università di Genova

[3] School of Computer Science, Faculty of Science and Engineering, Manchester University, Manchester, UK

*Corresponding author:

Agnieszka Wykowska

Istituto Italiano di Tecnologia

Centre for Human Technologies

Via E. Melen, 83

16040 Genova, Italy

e-mail: agnieszka.wykowska@iit.it

Human control experiment

Materials and methods

Sample

First, we collected data of one hundred and twenty Italian native speakers with different social and educational backgrounds (see Table 1 for demographical details) who completed our Human Control Questionnaire (HCQ): HCQ mapping not-counterbalanced, N=120). Due to a mistake in counterbalancing the position (left vs. right) of mechanistic or mentalistic descriptions, we administered the HCQ with the proper counterbalanced mapping to a second sample of hundred and three participants, from which we excluded one participant who already filled in the first HCQ: HCQ mapping counterbalanced, N=102. Data collection was conducted in accordance with the ethical standards laid down in the Code of Ethics of the World Medical Association (Declaration of Helsinki), procedures were approved by the regional ethics committee (Comitato Etico Regione Liguria).

Table 1**.** Demographic details of the sample (N=222).

Demographic Characteristic	
Age (years), mean (SD) [min, max]	29.81 (10.39) [18,68]
Female, n (%)	153 (68.92)
Education (years), mean (SD) [min, max]	16.20 (2.97) [8, 24]

Human Control Questionnaire (HCQ)

We selected 15 scenarios out of the 34 original ones that could be adapted to a human agent with respect to the mechanistic descriptions (we excluded all items that used very implausible mechanistic descriptions such as motor calibration) and digitally edited them (Adobe

Photoshop CC 2018) depicting a human agent (Paola) instead of the iCub robot. Each item of the HCQ was identical with the ISQ, except for the depicted agent in the scenarios, which for HCQ was a human (see Figure 1 for an example, but see also Supplementary Material 3 for all the scenarios included in the HCQ). Each scenario was composed of three pictures (size 900 x 173.2 pixels). Out of the 15 scenarios, 9 involved one (or more) other human interacting with Paola; 1 scenario showed a human arm pointing to an object; 6 scenarios depicted only Paola. The types of action performed by Paola in the scenarios were: grasping, pointing, gazing, and head movements.

As in the original ISQ, each item included two sentences, in addition to the scenario. One of the sentences was always explaining Paola's behaviour referring to the design stance (i.e., mechanistic explanation), whereas the other was always describing Paola's behaviour referring to mental states (i.e., mentalistic explanation). We kept the human agent's emotional expression constant within and across the scenarios not to bias towards mentalistic explanations, see Supplementary Material 3 for a complete list of items.

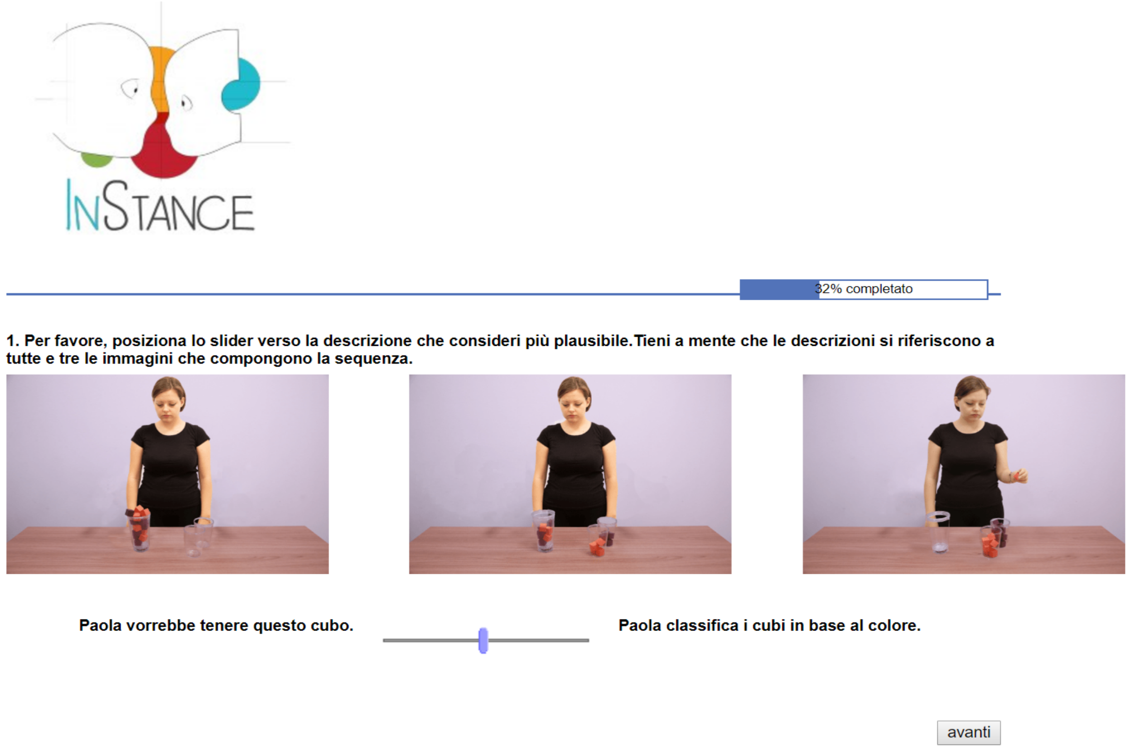

Figure 1. Screenshot from the Human Control Questionnaire in Italian.

Data Analysis and Results.

All statistical analyses were performed in R (version 3.4.0, freely available at http://www.rproject.org). Data analysis was conducted in three steps. First, we analysed responses in the HCQ as we did for the InStance questionnaire (ISQ). Second, we selected from our original ISQ only responses to the same 15 items included in the HCQ, and we performed analyses on those. Finally, we compared responses in the two questionnaires (15 items) between groups.

Human control questionnaire (HCQ)

For each participant, we calculated the average score. As we did for the ISQ, we converted the bipolar scale into a 0-100 scale where 0 corresponded to completely mechanistic and 100 to a completely mentalistic explanation. The null value of the scale, i.e. the starting position of the slider that was equally distant from both limits, corresponded to 50. Scores under 50 meant the answer was ‘mechanistic’, scores above 50 meant they were ‘mentalistic’. Firstly, we investigated the effect of the mapping by comparing the average score across groups (i.e. not-counterbalanced mapping sample vs. counterbalanced mapping sample). Independent sample t-test showed that the average score did not differ across groups, $t(220) < 1$. Given that no differences were found between mappings, from the total sample of N=222 participants, we randomly selected 106 respondents, to match the sample size of our original ISQ. The overall average score for the HCQ was 54.62 (with 0 value indicating the most mechanistic score and 100 indicating the most mentalistic score). We tested the distribution of the average score for normality with the Shapiro–Wilk test. Results showed that the average scores were distributed normally, $W = 0.99$, $p > .05$. Moreover, in order to check that the average score for the HCQ was not the result of random choice, we conducted one-sample t-tests against a critical value of 50 (i.e. the position at which the slider was equally distant from both statements). Results

showed that the average score significantly differed from 50, t (105) = 3.67, $p < .001$). Then, as we did for the ISQ, we focused only on respondents who were not familiar with robots. From the original sample of N=222, we selected randomly N= 89 respondents (to match the sample of the non-familiar group in ISQ), who reported no familiarity with robots. The average score was 54.82, and it was distributed normally, Shapiro–Wilk test: W = 0.97, p = .052. Then, as we did for the ISQ, we estimated the percentage of participants who attributed 'mechanistic' or 'mentalistic' descriptions according to their average score. Participants who scored below 50 (0 - 50 in our scale) were assigned to the Mechanistic group (N=32), whereas participants with an average score above 50 (50 – 100) were classified as the Mentalistic group (N=56). To check whether the percentage of respondents in the Mechanistic and Mentalistic group differed from chance level (i.e., expected frequency of 0.5), we performed a chi-square test. Results revealed that the frequency of participants who scored in HCQ "Mechanistic" (36.4 %) and the frequency of participants who scored in HCQ "Mentalistic" (63.4 %) were both different from the chance level, X^2 (1. N=88) = 51.26, p < .001. In order to compare if the average scores of the two groups (Mechanistic and Mentalistic) significantly differed from the null value of our scale (i.e. 50, which corresponded to the position at which the slider was equally distant from both statements), we ran one-sample t-tests against a critical value of 50 (i.e. the null value of our scale). Results showed that the average score significantly differed from the null value of 50 both for the Mechanistic (M =42.95, SD = 7.48, t (31) = -5.33, $p < .001$) and the Mentalistic group (M =61.69, SD = 9.60, t (55) = 9.11, $p < .001$).

InStance questionnaire: selected 15 items

From the original ISQ, we selected responses to the same 15 items as those included in the HCQ. The average InStance score (ISS) was 38.32, and it was distributed normally, Shapiro–Wilk test: W = 0.98, p > .05. In order to compare if the ISS significantly differed from a

completely mechanistic bias, we conducted one-sample t-tests against a critical value of 0 (i.e. the value corresponding to a mechanistic bond). Results showed that the average ISS significantly differed from 0, t (105) = 24.52, $p < .001$). For respondents who were not familiar with robots (N = 89), the average score was 38.67, and it was distributed normally, Shapiro–Wilk test: W = 0.98, p > .05. Then, as we did for the full version of the ISQ, we estimated the percentage of participants who attributed 'mechanistic' or 'mentalistic' descriptions according to their average score. Participants who scored below 50 (0 - 50 in our scale) were assigned to the Mechanistic group (N=65), whereas participants with an average score above 50 (50 – 100) were classified as the Mentalistic group (N=24). To check whether the percentage of respondents in the Mechanistic and Mentalistic categories differed from chance level (i.e., expected frequency of 0.5), we conducted a chi-square test. Results revealed that the frequency of participants who scored in ISQ "Mechanistic" (73.0 %) and the frequency of participants who scored in ISQ "Mentalistic" (27.0 %) were both different from the chance level, X^2(1. N =89) = 18.89, p < .001. In order to compare if the mean average scores of the two groups (Mechanistic and Mentalistic) significantly differed from the null value of our scale (i.e. 50, which corresponded to the position at which the slider was equally distant from both the two statements), we conducted one-sample t-tests against a critical value of 50 (i.e. the null value of our scale). Results showed that the average score significantly differed from the null value of 50 both for the Mechanistic (M =31.19 SD = 11.15, t (64) = -13.61, $p < .001$) and the Mentalistic group (M =58.96, SD = 6.08, t (23) = 7.22, $p < .001$).

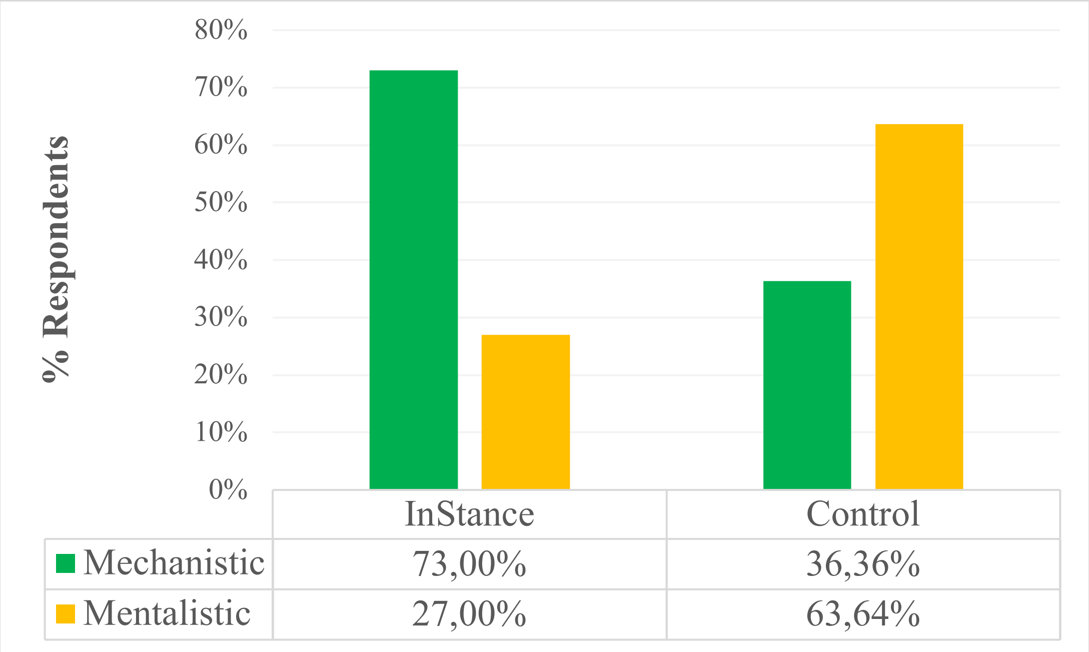

Figure 2. Percentage of Mechanistic (green bars) and Mentalistic (yellow bars) respondents to the reduced version of the ISQ on the left, and to the HCQ on the right, for participants who were not familiar with robots (N=89).

Comparison between InStance and Human control questionnaire

Average scores for ISQ and HCQ for 15 items are shown in Table 2. In order to compare if the average score for the HCQ statistically differed from the ISS, we conducted an independent-samples t-test. Results showed that the ISS for the shorter version of our InStance questionnaire (M= 38.32, SD= 16.09) significantly from the average score of the HCQ (M= 54.62, SD= 12.95), t(210) = 8.12, p < .001, d = 1.12. Similar results were found when considering only respondents who were not familiar with robots (N= 89), t(176) = 7.5, p < .001, d = 1.12.

Table 2. Average score and standard deviation for each item of the ISQ and HCQ (N= 106).

**Item ISQ**	**N° humans in the scenario**	**Mean**	**SD**	**Item HCQ**	**N° humans in the scenario**	**Mean**	**SD**
**3**	1	50.28	41.71	**3**	1	52.05	41.61
**4**	1	31.51	35.74	**4**	1	51.47	42.90
**8**	0	22.84	30.21	**8**	0	42.25	40.90

**10**	1	32.35	37.37	**10**	1	45.61	40.99
**11**	1	66.05	39.47	**11**	1	74.92	36.36
**13**	0	42.65	40.26	**13**	0	56.47	42.87
**16**	0	33.28	36.97	**16**	0	51.97	41.55
**18**	1	38.09	38.33	**18**	1	66.82	37.46
**21**	2	24.42	33.80	**21**	2	35.99	42.48
**22**	0	34.34	37.02	**22**	0	59.30	41.65
**23**	1	52.42	42.11	**23**	1	78.22	33.44
**25**	1	78.16	30.59	**25**	1	72.58	37.44
**28**	0	10.07	20.62	**28**	0	43.53	45.63
**31**	0	11.97	25.22	**31**	0	53.76	44.95
**33**	0	46.40	41.53	**33**	0	34.33	40.75

The InStance Questionnaire Supplementary material 2 ENGLISH

Item 0 (Example)

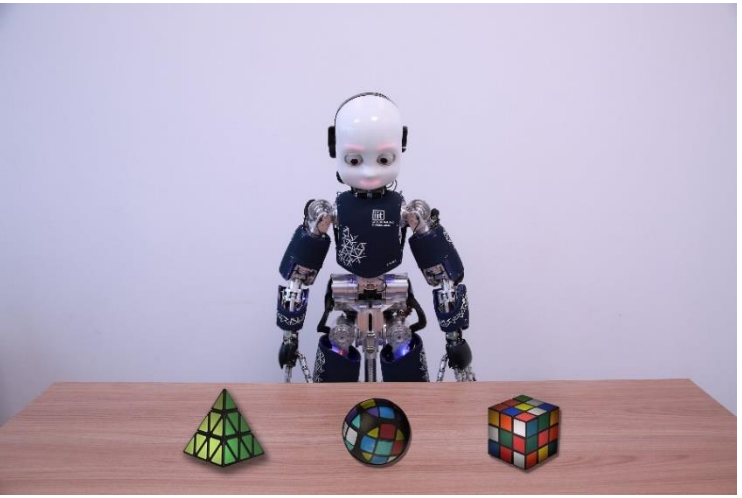

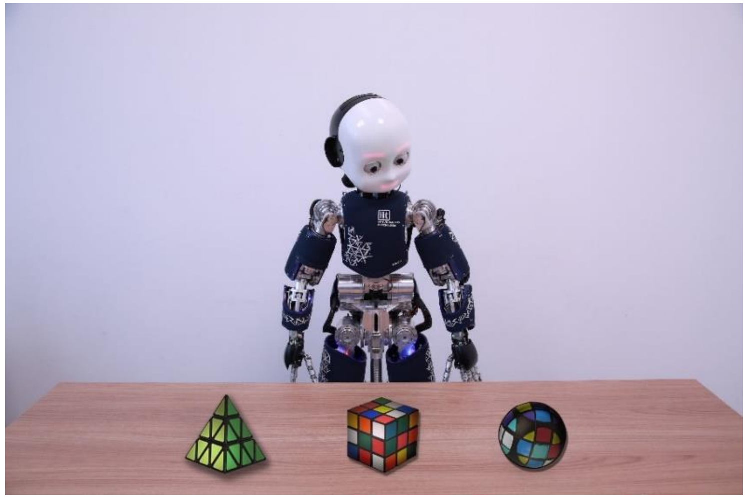

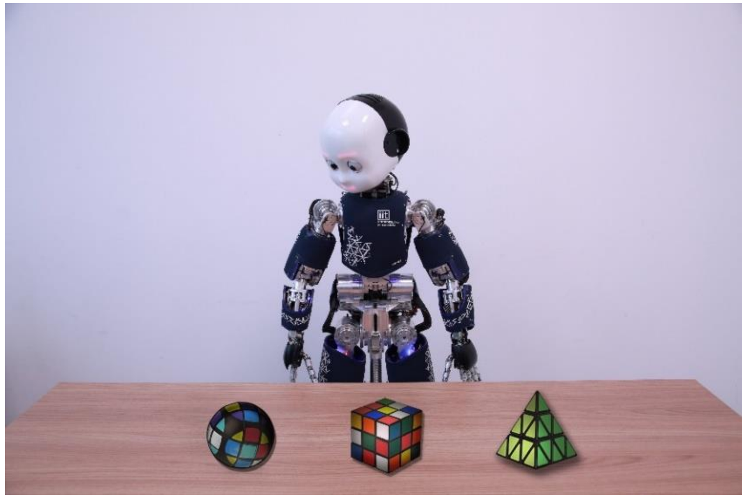

iCub categorizes objects by their shapes.

iCub likes round objects.

M= 55.08; SD: 44.39

Item 1

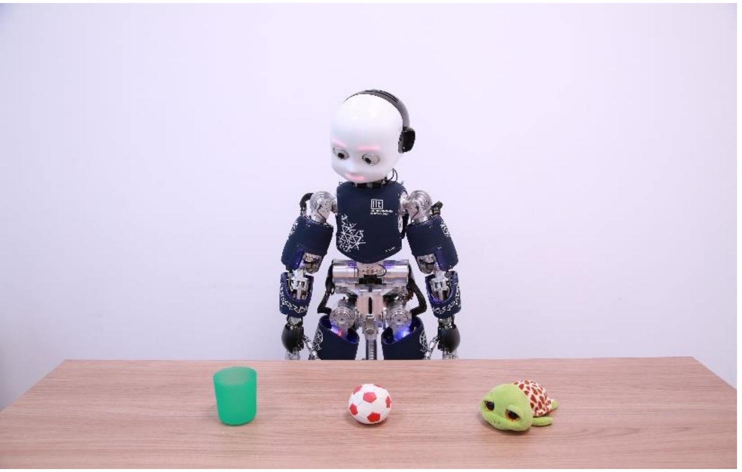

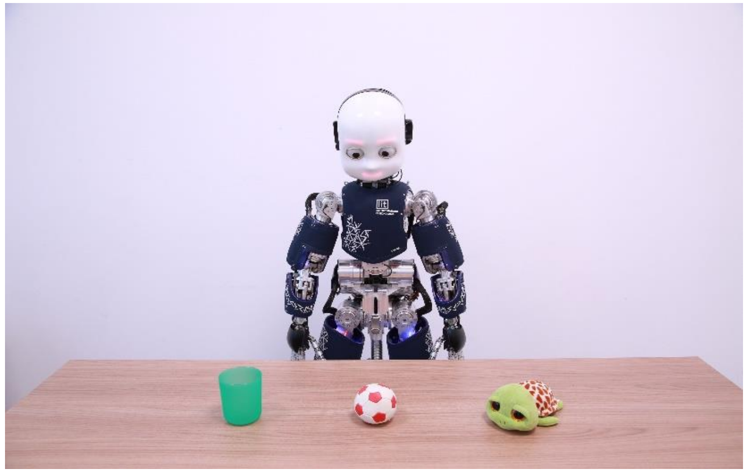

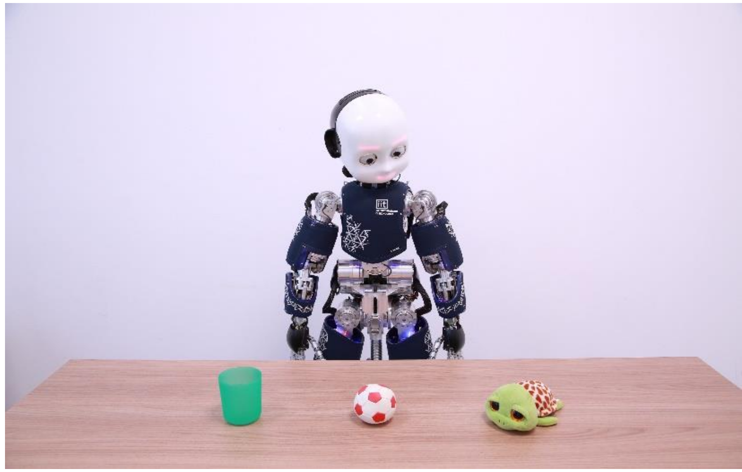

iCub is scanning the environment.

iCub is interested in these objects.

M= 35.25; SD: 39.72

Item 2

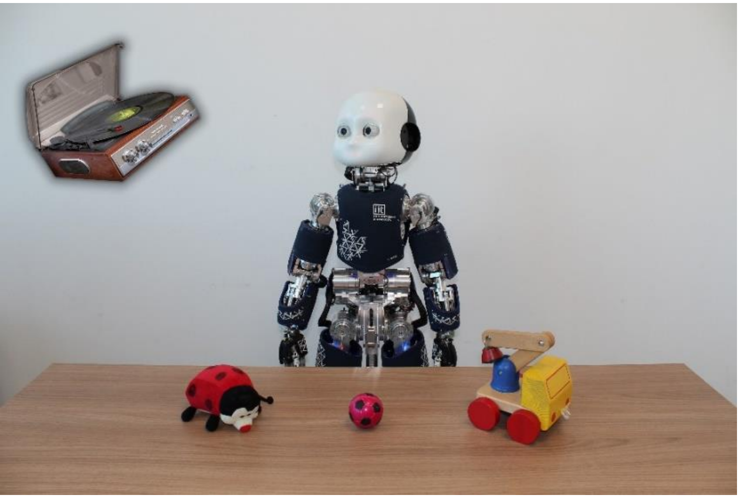

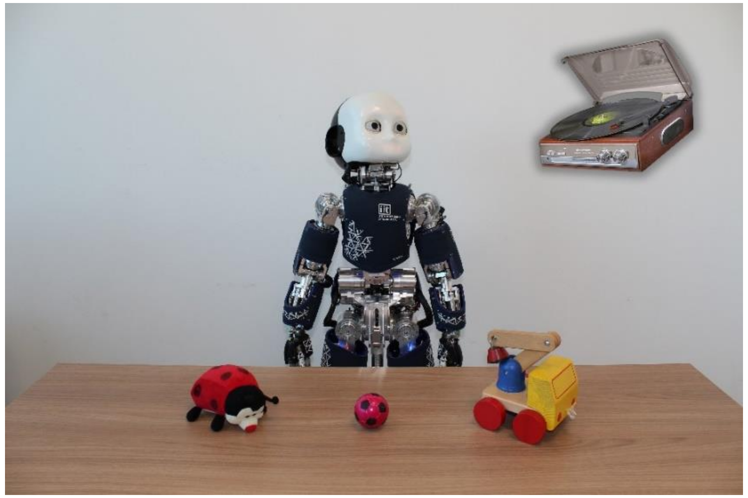

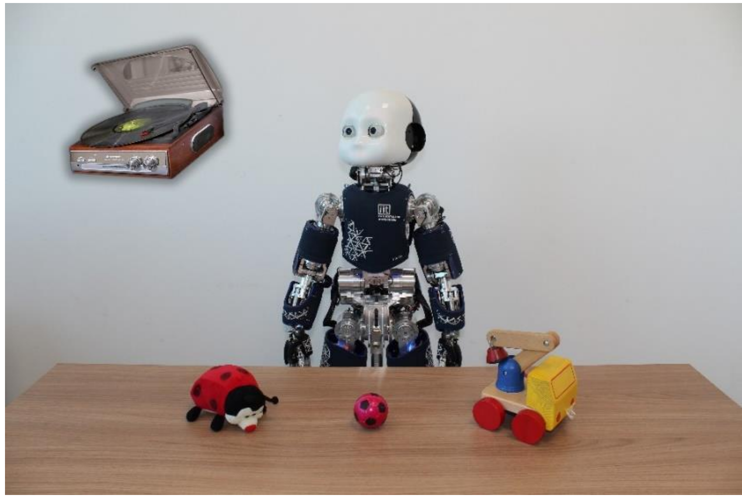

iCub looks at objects at eye level.

iCub is surprised to see the object in the air.

M= 46.37; SD: 41.91

Item 3

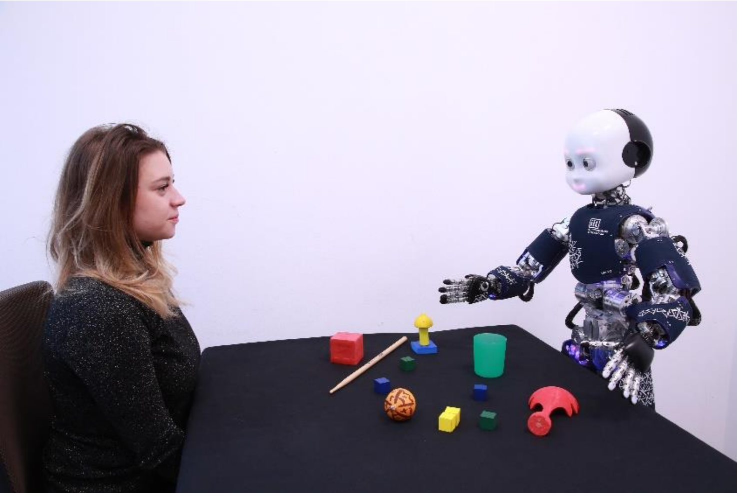

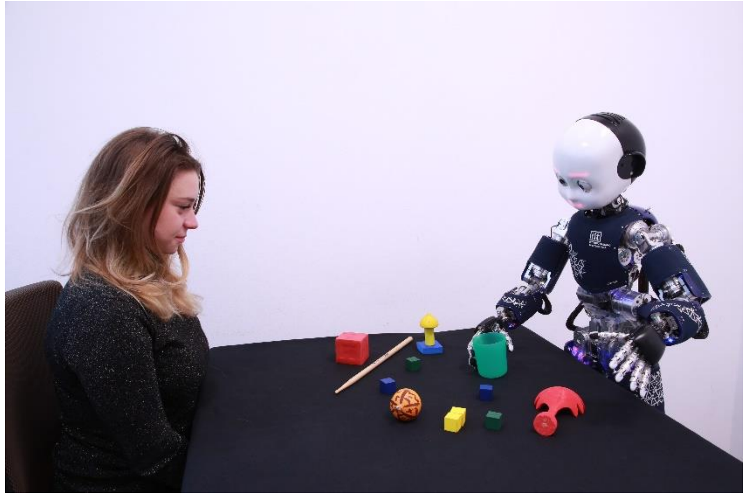

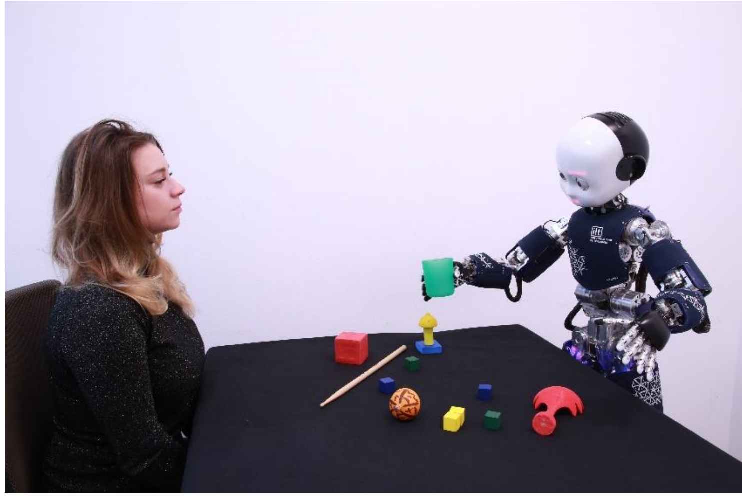

iCub grasps cylindrical objects best.

iCub believes that the girl likes the cup.

M= 50.28; SD: 41.71

Item 4

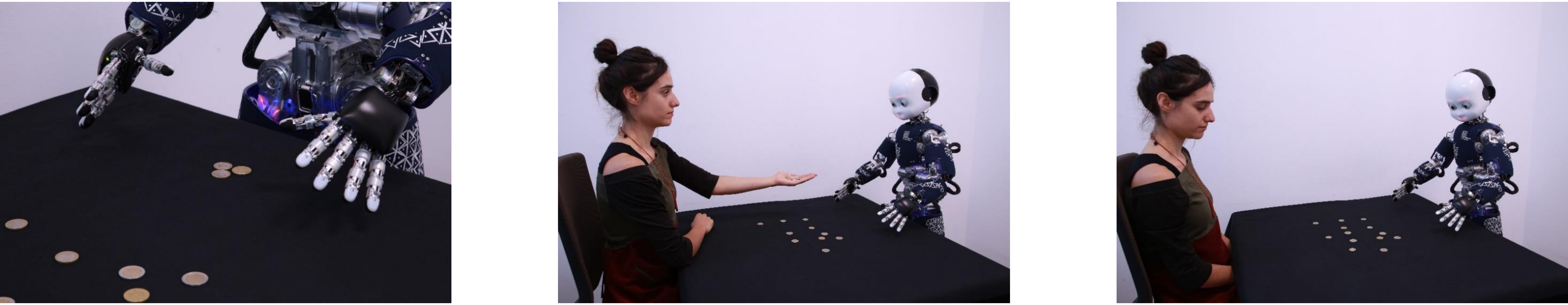

iCub is calculating the number coins.

iCub decided to give back the money.

M= 31.51; SD: 35.74

Item 5

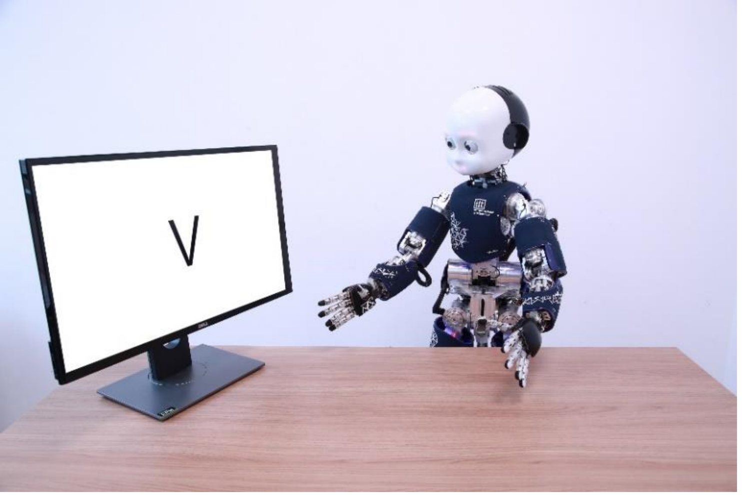

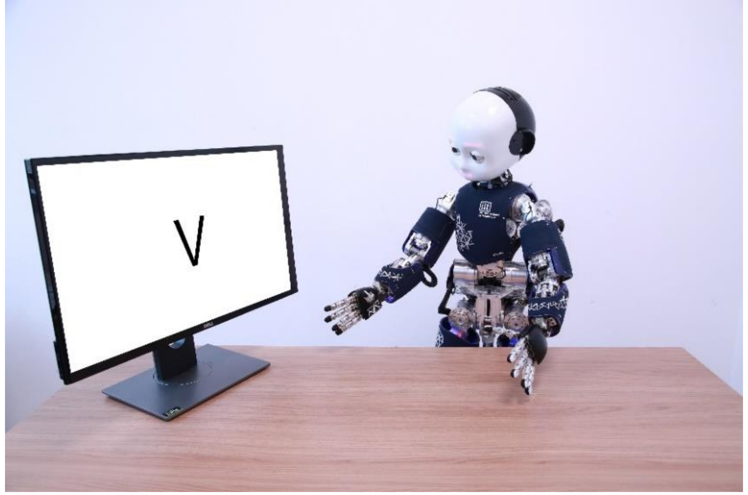

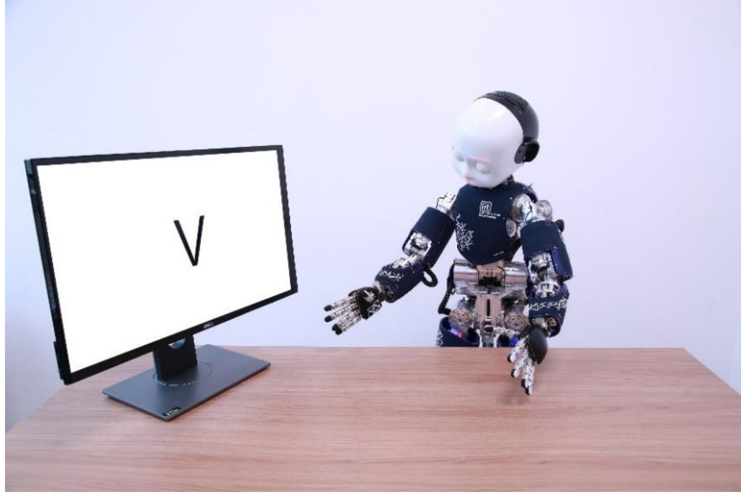

iCub is turning off.

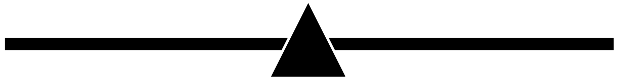

iCub is bored.

M= 31.45; SD: 38.51

Item 6

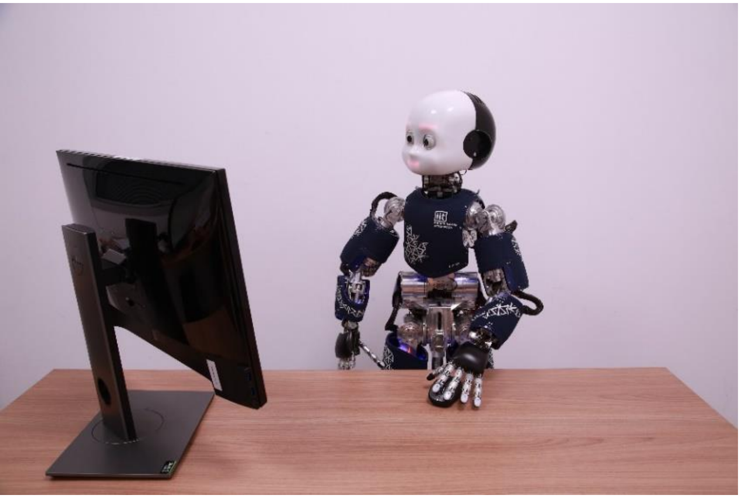

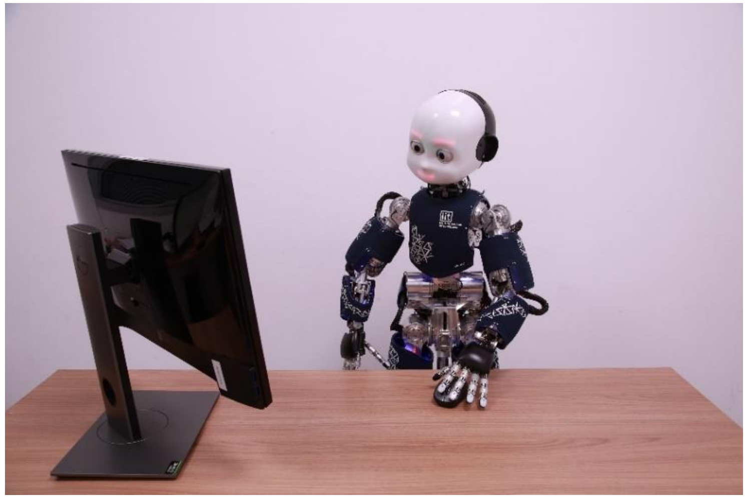

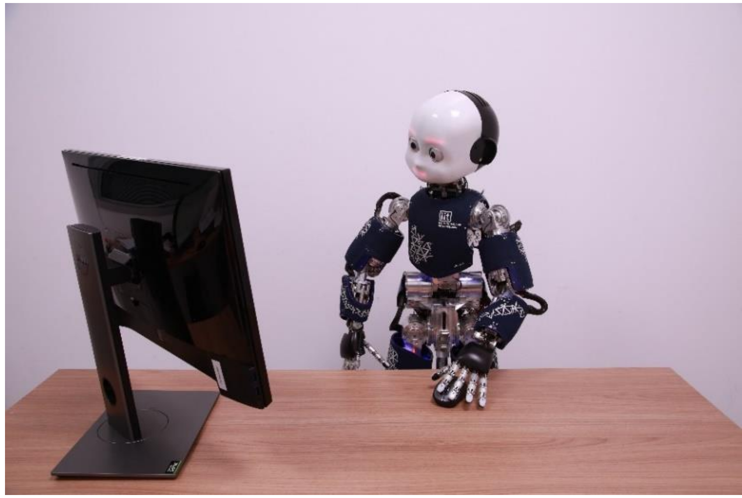

iCub is tracking the mouse cursor.

iCub finds the content on the screen interesting.

M= 43.46; SD: 40.89

Item 7

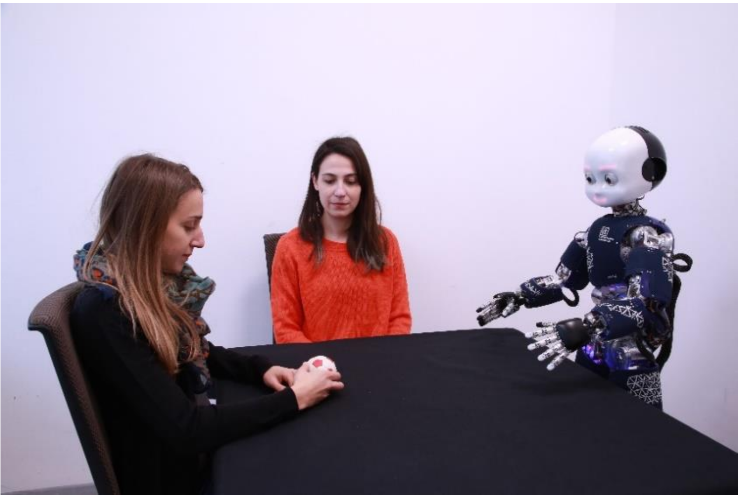

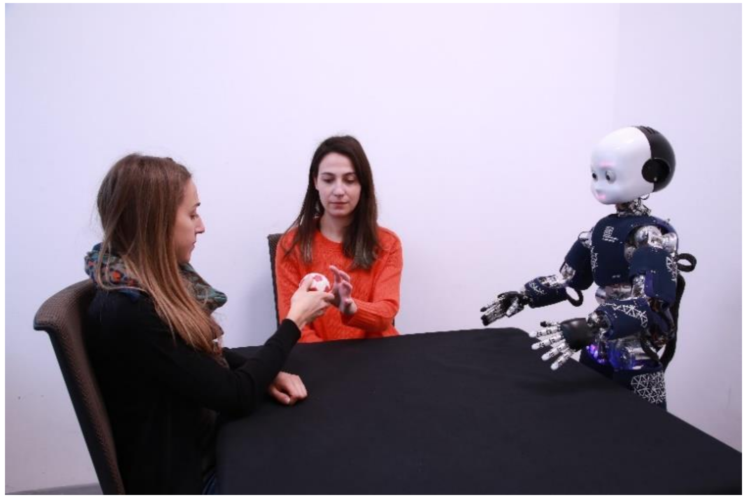

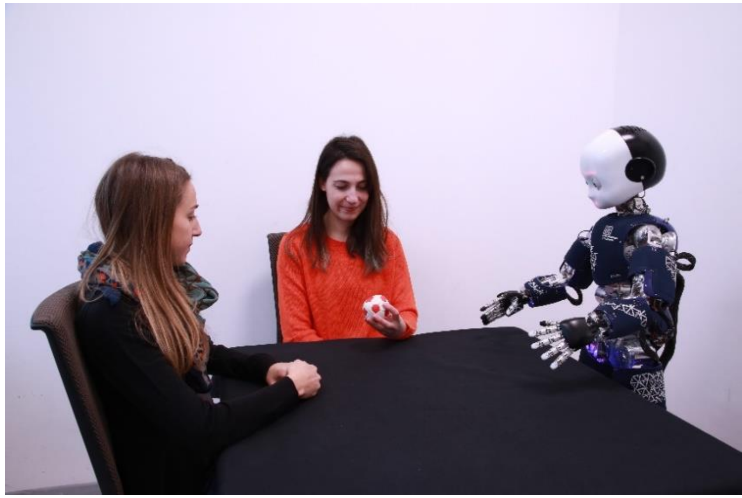

iCub tracks the ball's position.

iCub can't wait to receive the ball.

M= 33.27; SD: 38.11

Item 8

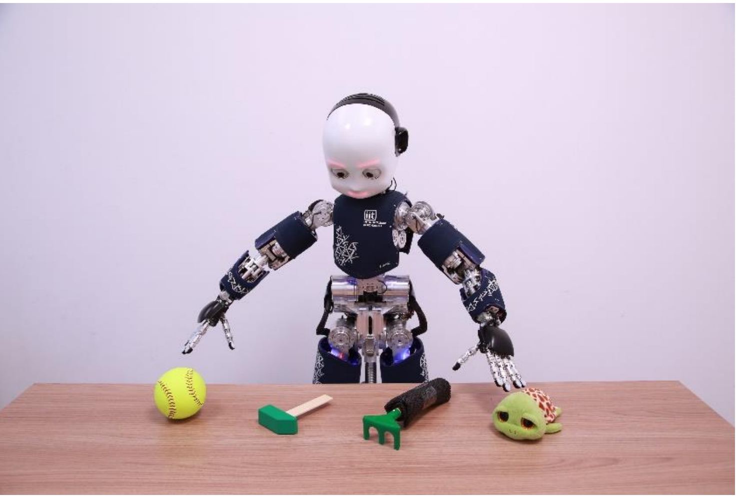

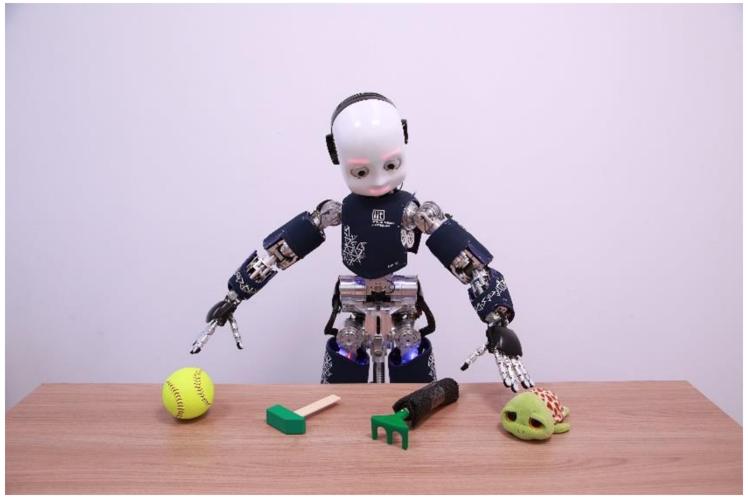

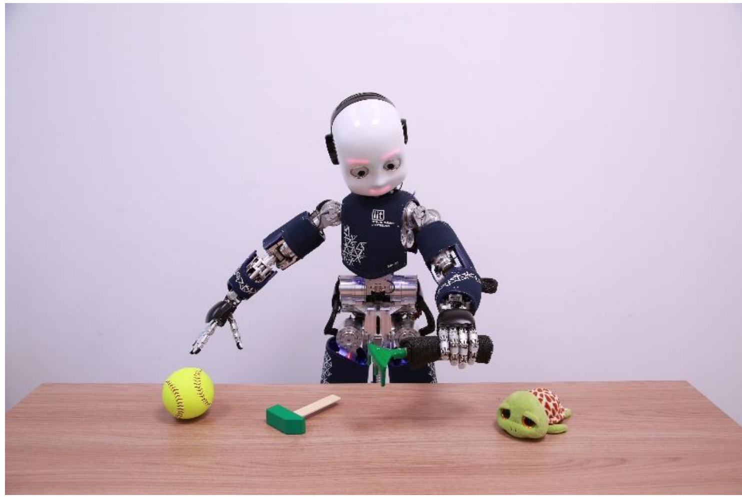

iCub adjusts the force to the weight of the object.

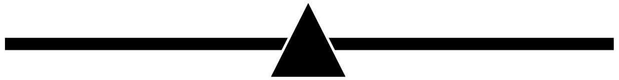

iCub pretends to be a gardener.

M= 22.84; SD: 30.21

Item 9

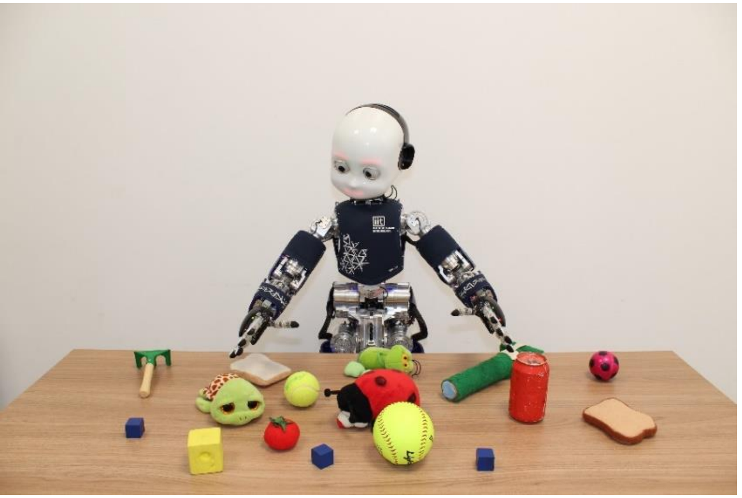

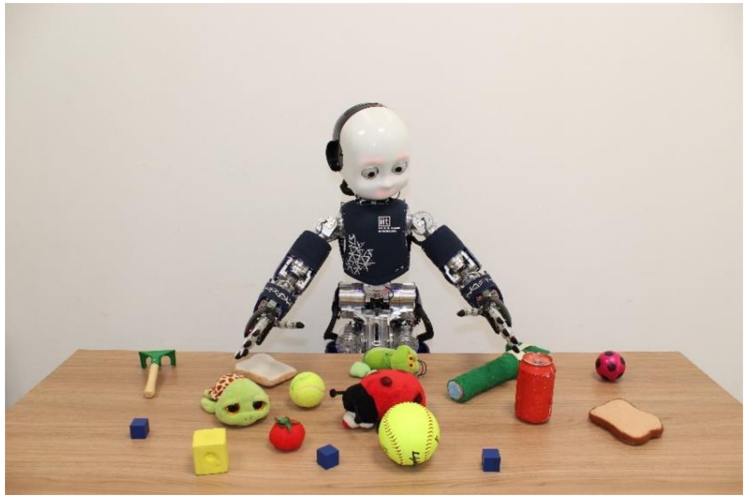

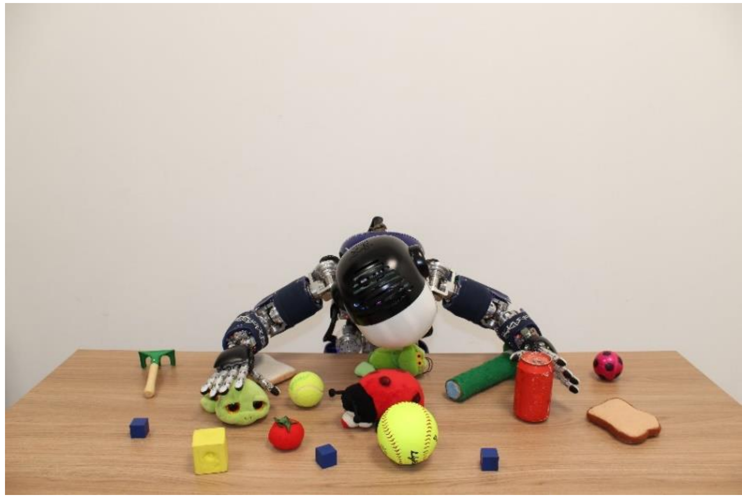

iCub has broken motors.

iCub gave up on finding the favorite toy.

M= 55.72; SD: 41.15

Item 10

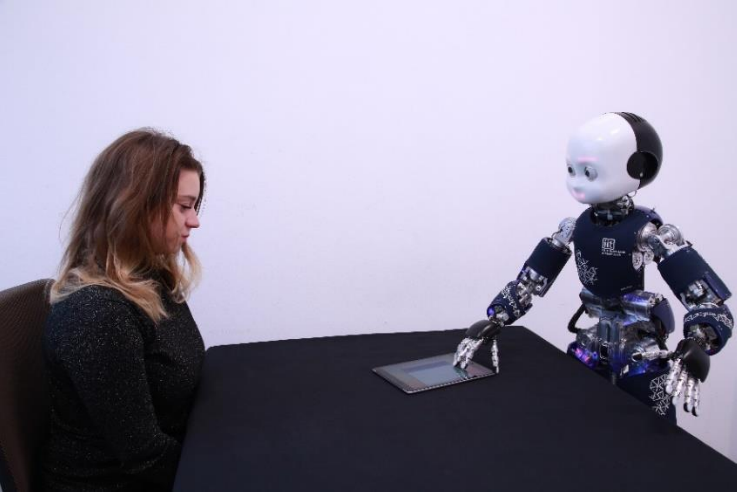

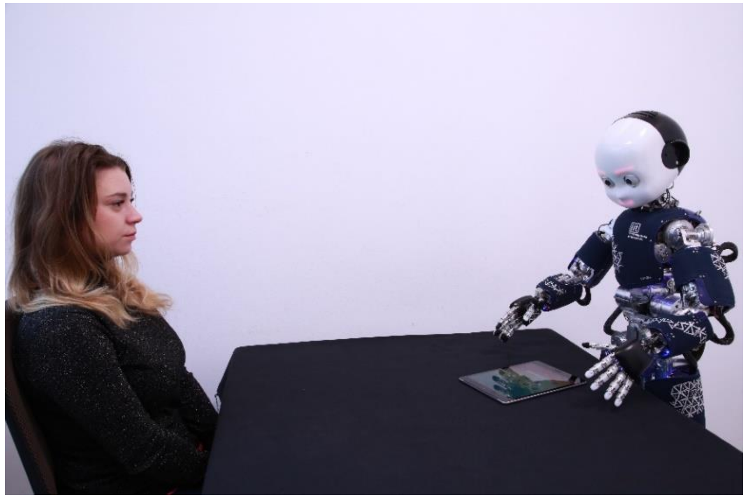

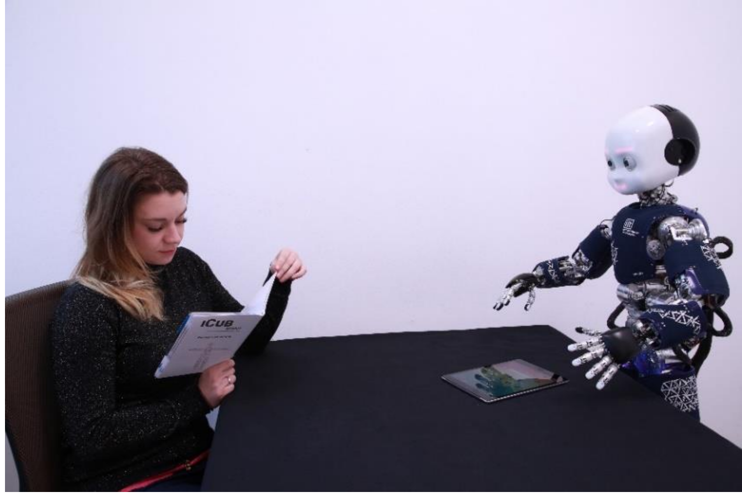

iCub detected differences between the old and the new object in the scene.

iCub wants to read from the book too.

M= 32.35; SD: 37.37

Item 11

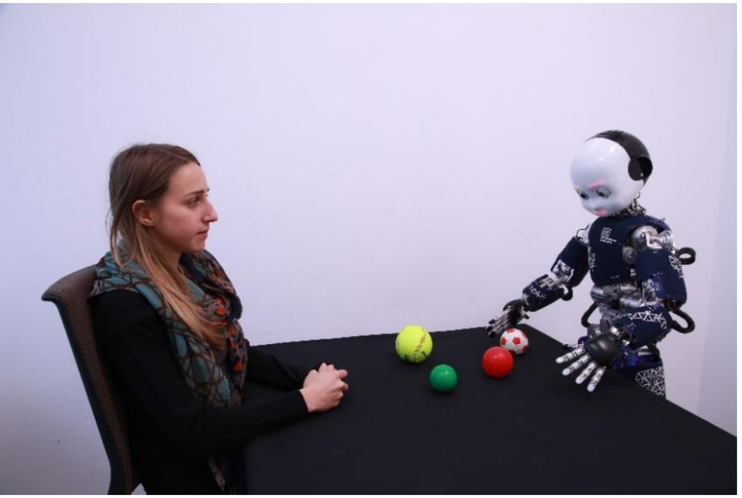

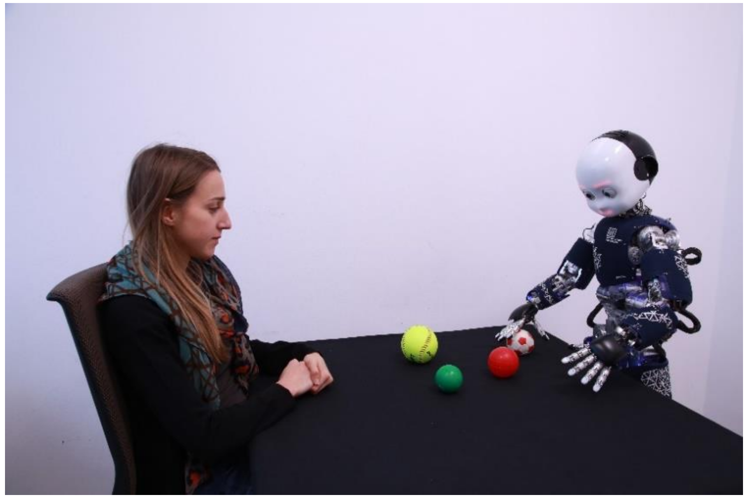

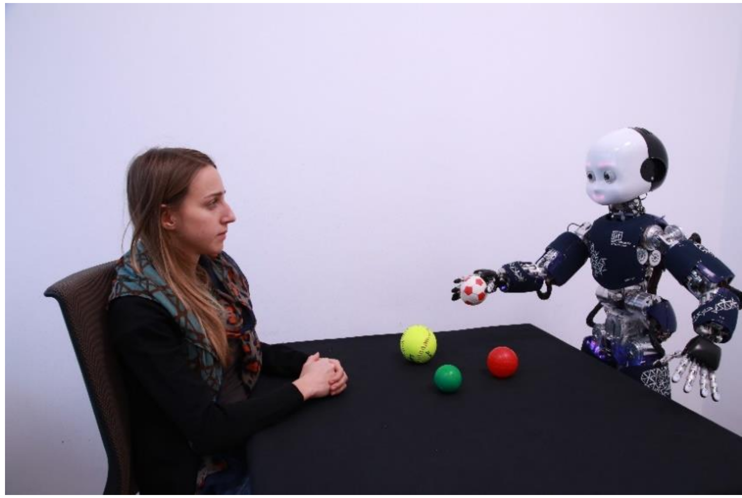

iCub calculates the weight of the balls.

iCub wants to play with the girl.

M= 66.05; SD: 39.47

Item 12

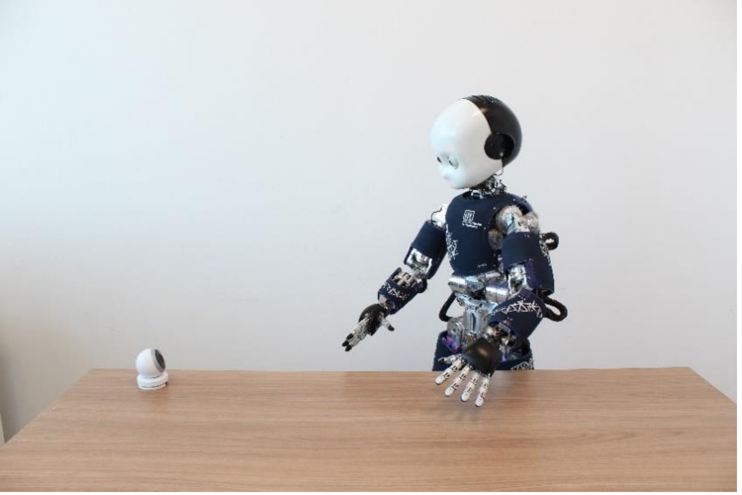
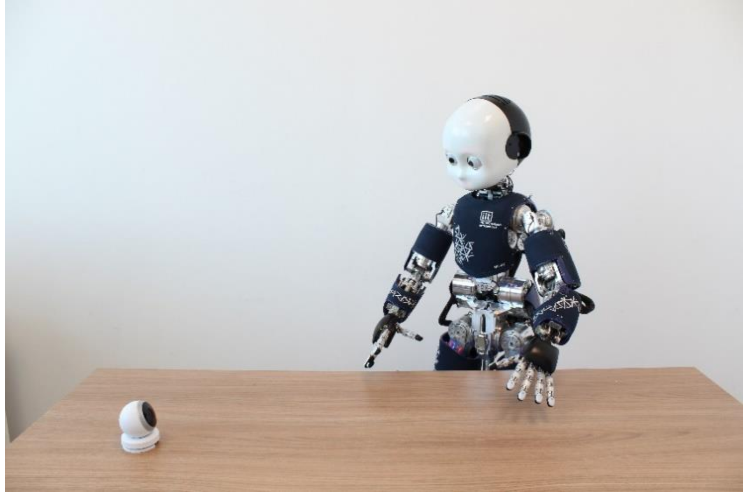
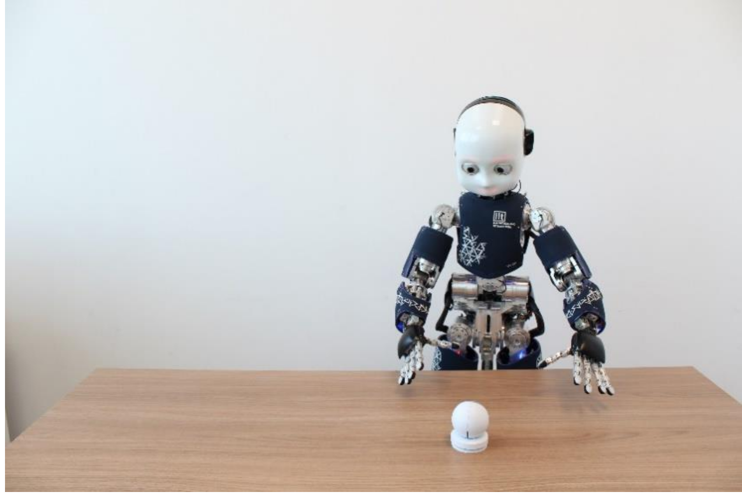

iCub aligns the head with the webcam.

iCub is surprised by the webcam moving.

M= 28.56; SD: 35.14

Item 13

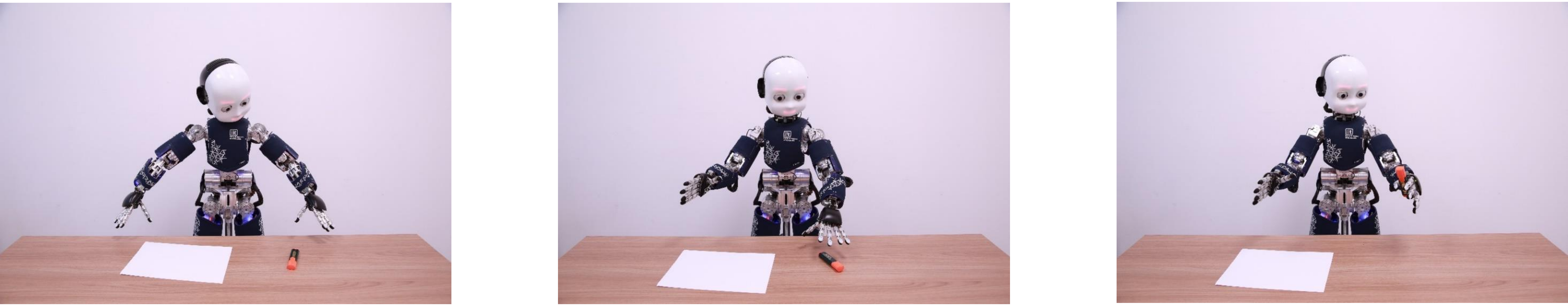

iCub optimizes grip for small objects.

iCub wants to draw something.

M= 42.65; SD: 40.26

Item 14

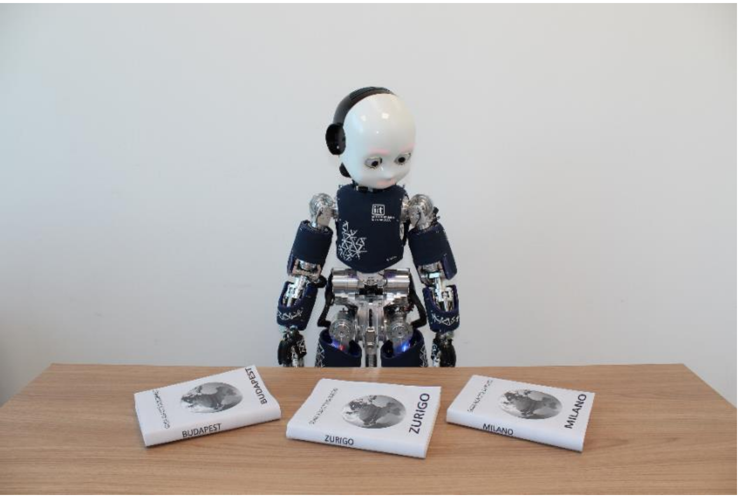

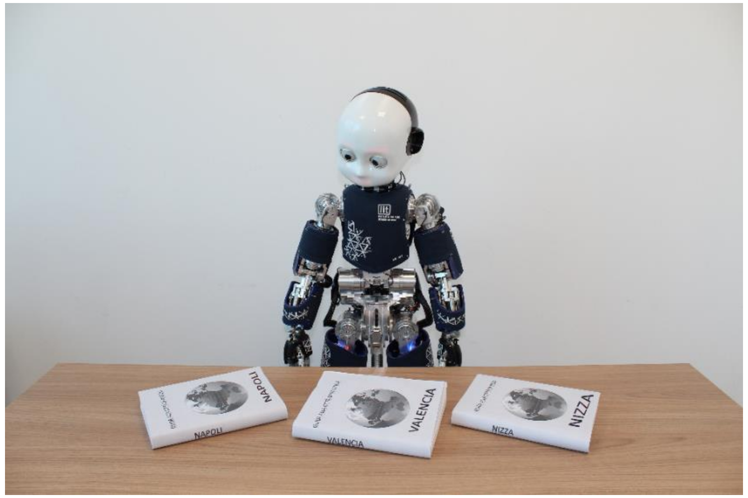

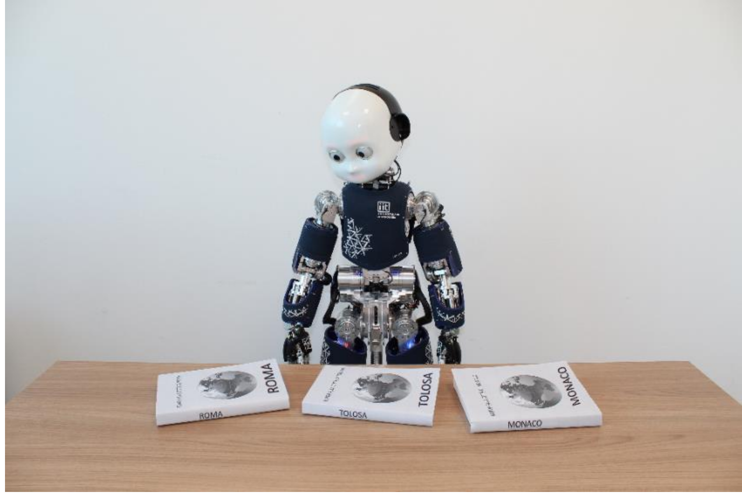

iCub orders Italian cities alphabetically.

iCub prefers Italian cities to other cities.

M= 41.85; SD: 40.47

Item 15

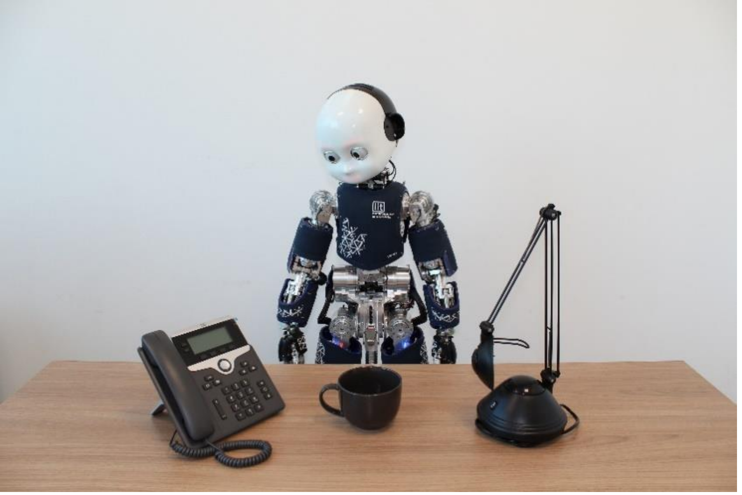

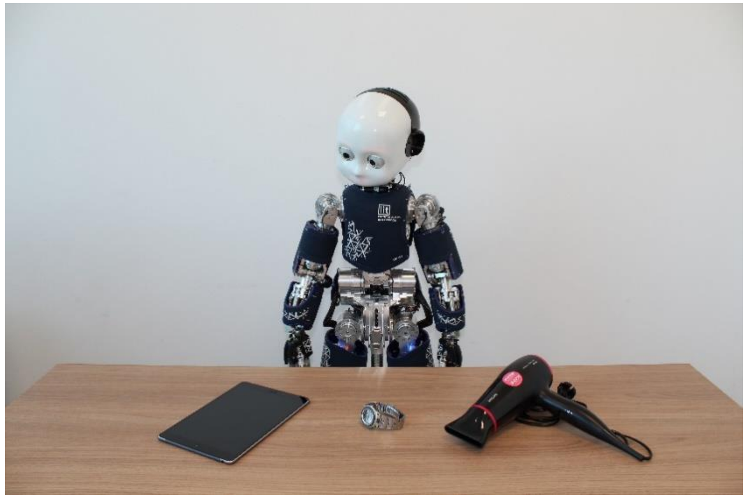

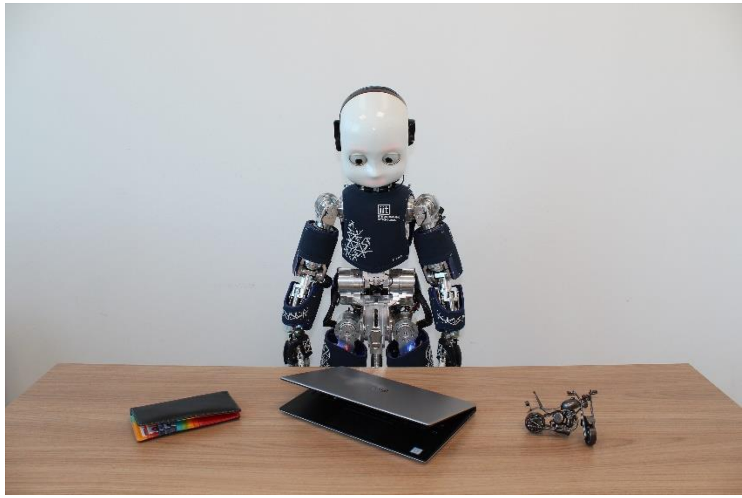

iCub easily detects objects with screens.

iCub finds digital technology intriguing.

M= 33.79; SD: 38.01

Item 16

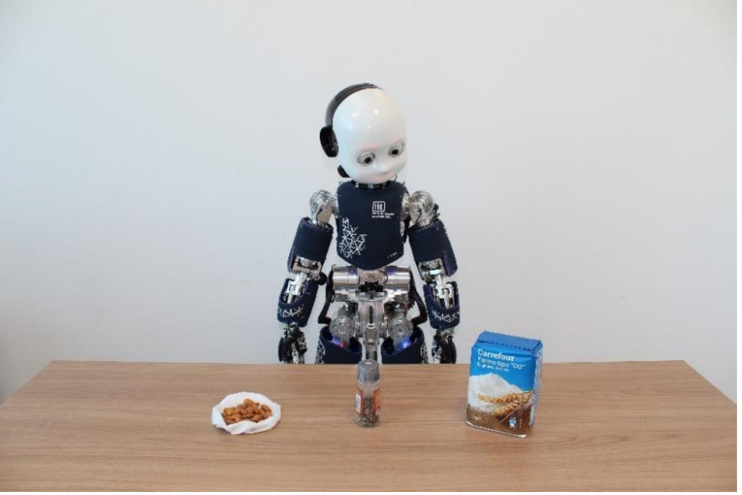

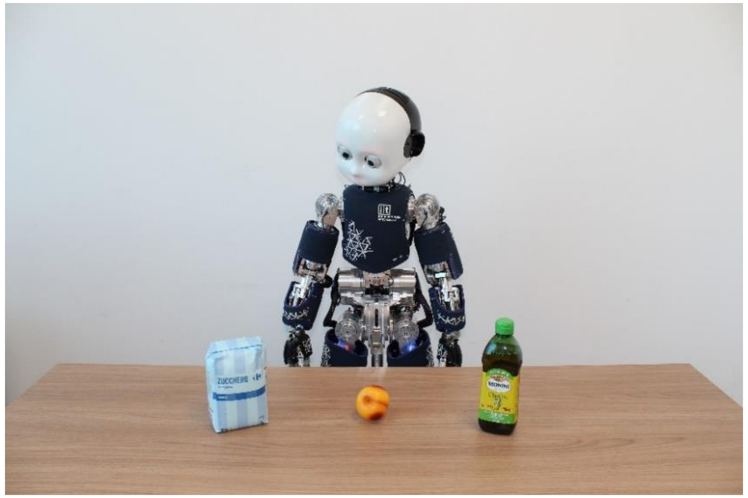

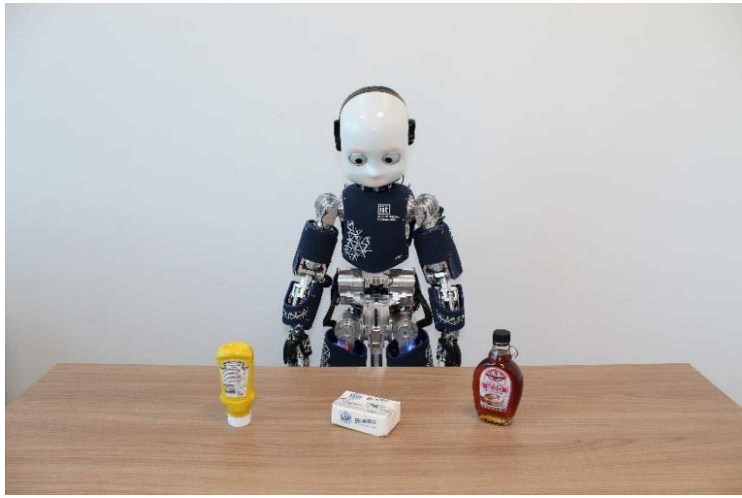

iCub follows the recipe's instructions.

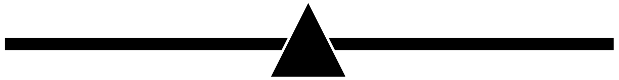

iCub has decided to bake a cake.

M= 33.28; SD: 36.97

Item 17

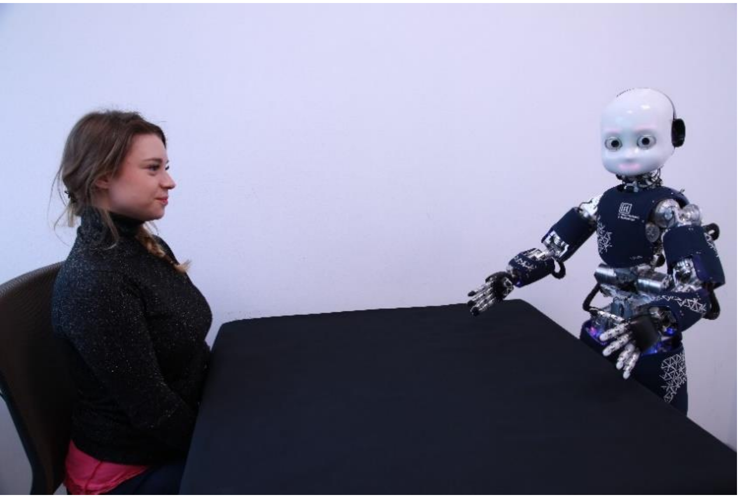

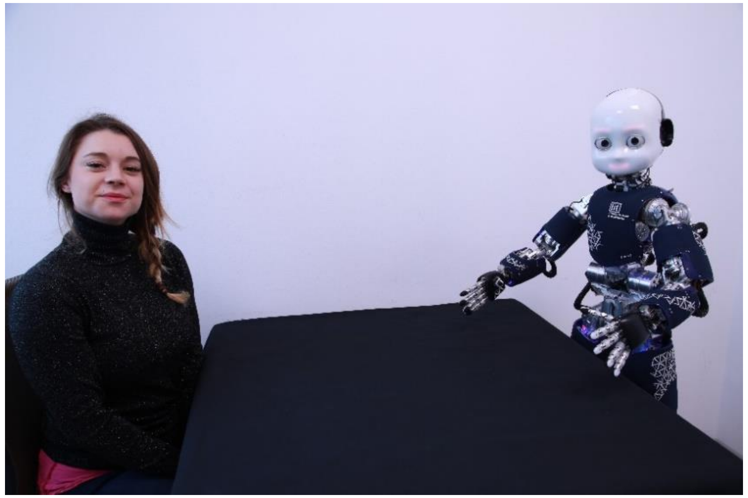

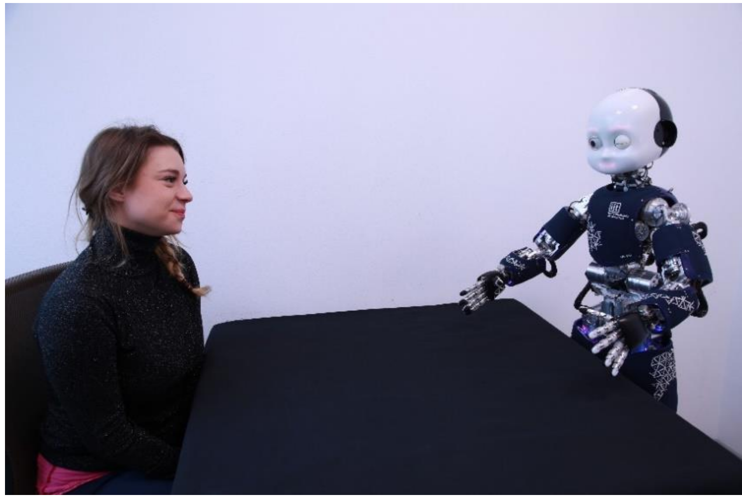

iCub has the eyelid broken.

iCub tries to be funny.

M= 65.04; SD: 38.54

Item 18

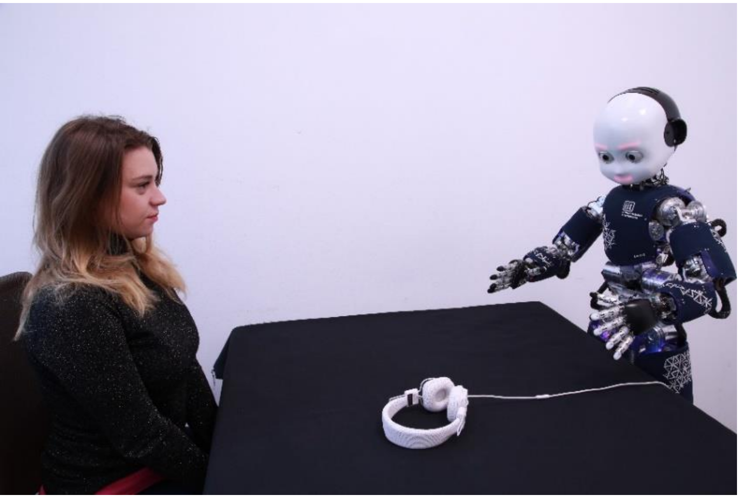
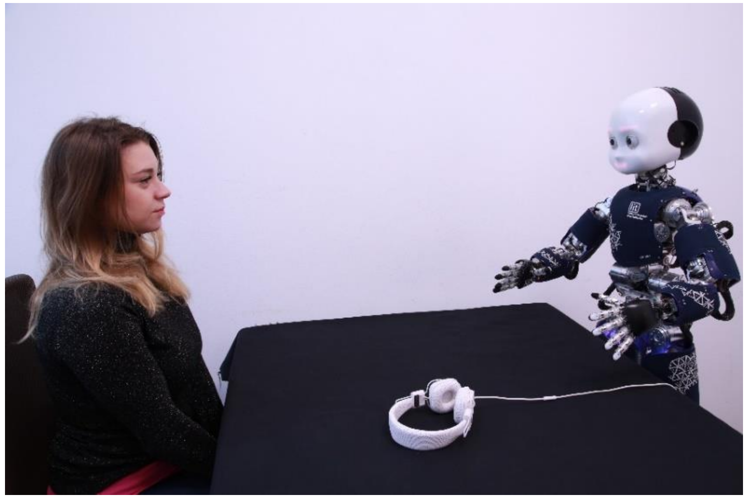
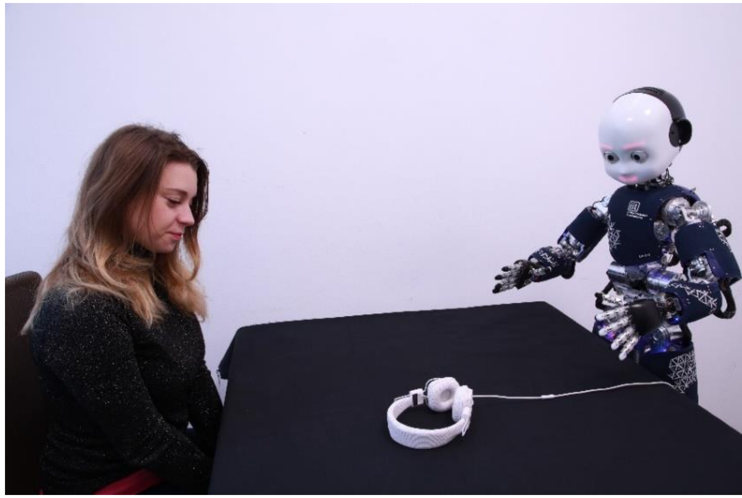

iCub measures the distance between the girl and the headphones.

iCub expects that the girl would lend the headphones.

M= 38.09; SD: 38.33

Item 19

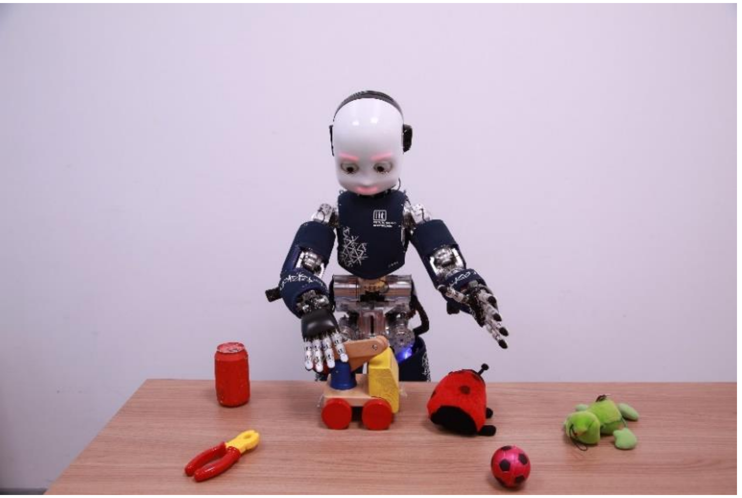

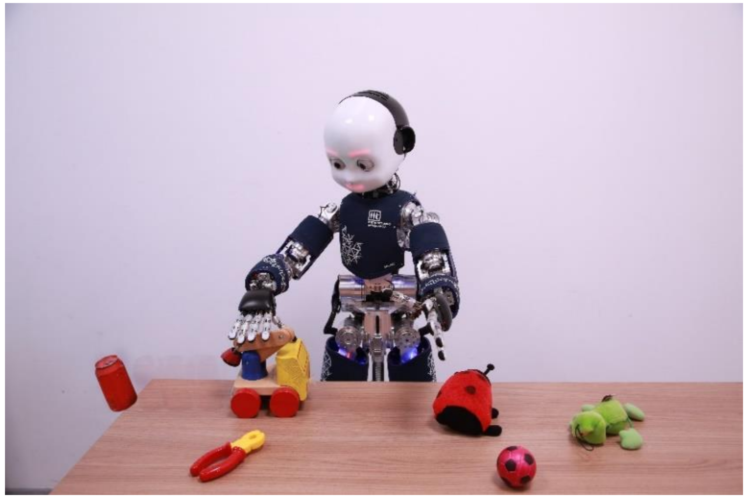

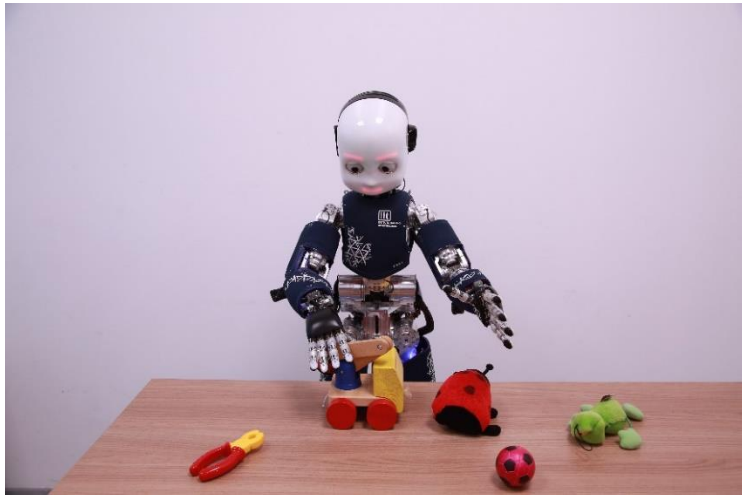

iCub cannot pause the procedure despite the falling can.

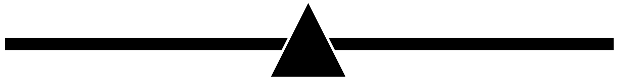

iCub does not intend to pick up the fallen can

M= 24.71; SD: 31.91

Item 20

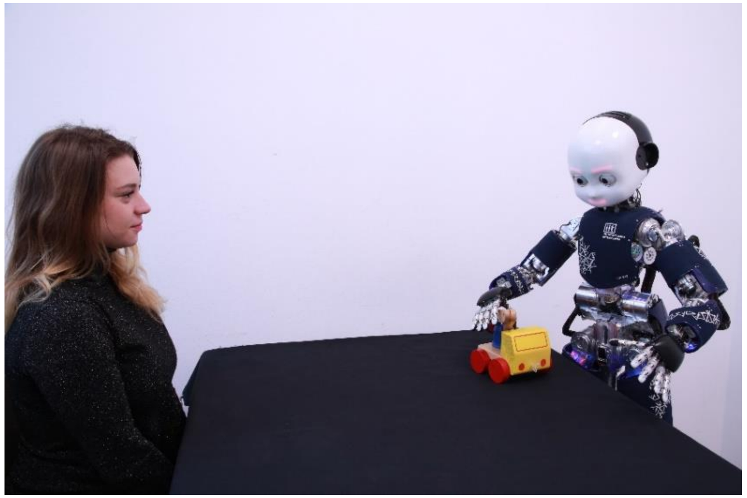

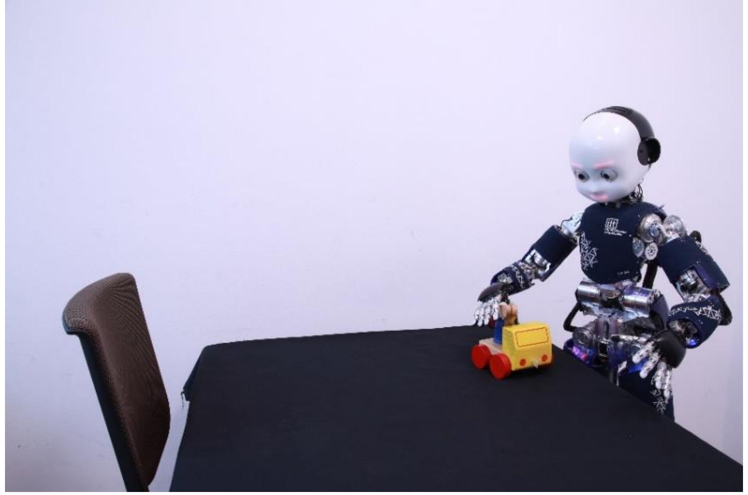

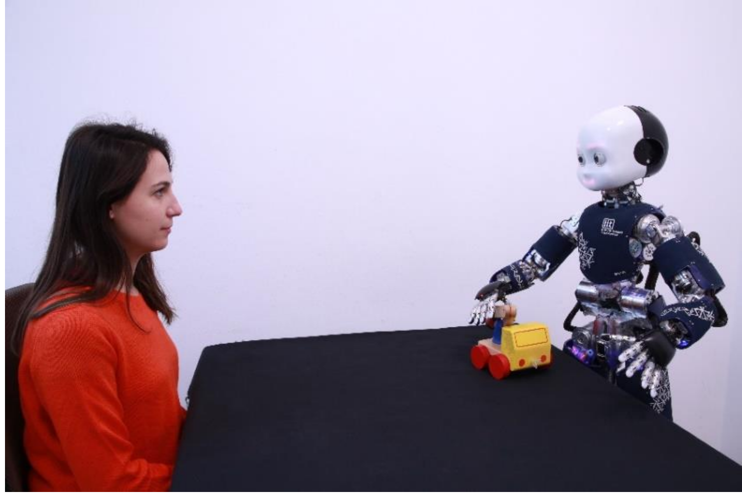

iCub turns the head to bright colors of the sweater.

iCub realizes that there is a new person.

M= 56.58; SD: 39.13

Item 21

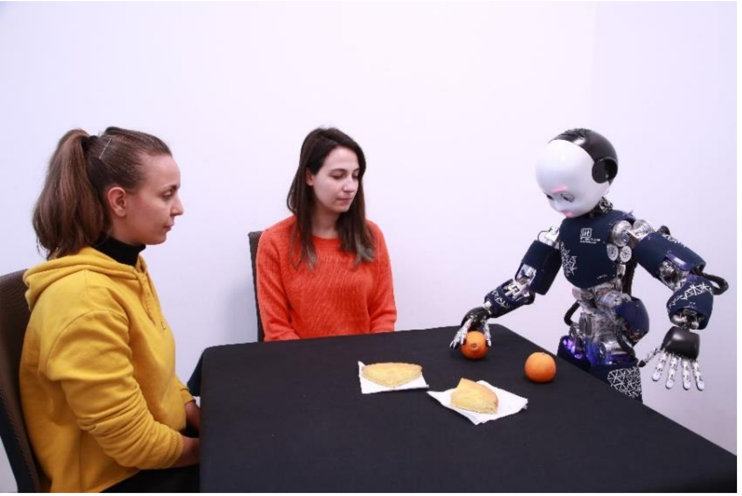

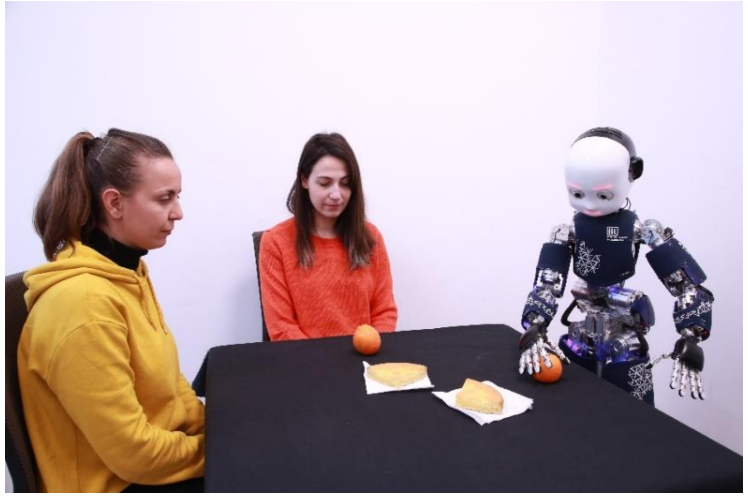

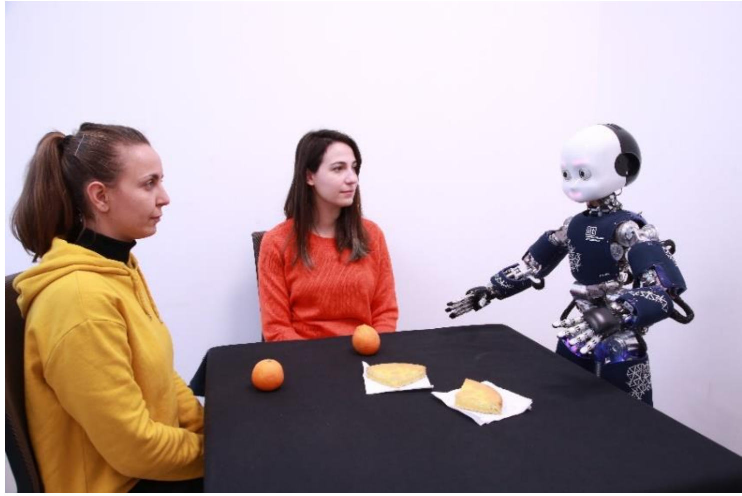

iCub can grasp oranges easier than cakes.

iCub thinks that the girls need some healthy food.

M= 24.42; SD: 33.80

Item 22

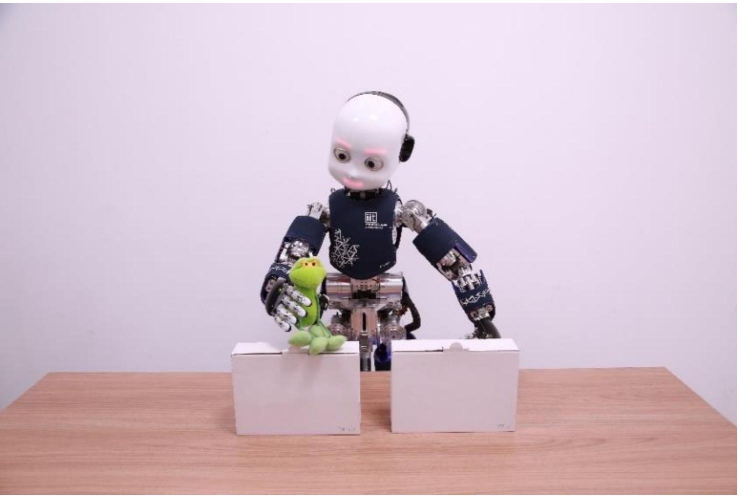

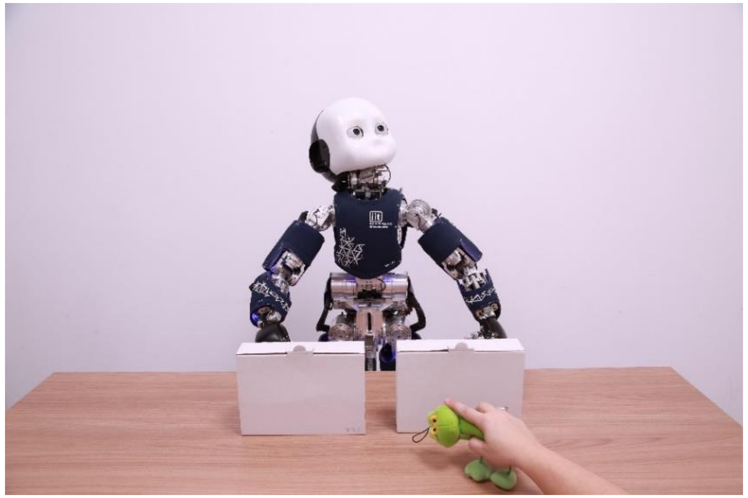

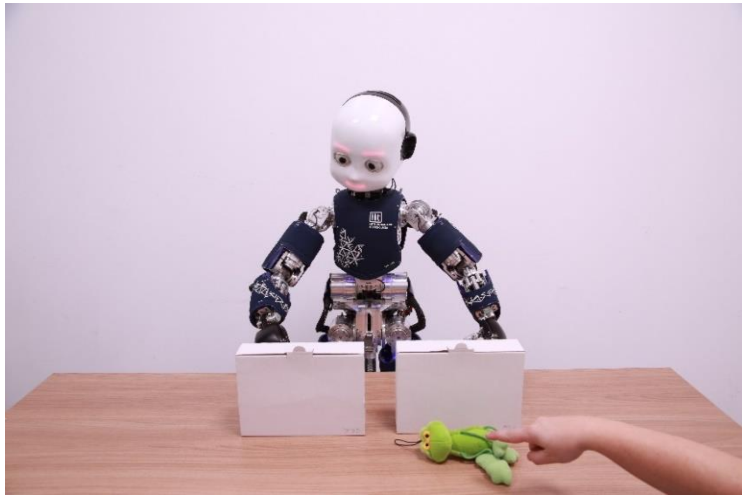

iCub is turning the head to the initial position.

iCub is not interested in the toy anymore.

M= 34.34; SD: 37.02

Item 23

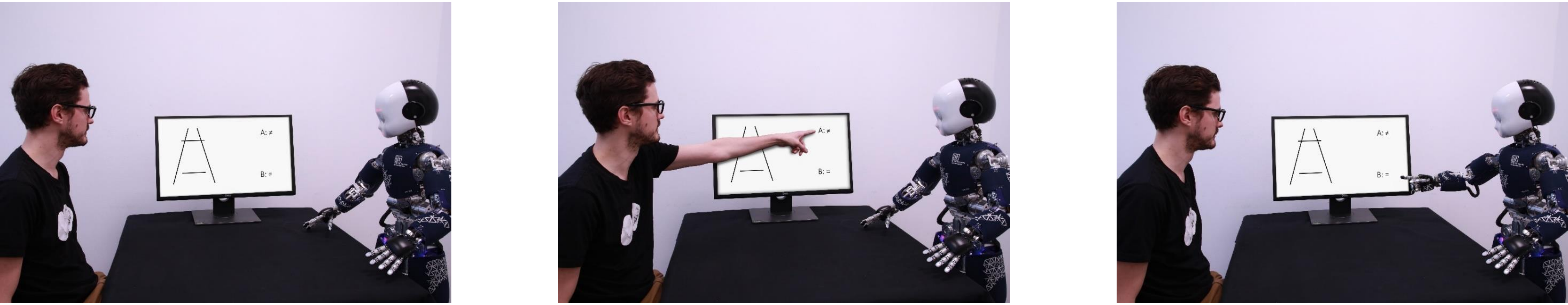

iCub is repeating the pointing movement.

iCub is expressing an opinion.

M= 52.42; SD: 42.11

Item 24

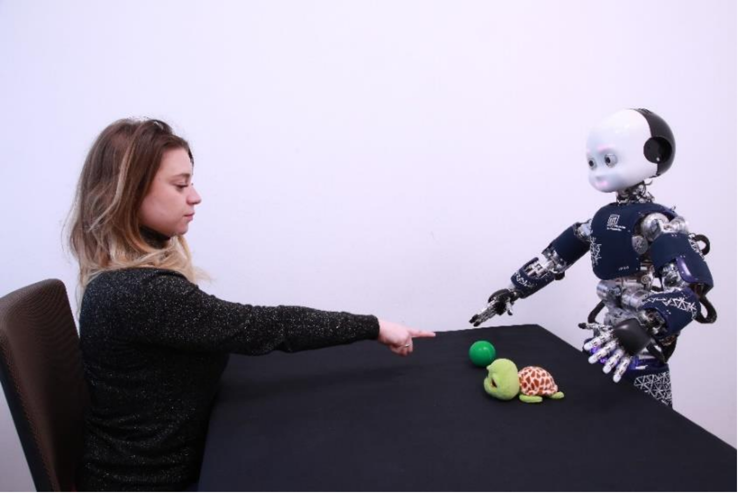

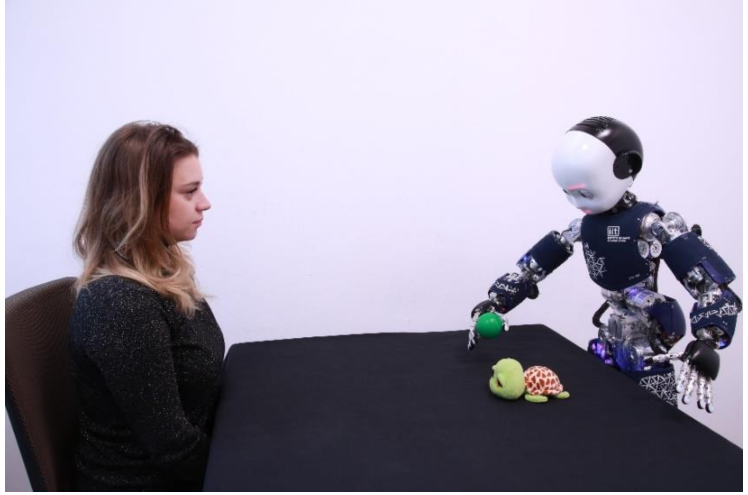

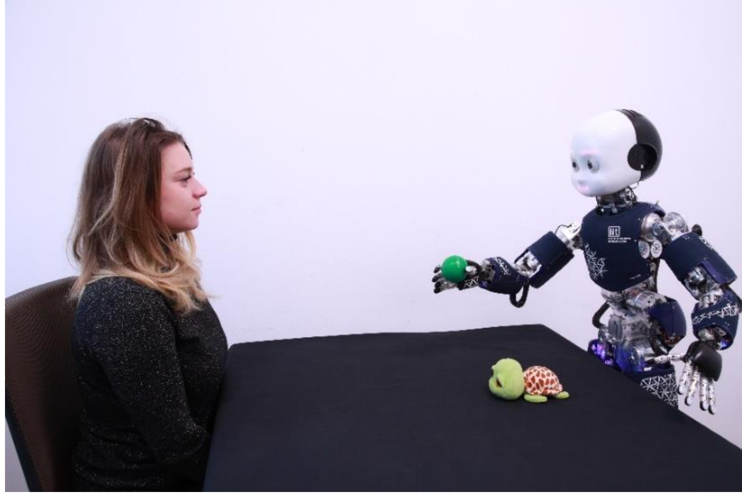

iCub tracked the girl's hand movements.

iCub understood that the girl wants the ball.

M= 68.83; SD: 38.41

Item 25

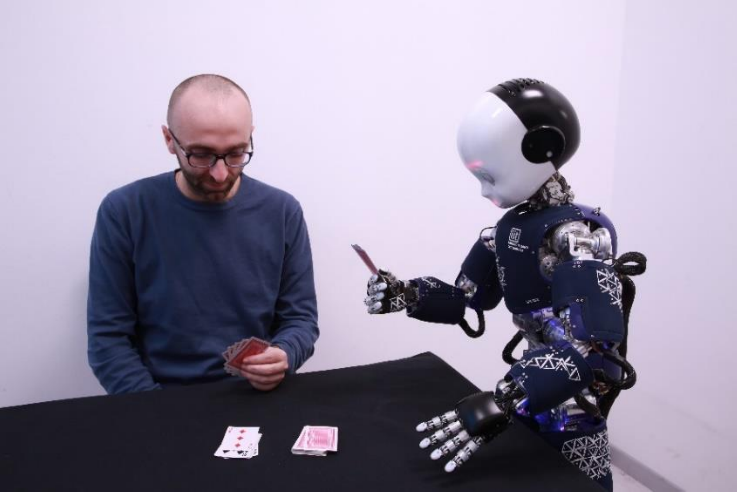

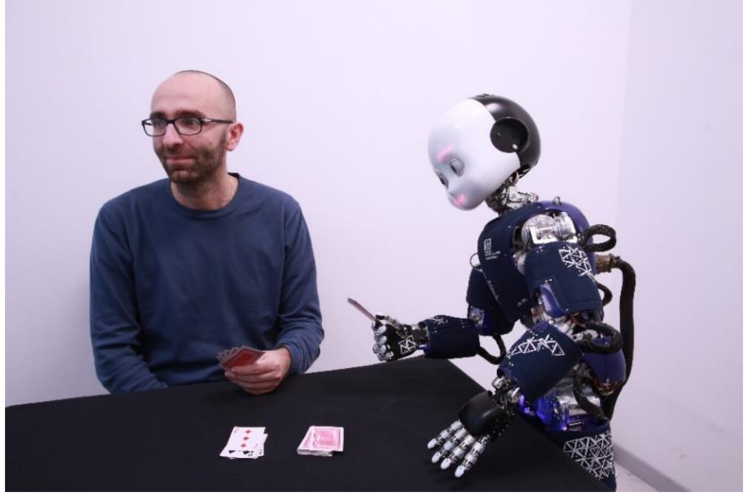

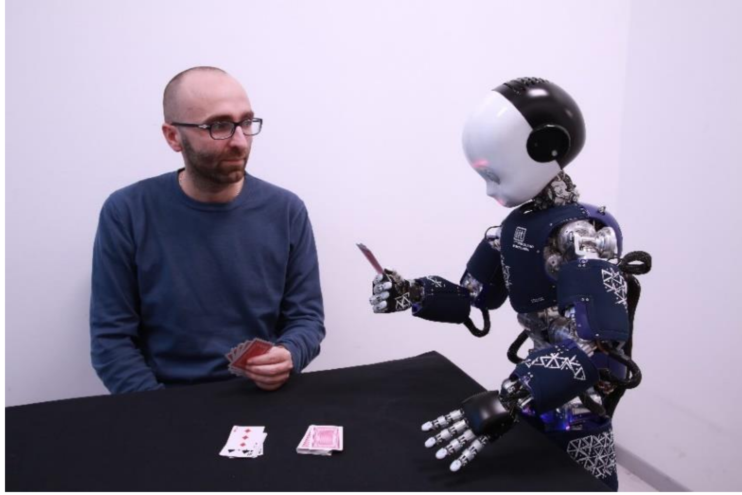

iCub was unbalanced for a moment.

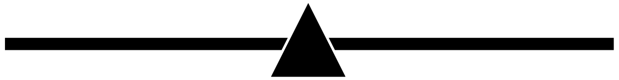

iCub was trying to cheat by looking at opponent's cards.

M= 78.16; SD: 30.59

Item 26

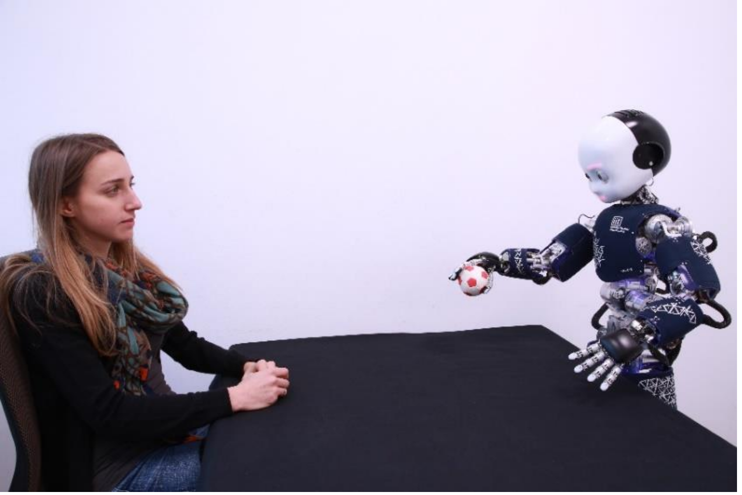

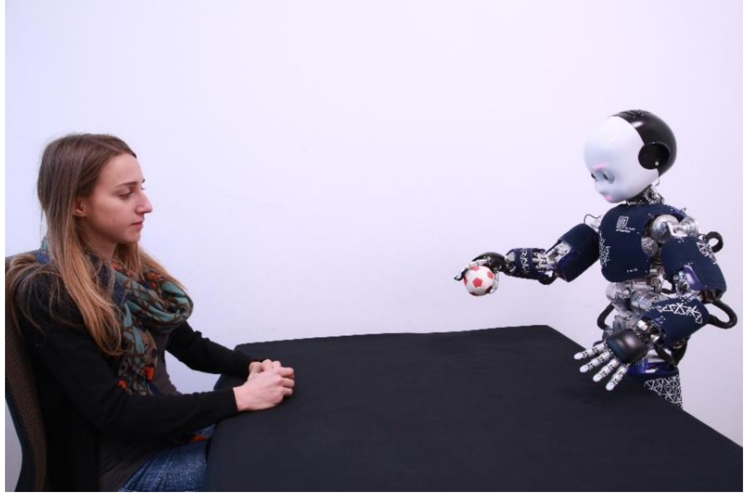

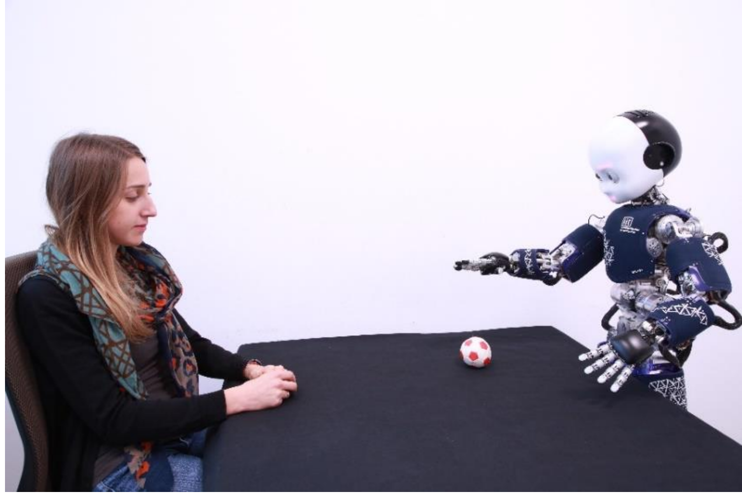

iCub has a failure in the hand.

iCub finds it disappointing that the ball doesn't bounce back.

M= 57.61; SD: 38.94

Item 27

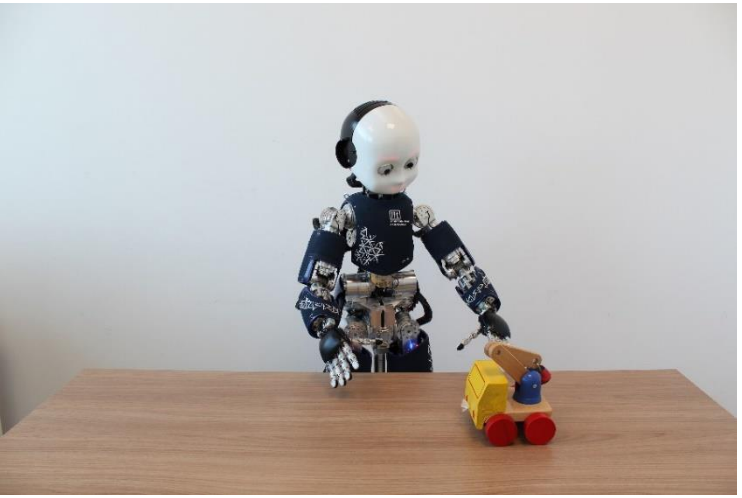
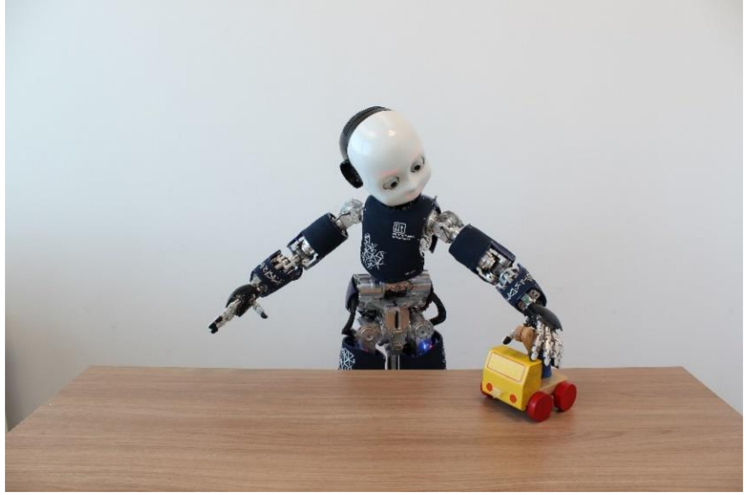
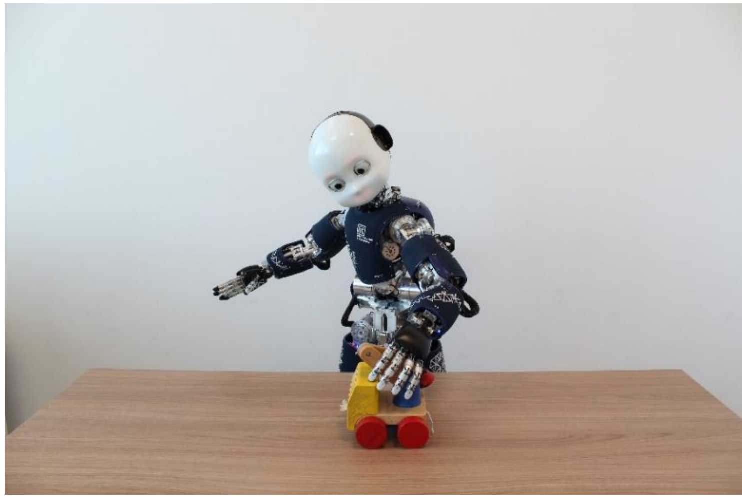

iCub is optimizing head-arm coordination.

iCub is enjoying playing with the truck.

M= 45.08; SD: 40.81

Item 28

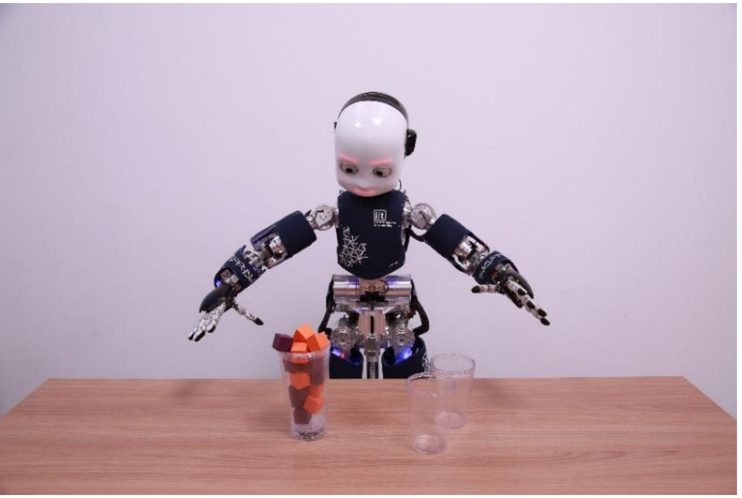

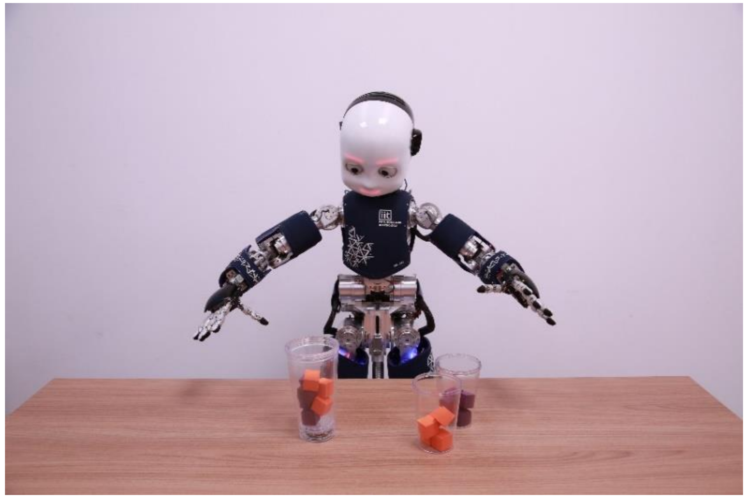

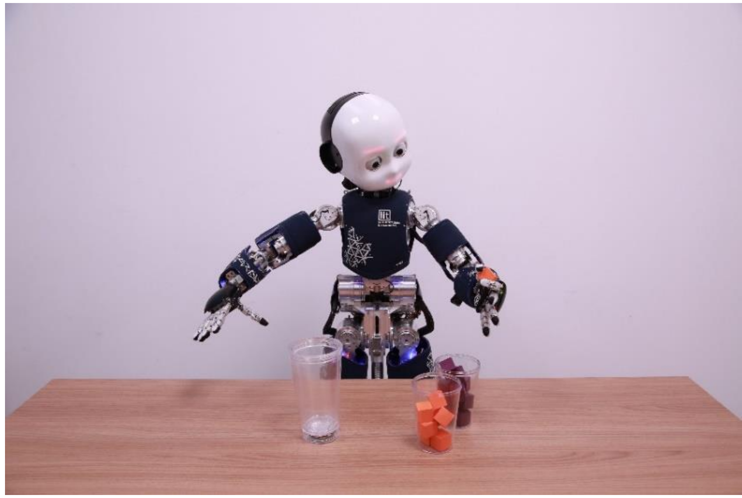

iCub classifies cubes by color.

iCub would like to keep this cube.

M= 10.07; SD: 20.62

Item 29

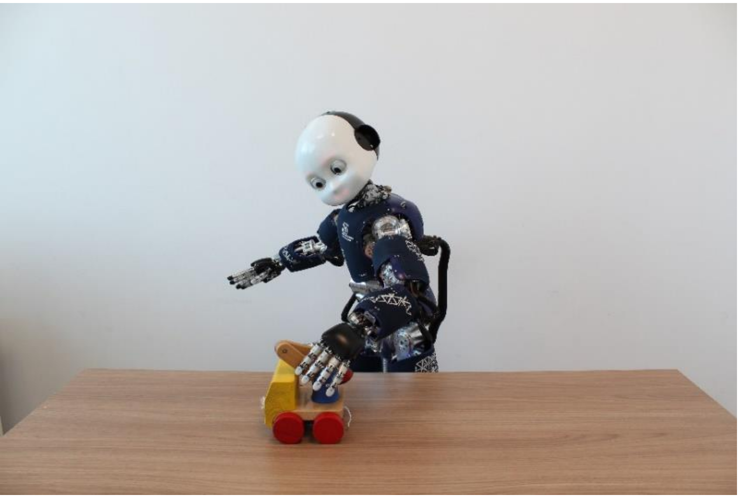

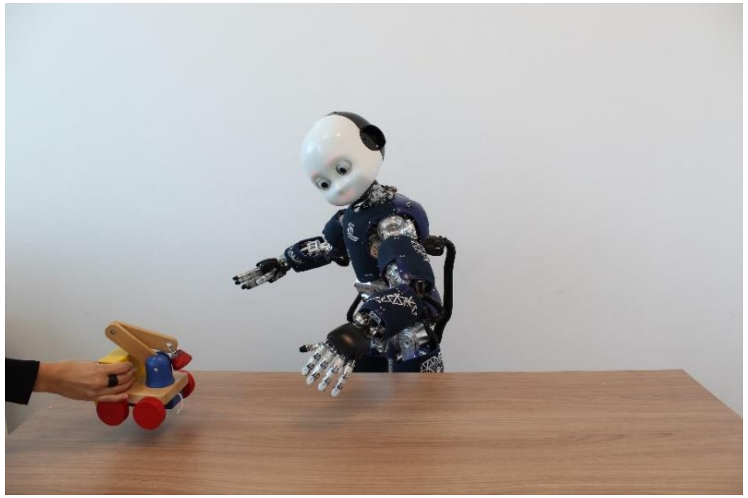

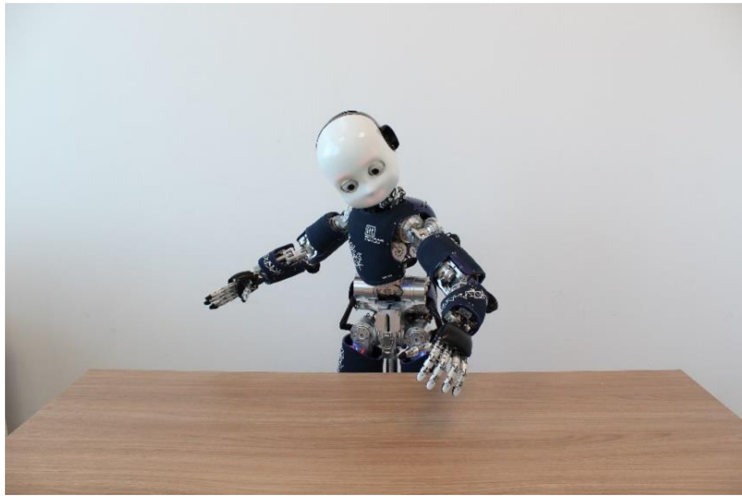

iCub got stuck.

iCub imagines that the toy is still there.

M= 48.55; SD: 41.41

Item 30

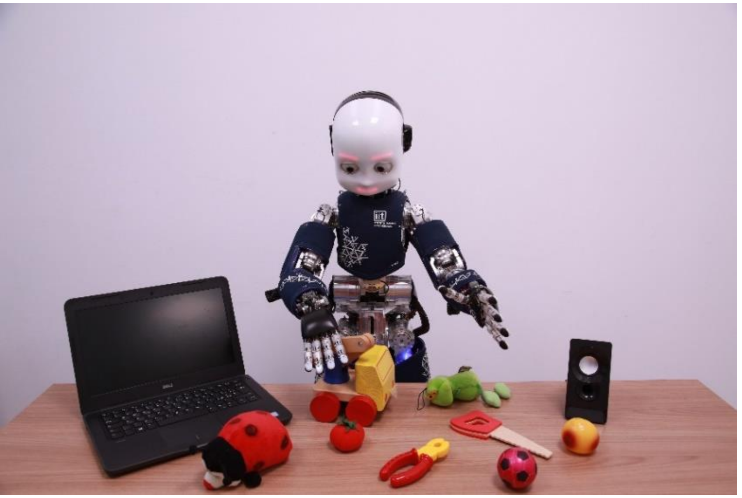

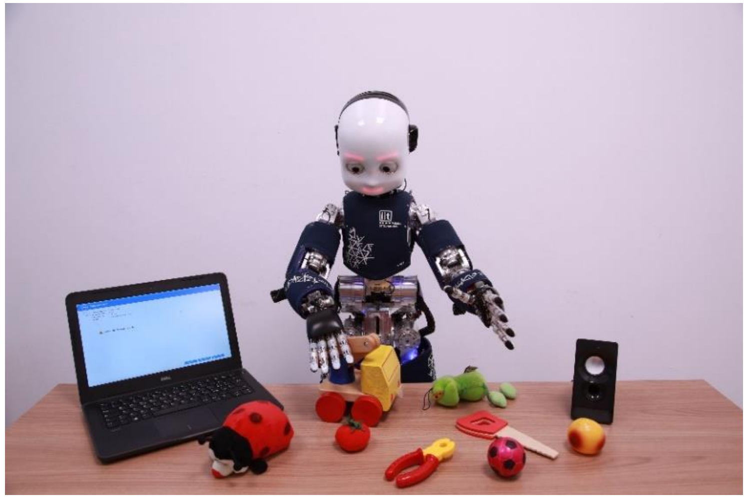

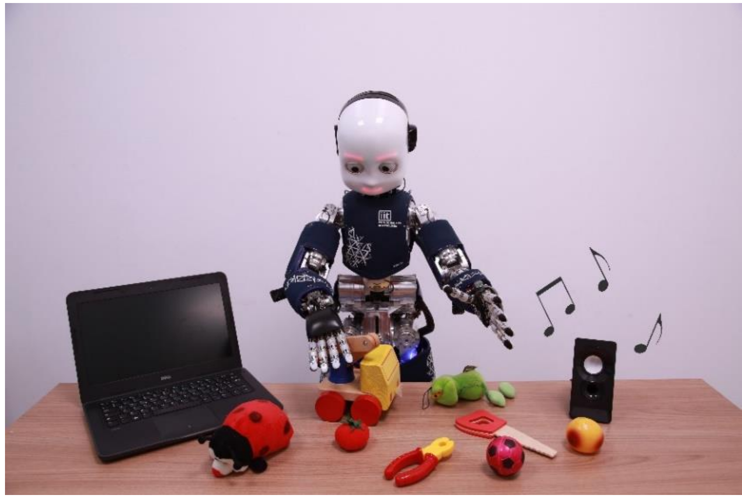

iCub is on stand-by mode.

iCub can't decide which toy to play.

M= 25.10; SD: 34.34

Item 31

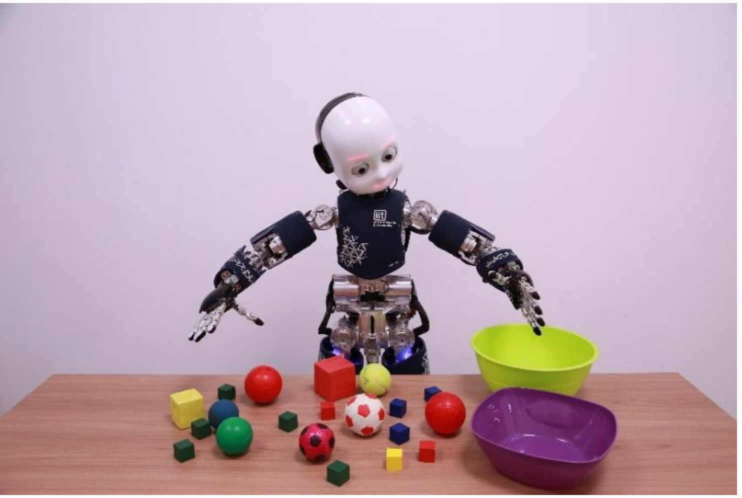

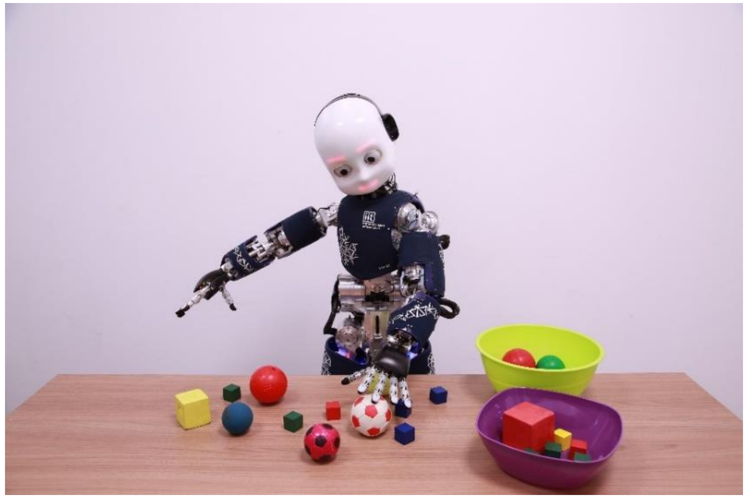

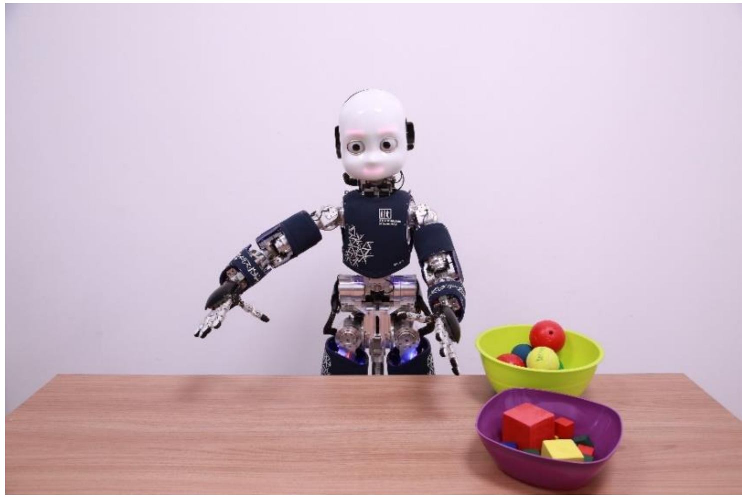

iCub places objects in bowls with corresponding shape.

iCub knows that it's better to clean the table after playing.

M= 11.97; SD: 25.22

Item 32

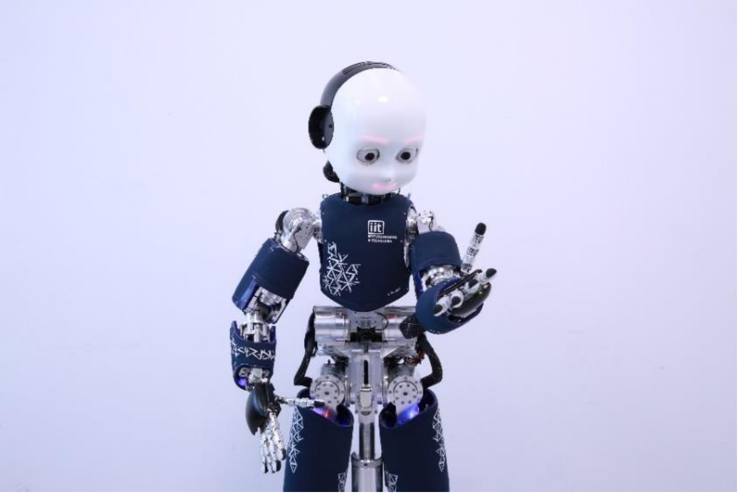

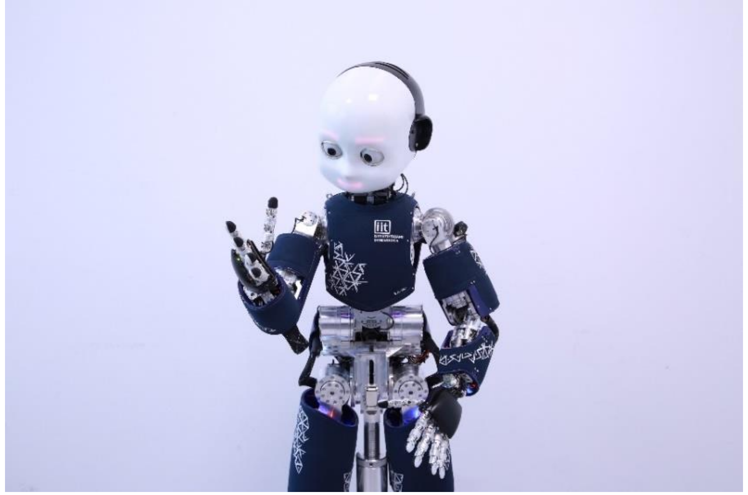

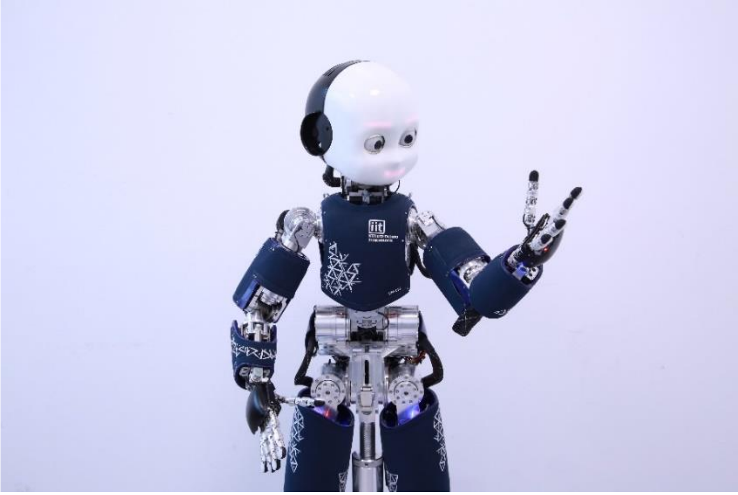

iCub calibrates position of the head with position of the hand.

iCub enjoys counting fingers on each hand.

M= 34.21; SD: 38.33

Item 33

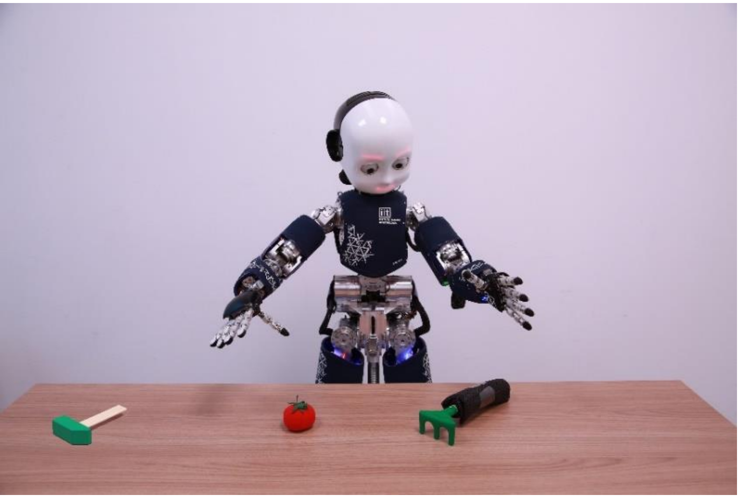

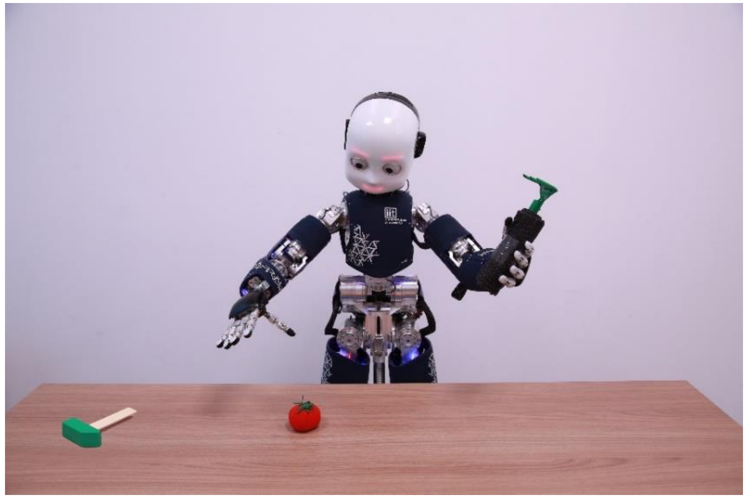

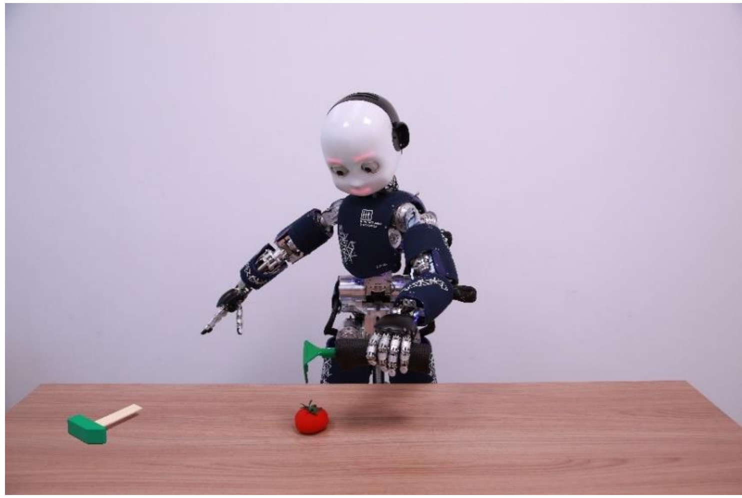

iCub grasped the closest object.

iCub was fascinated by tool use.

M= 46.40; SD: 41.53

Item 34

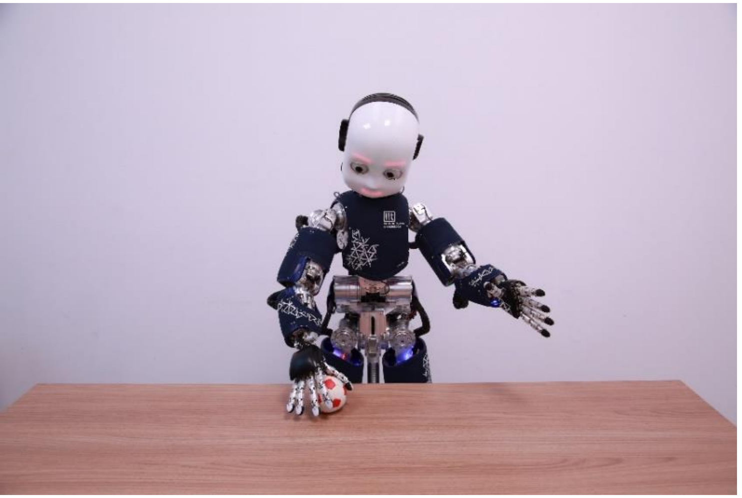
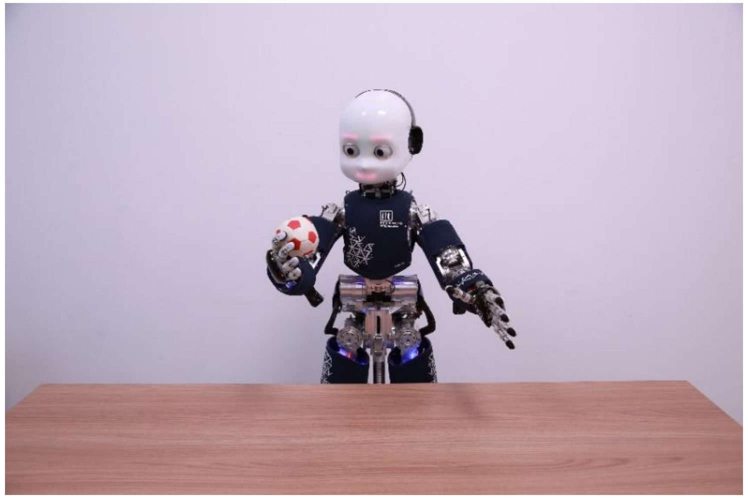
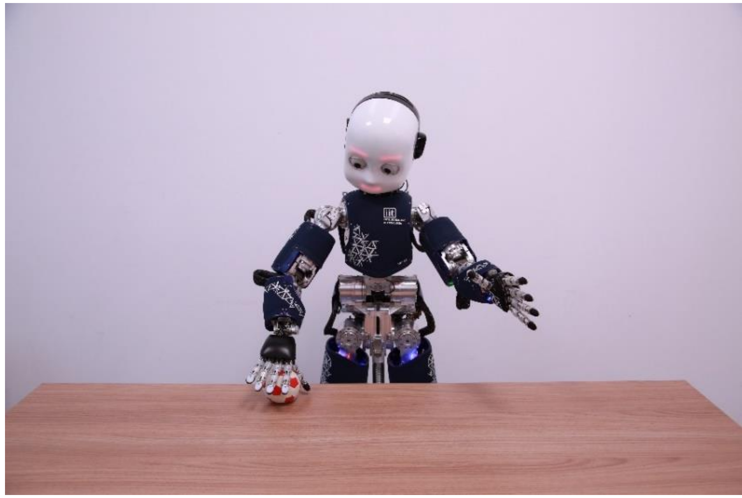

iCub has updated the coordinates of the ball position.

iCub has decided not to throw the ball

M= 34.40; SD: 36.91

The InStance Questionnaire
Supplementary material 2 ITALIAN

Item 0 (Esempio)

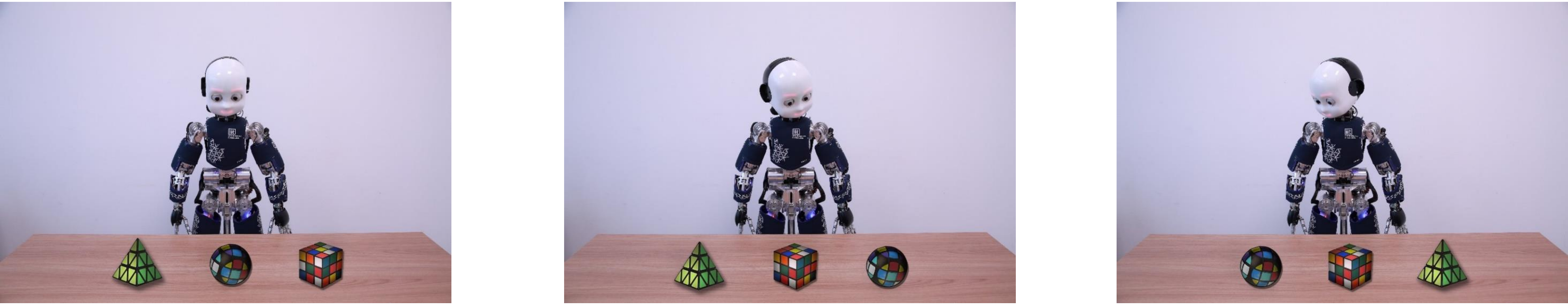

iCub categorizza gli oggetti in base alla loro forma.

iCub preferisce gli oggetti sferici.

M= 55.08; SD: 44.39

Item 1

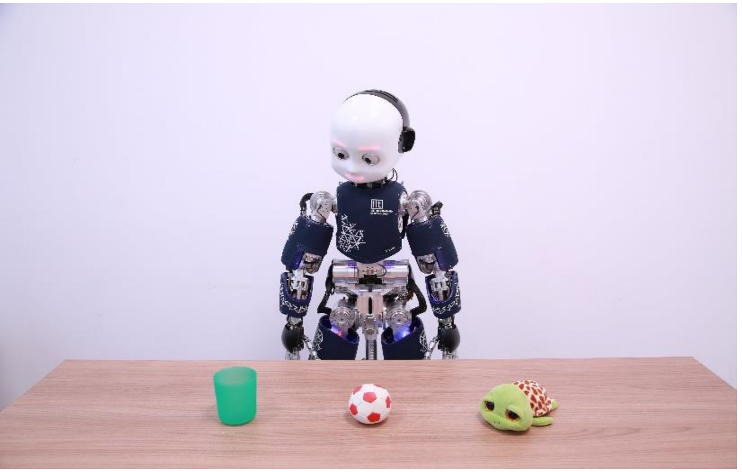

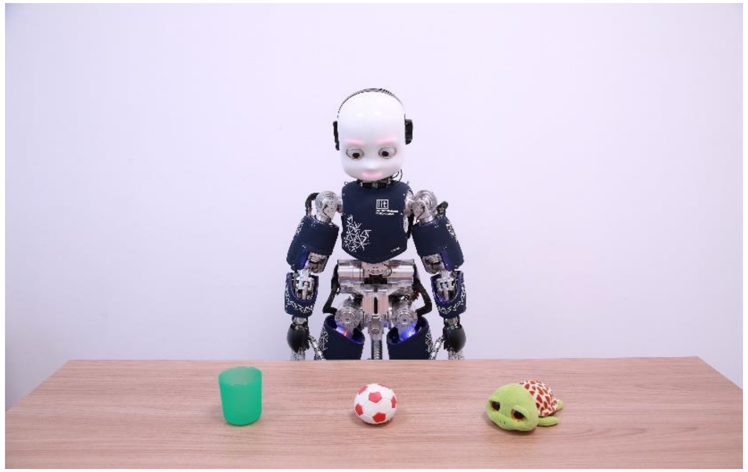

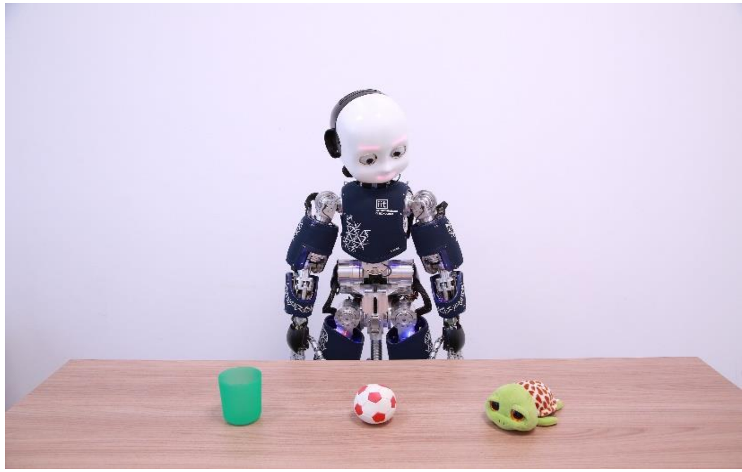

iCub scansiona l’ambiente.

iCub è interessato a questi oggetti.

M= 35.25; SD: 39.72

Item 2

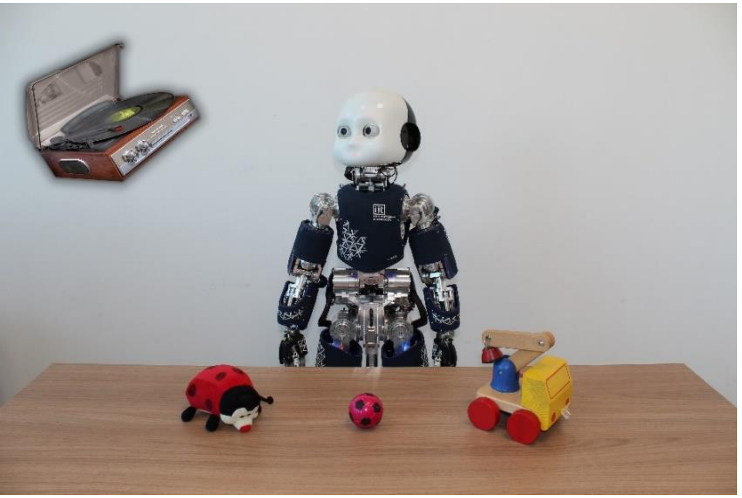

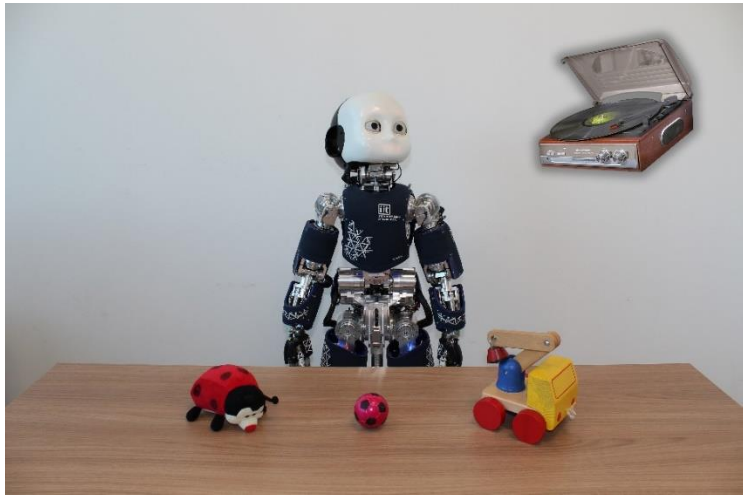

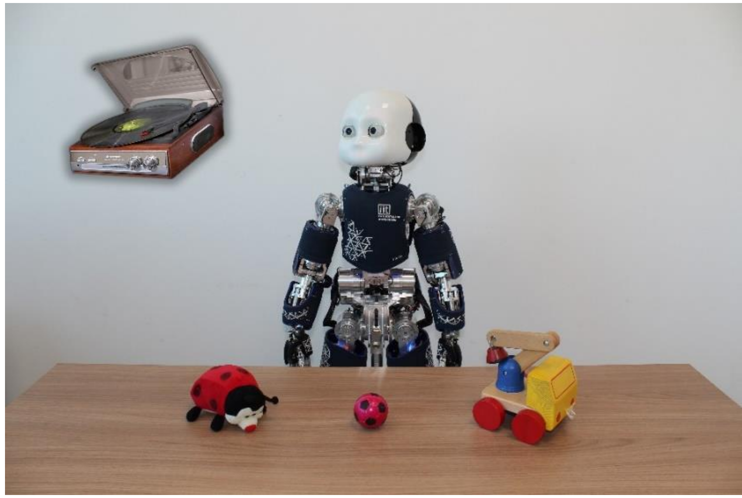

iCub guarda gli oggetti all’altezza degli occhi.

iCub è sorpreso di vedere l’oggetto in aria.

M= 46.37; SD: 41.91

Item 3

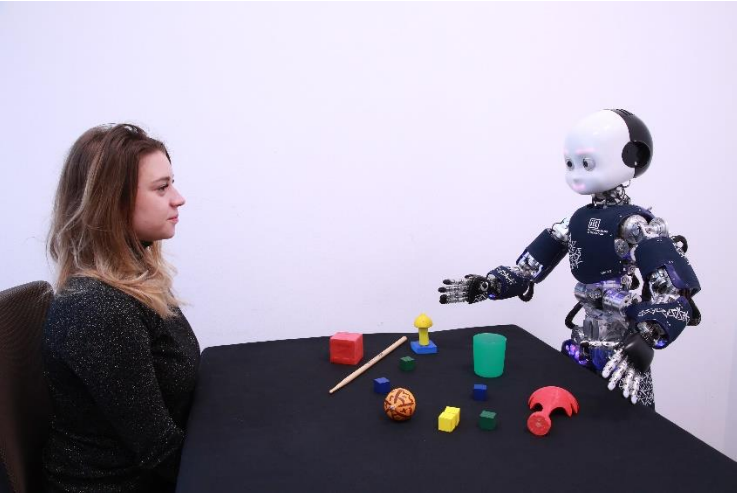

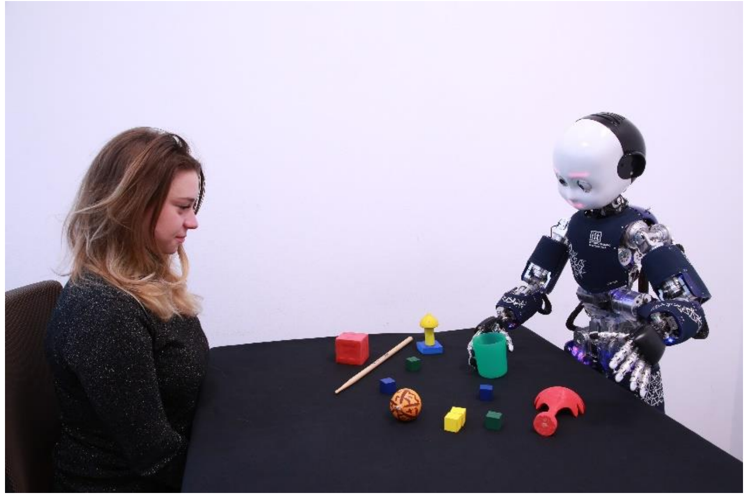

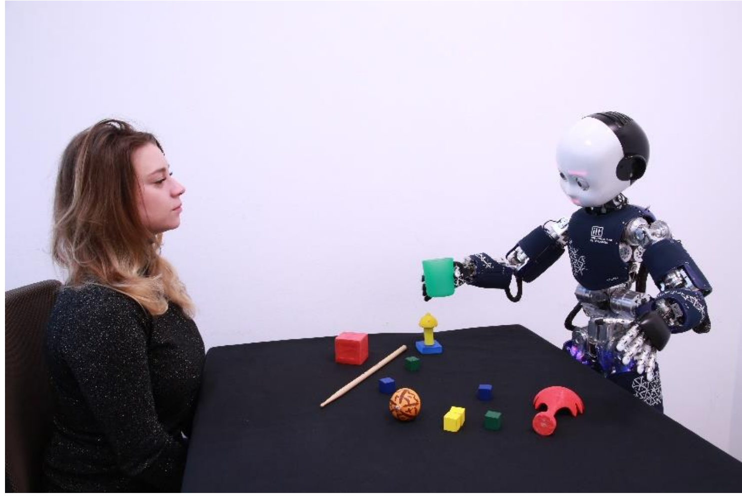

iCub afferra meglio gli oggetti cilindrici.

iCub pensa che alla ragazza piaccia il bicchiere.

M= 50.28; SD: 41.71

Item 4

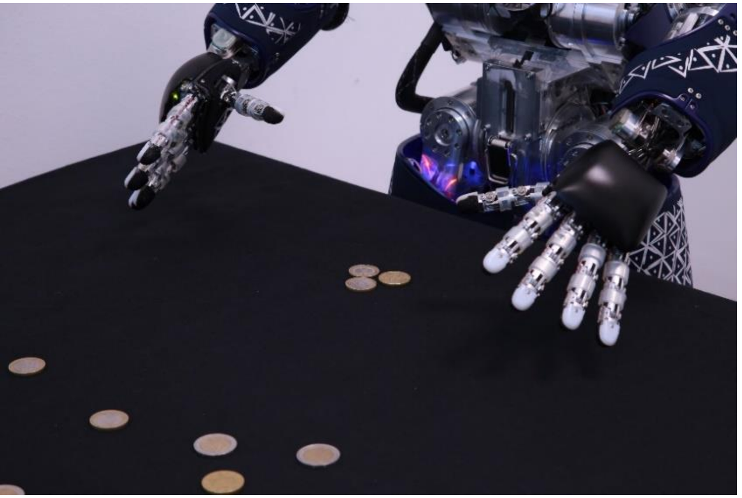
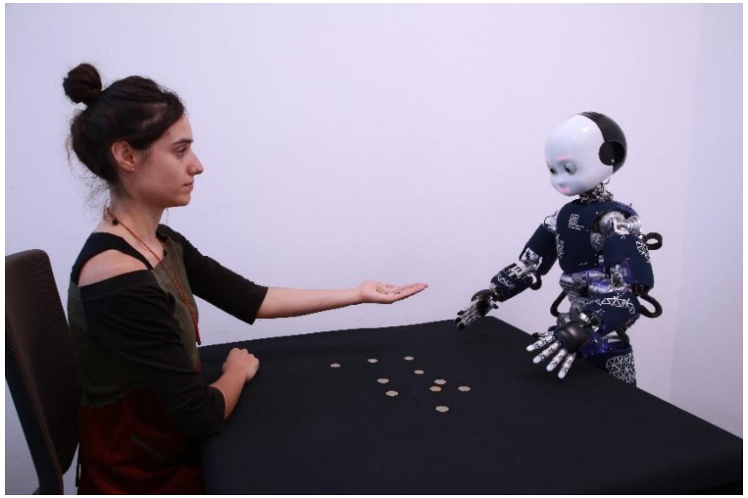
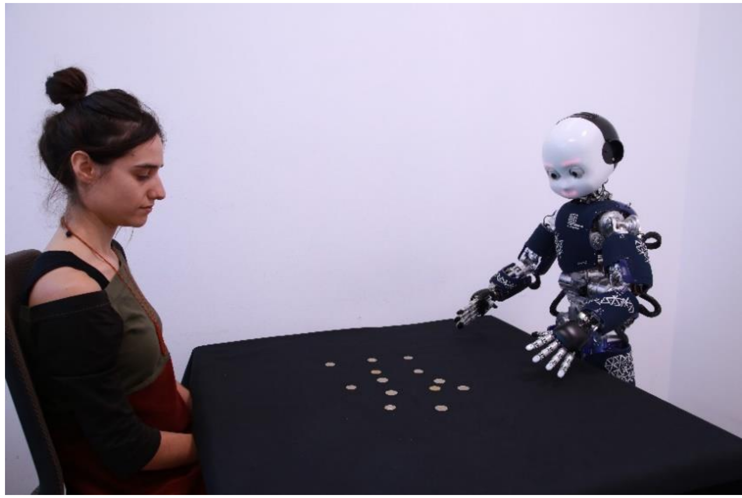

iCub calcola il numero di monete.

iCub ha deciso di restituire le monete.

M= 31.51; SD: 35.74

Item 5

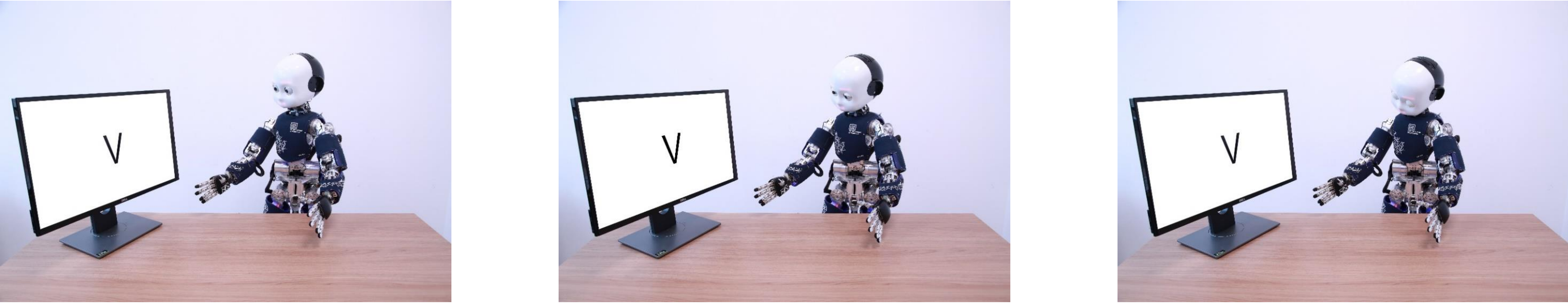

iCub è in fase di spegnimento.

iCub è annoiato.

M= 31.45; SD: 38.51

Item 6

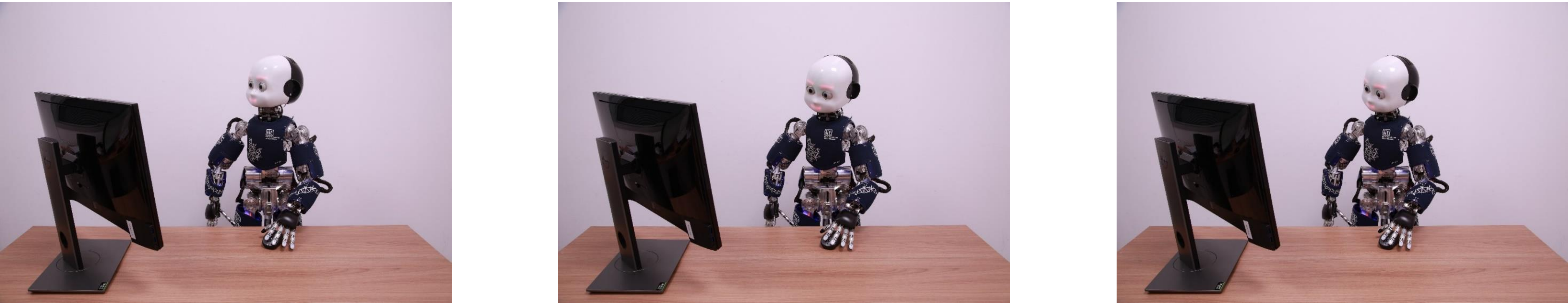

iCub traccia il cursore del mouse.

iCub è interessato a quello che c'è sullo schermo.

M= 43.46; SD: 40.89

Item 7

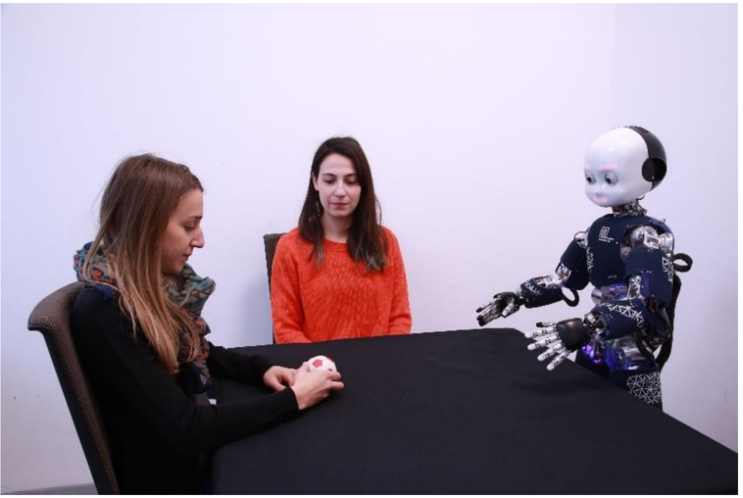

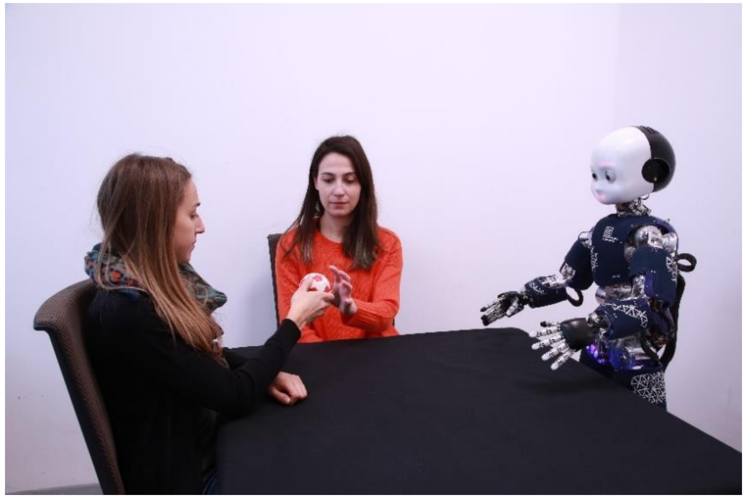

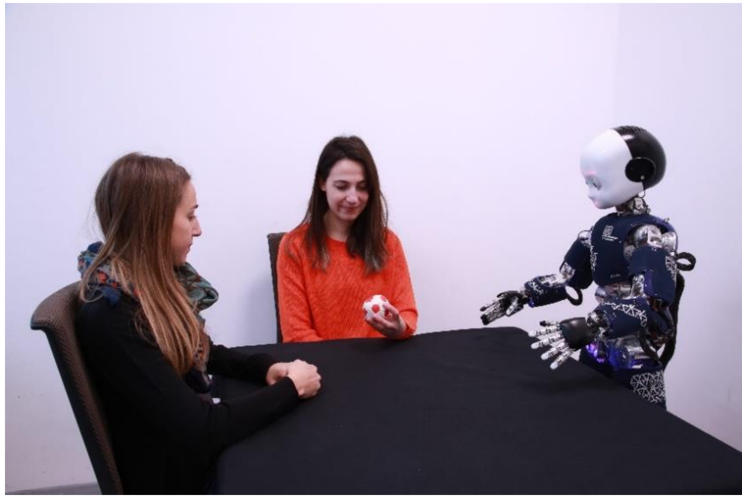

iCub traccia la posizione della palla.

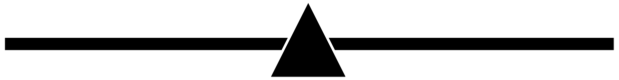

iCub non vede l’ora di ricevere la palla.

M= 33.27; SD: 38.11

Item 8

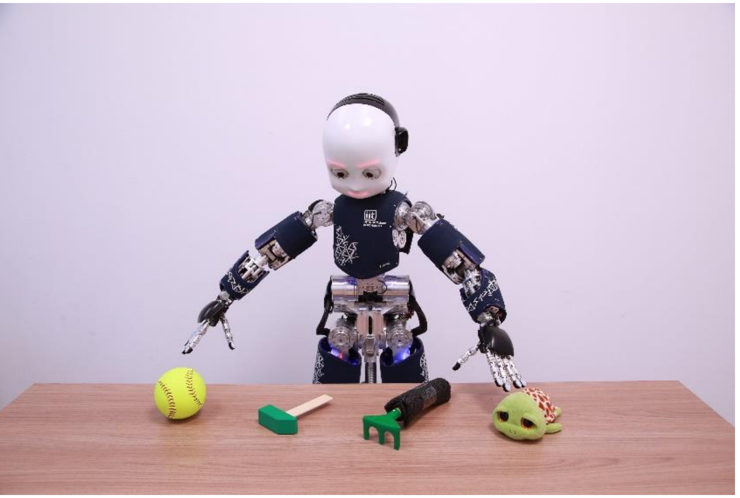

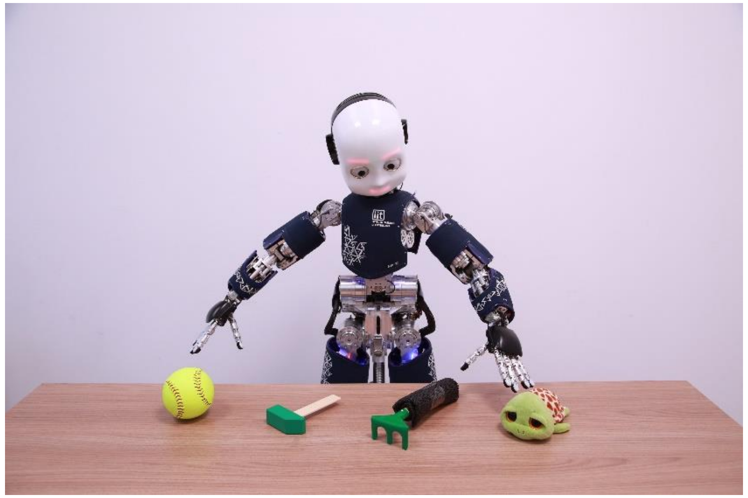

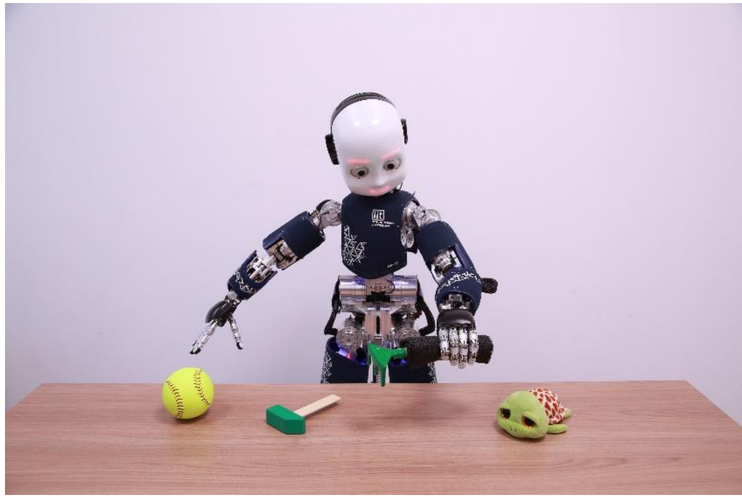

iCub regola la forza in base al peso dell’oggetto.

iCub finge di essere un giardiniere.

M= 22.84; SD: 30.21

Item 9

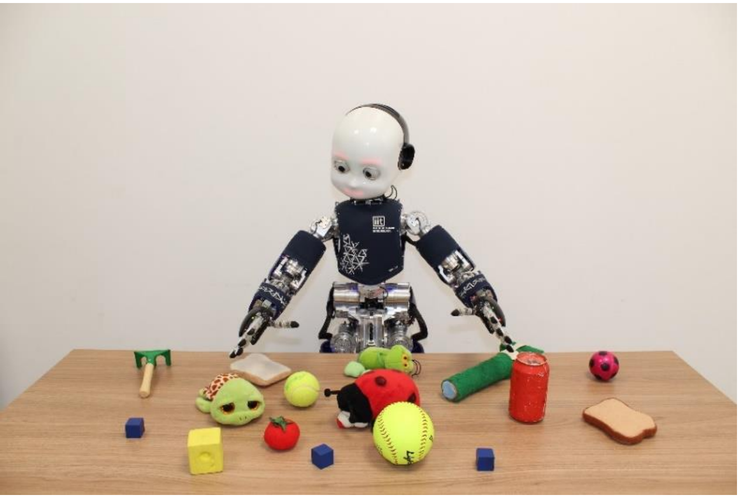

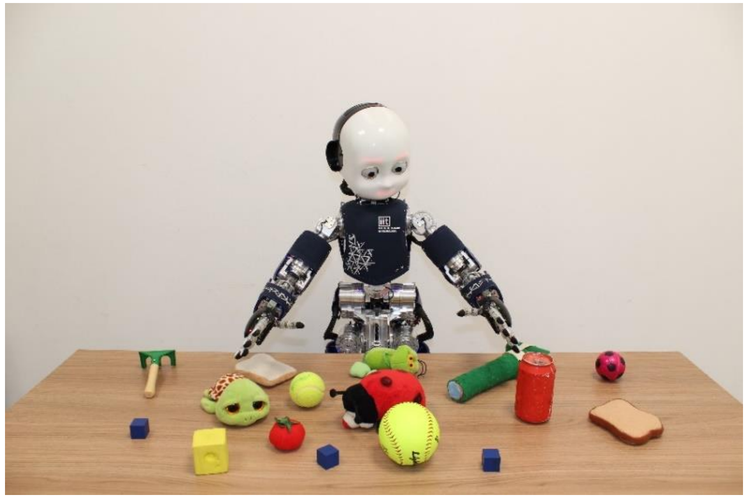

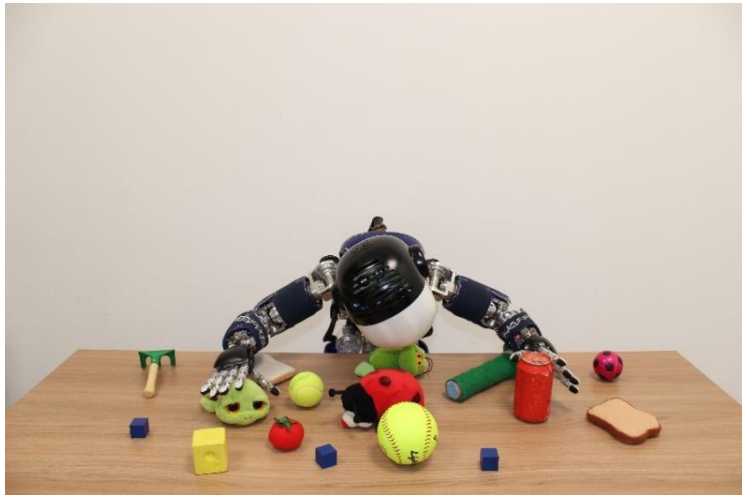

iCub ha i motori rotti.

iCub è disperato perché non trova il suo giocattolo preferito.

M= 55.72; SD: 41.15

Item 10

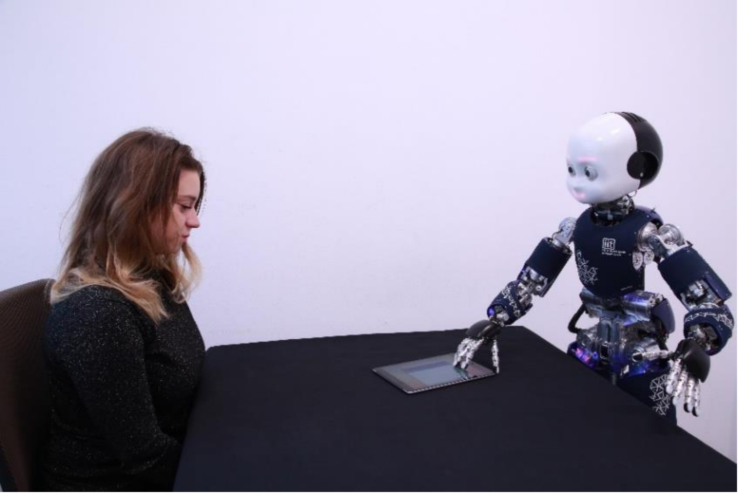

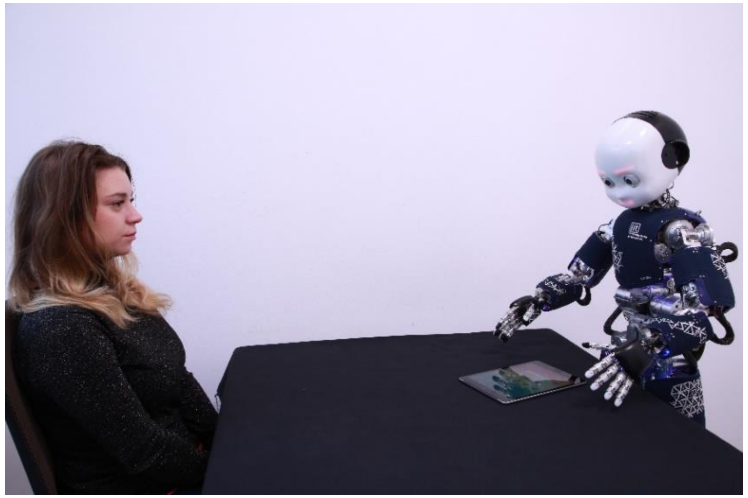

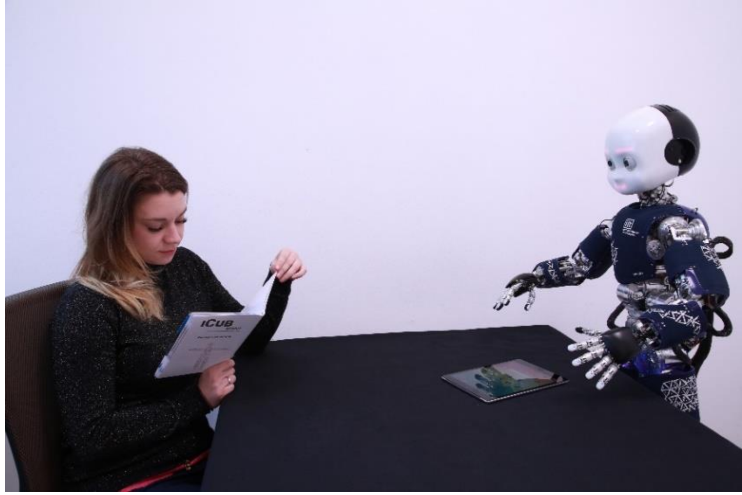

iCub rileva differenze tra il vecchio e il nuovo oggetto in scena.

iCub vuole leggere il libro.

M= 32.35; SD: 37.37

Item 11

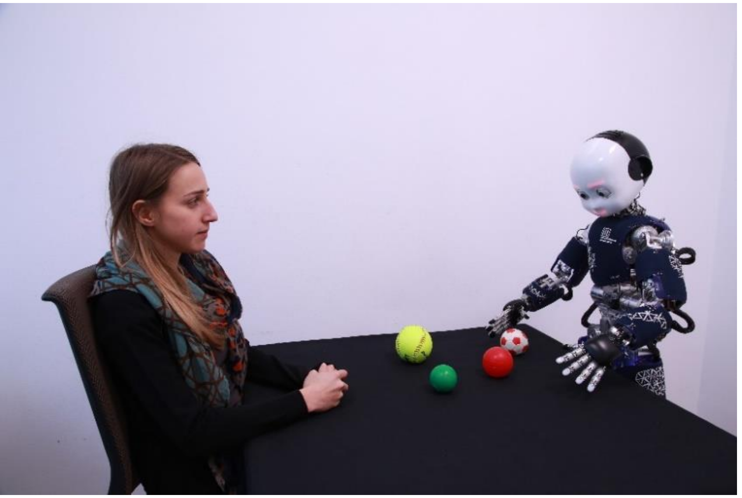

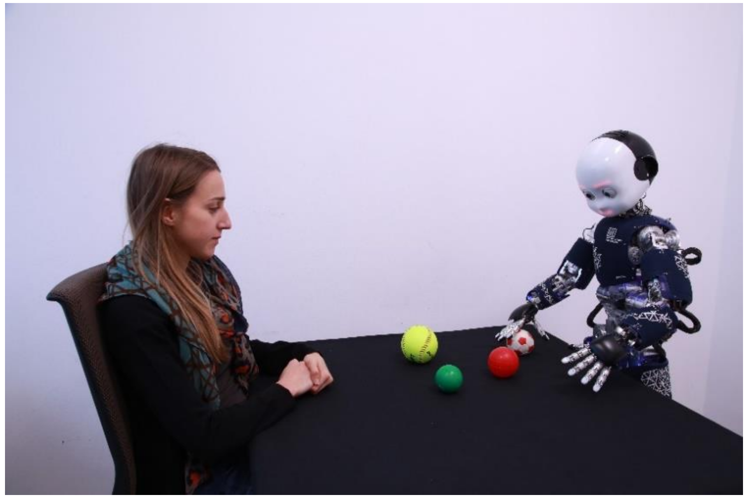

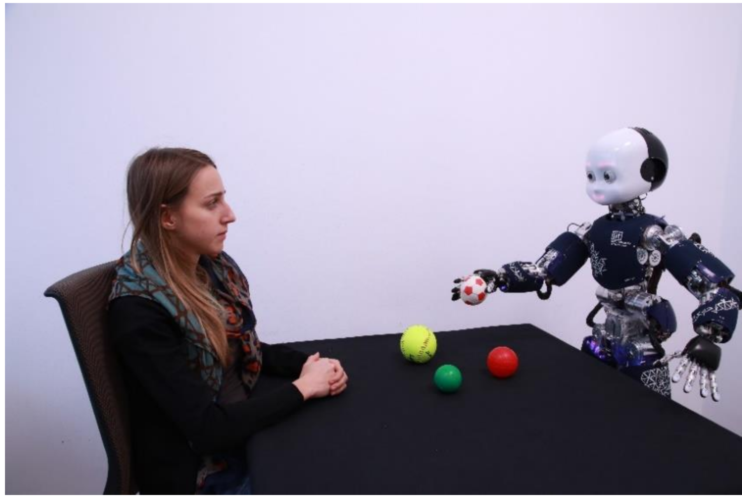

iCub calcola il peso delle palle.

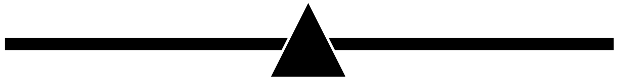

iCub vuole giocare con la ragazza.

M= 66.05; SD: 39.47

Item 12

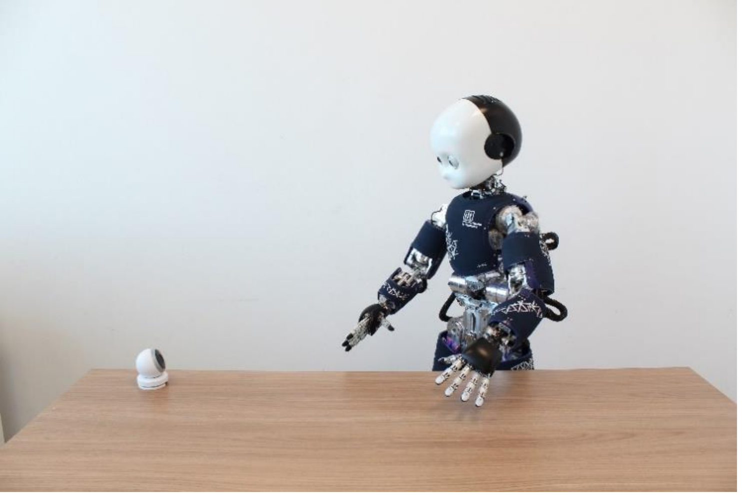

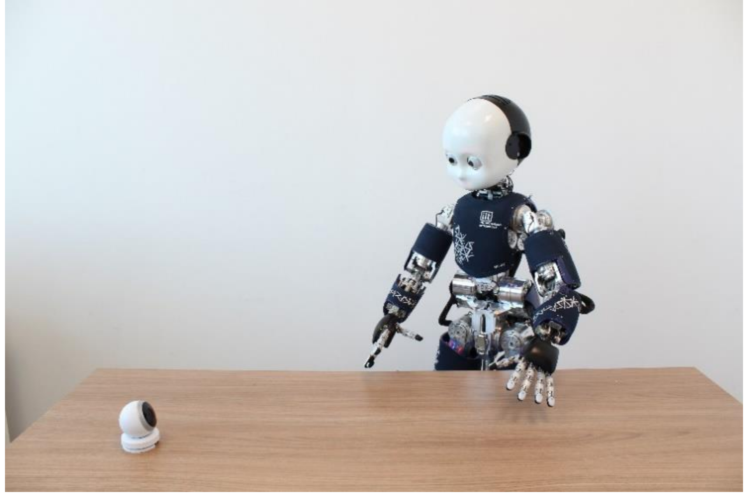

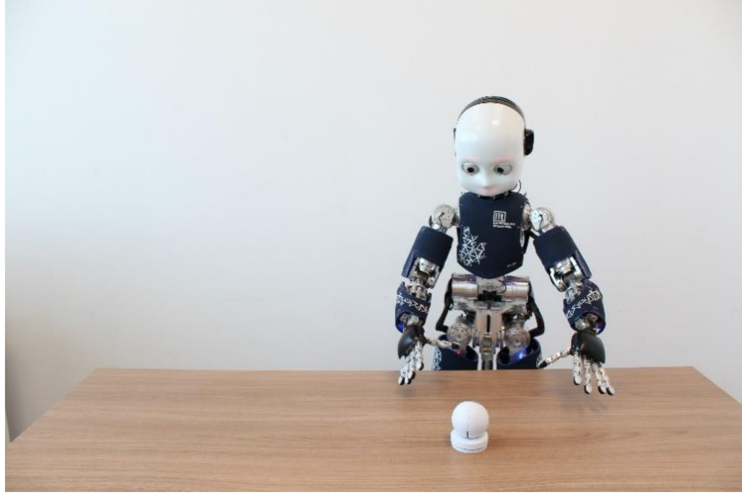

iCub allinea la testa con la webcam.

iCub è sorpreso dalla webcam che si muove.

M= 28.56; SD: 35.14

Item 13

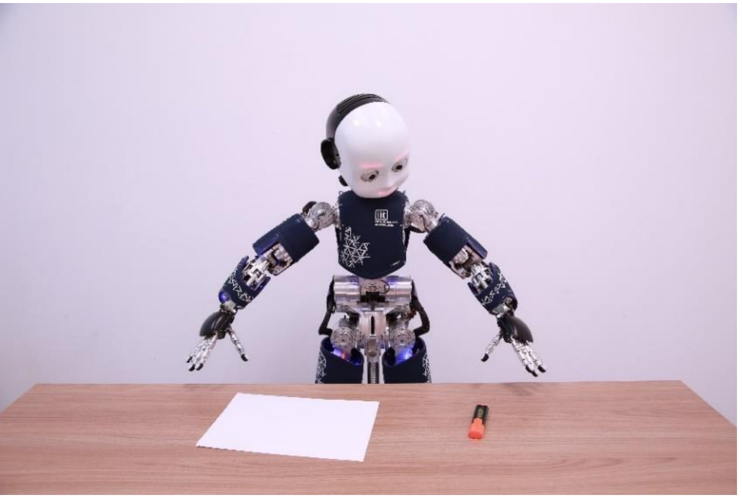

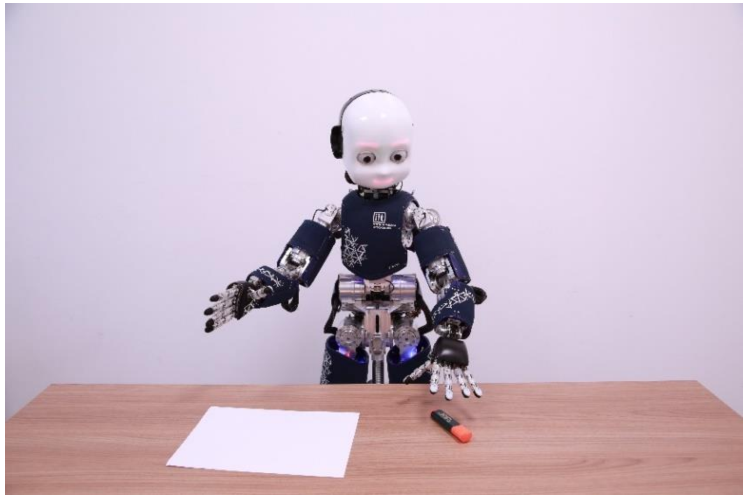

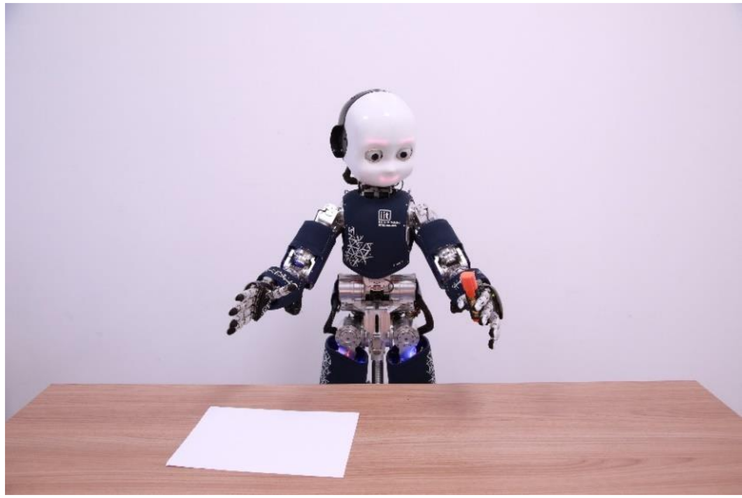

iCub ottimizza la presa gli oggetti piccoli.

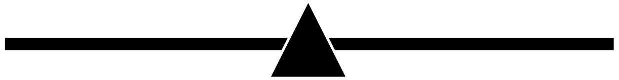

iCub vuole disegnare qualcosa.

M= 42.65; SD: 40.26

Item 14

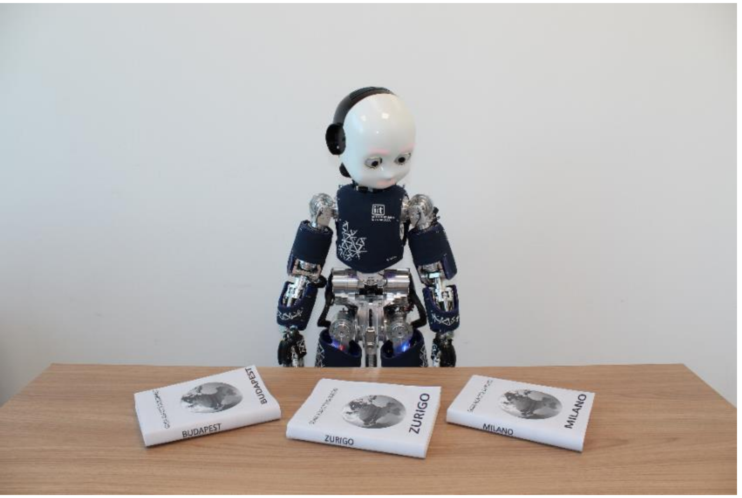

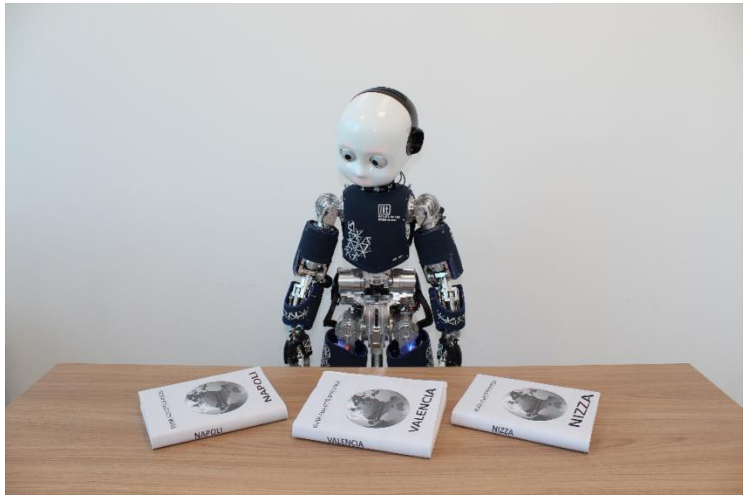

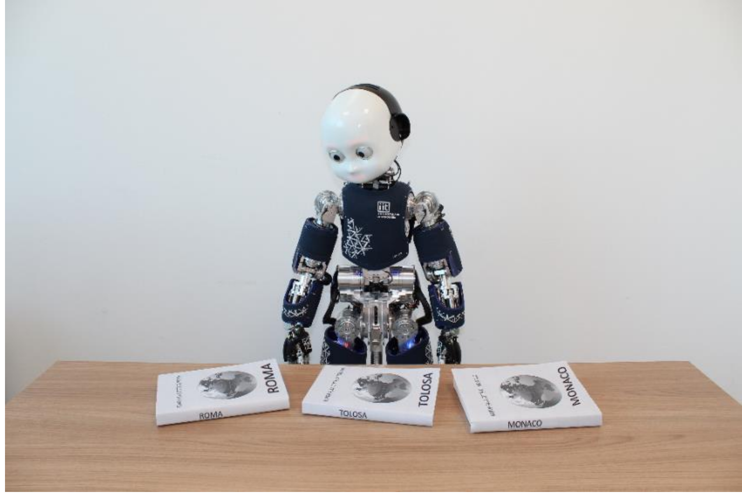

iCub registra le città italiane in ordine alfabetico.

iCub preferisce le città italiane.

M= 41.85; SD: 40.47

Item 15

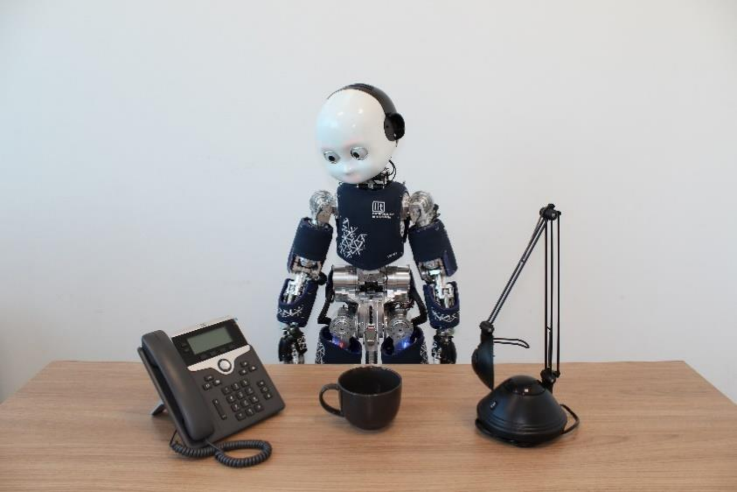
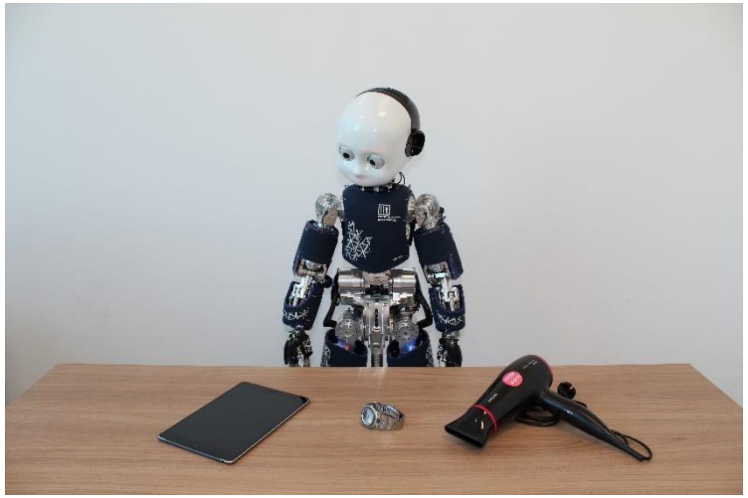
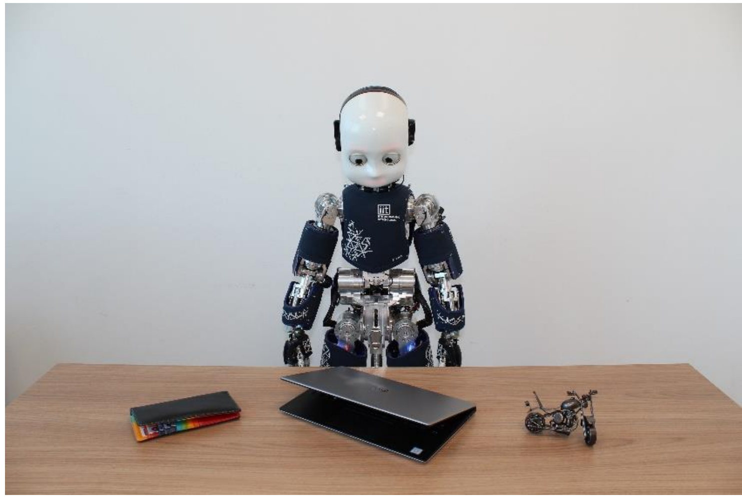

iCub rileva facilmente gli oggetti con uno schermo.

iCub è incuriosito dalla tecnologia digitale.

M= 33.79; SD: 38.01

Item 16

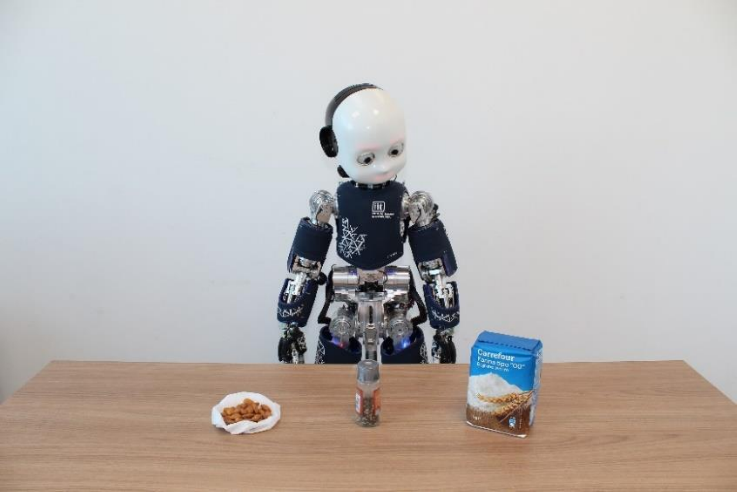

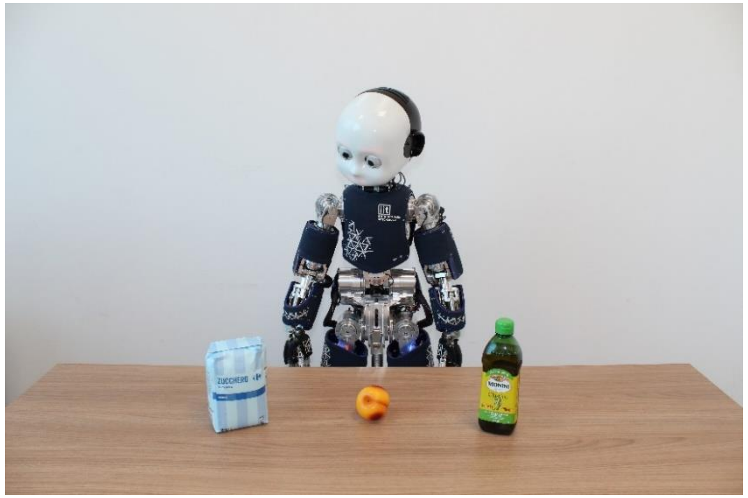

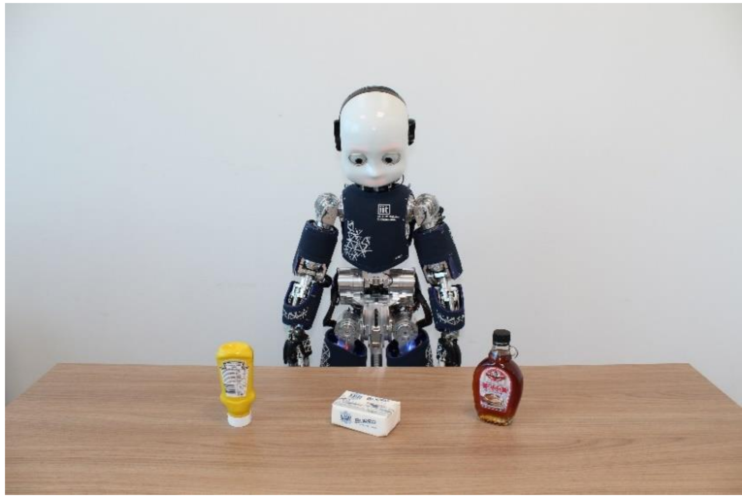

iCub segue le istruzioni della ricetta.

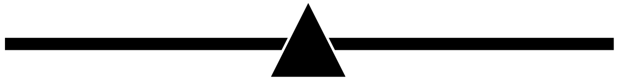

iCub ha deciso di cucinare una torta.

M= 33.28; SD: 36.97

Item 17

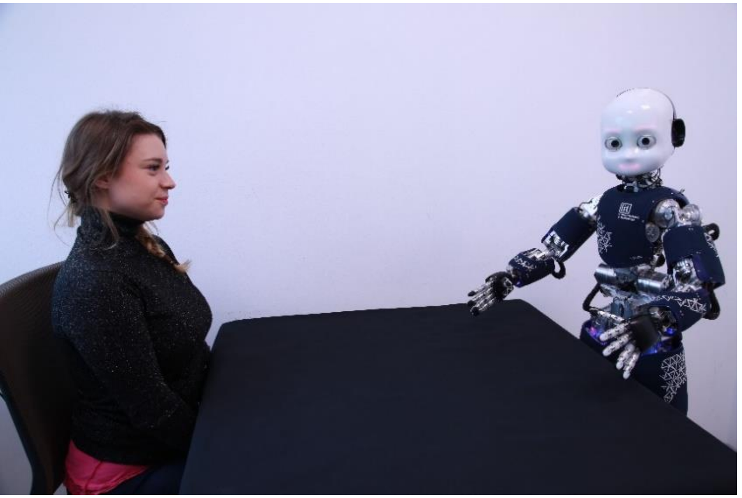

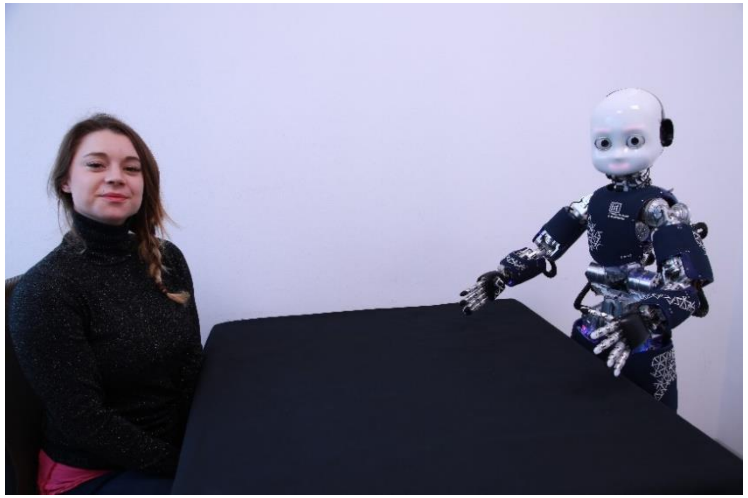

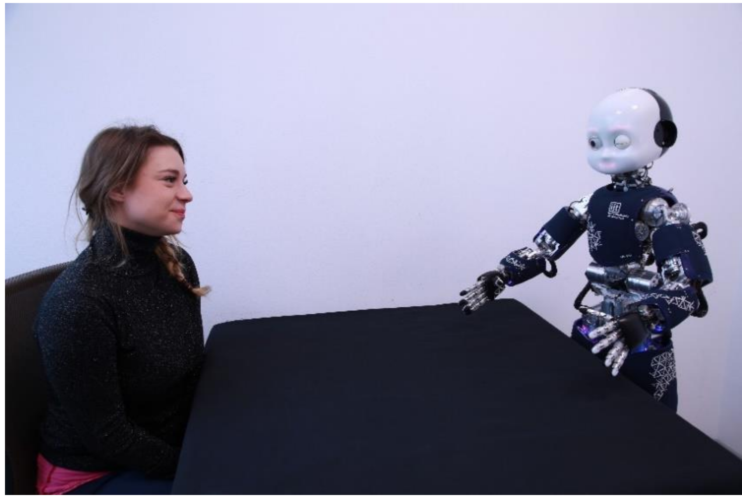

iCub ha i motori
delle palpebre rotti.

iCub cerca di essere
affascinante.

M= 65.04; SD: 38.54

Item 18

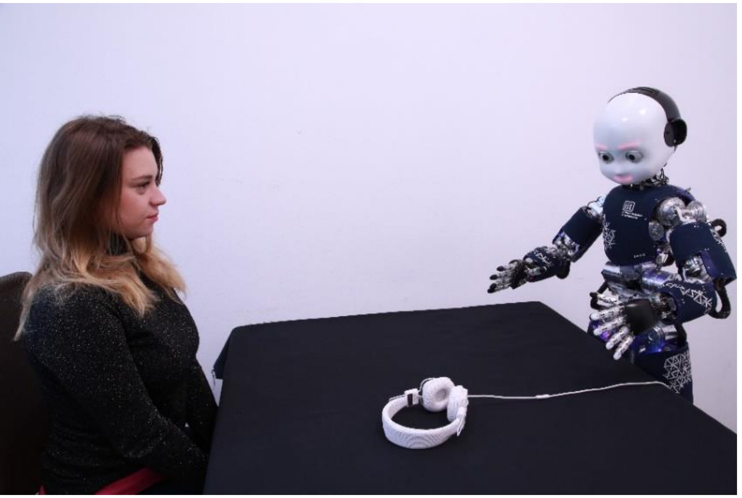

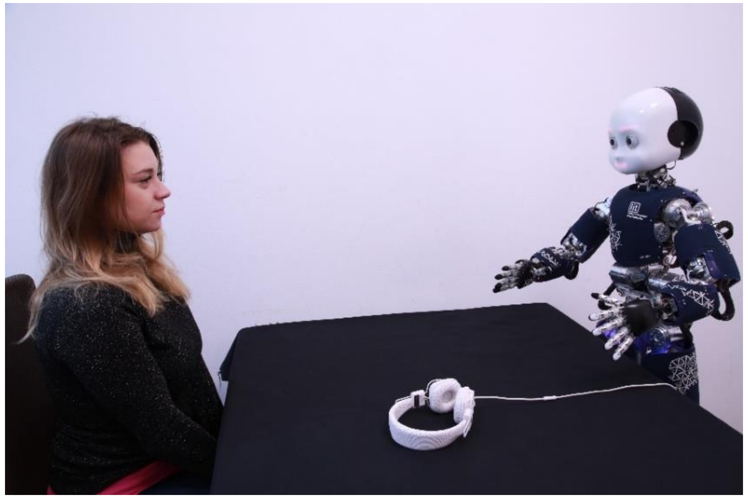

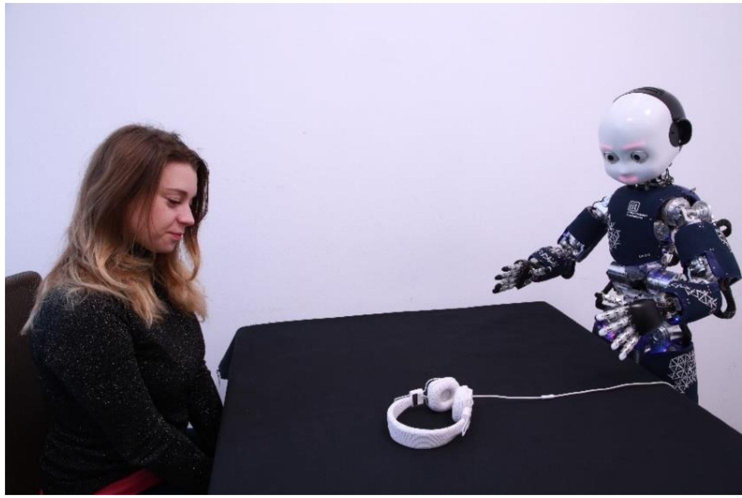

iCub misura la distanza tra la ragazza e le cuffie.

iCub si aspetta che la ragazza dia in prestito le cuffie.

M= 38.09; SD: 38.33

Item 19

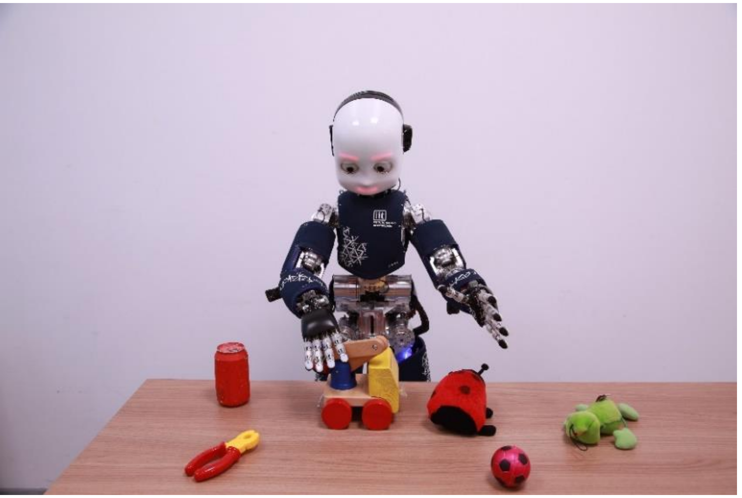

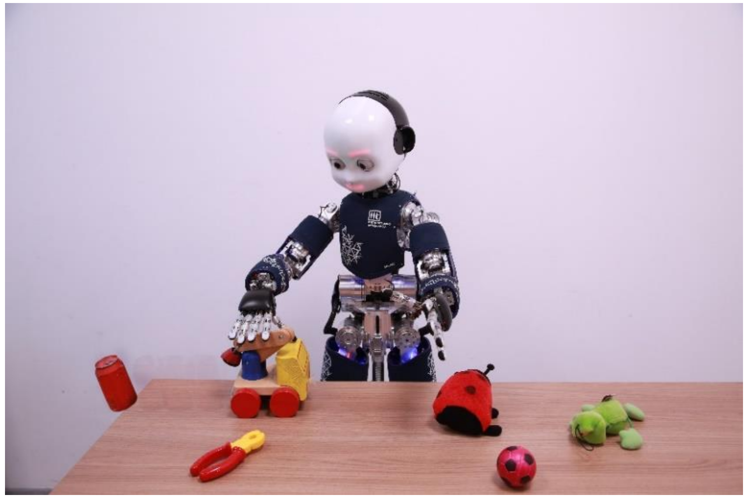

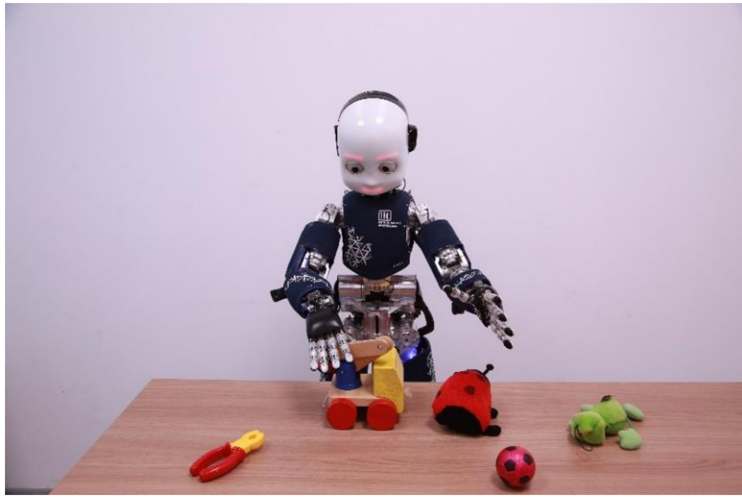

iCub non può bloccare la procedura nonostante la lattina stia cadendo.

iCub non ha intenzione di raccogliere la lattina caduta.

M= 24.71; SD: 31.91

Item 20

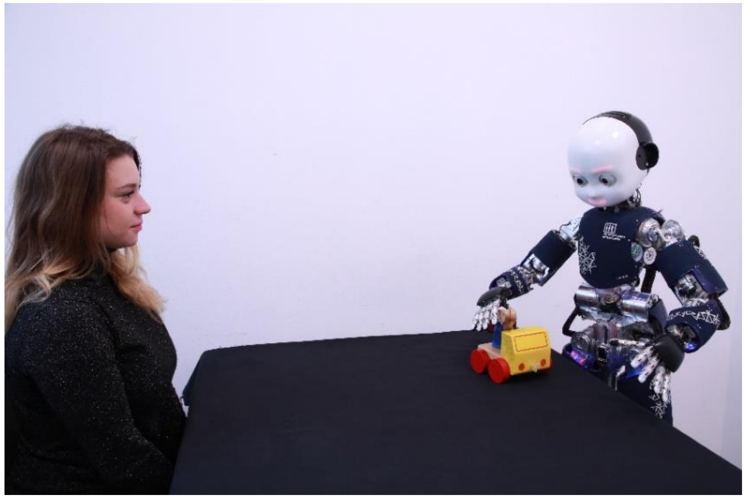

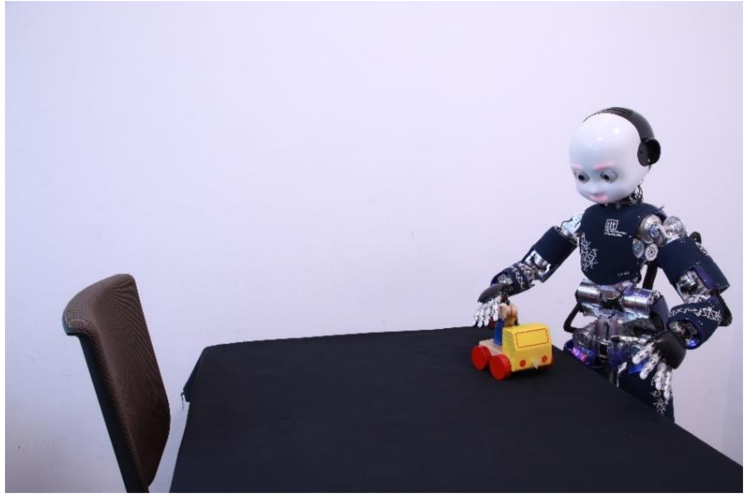

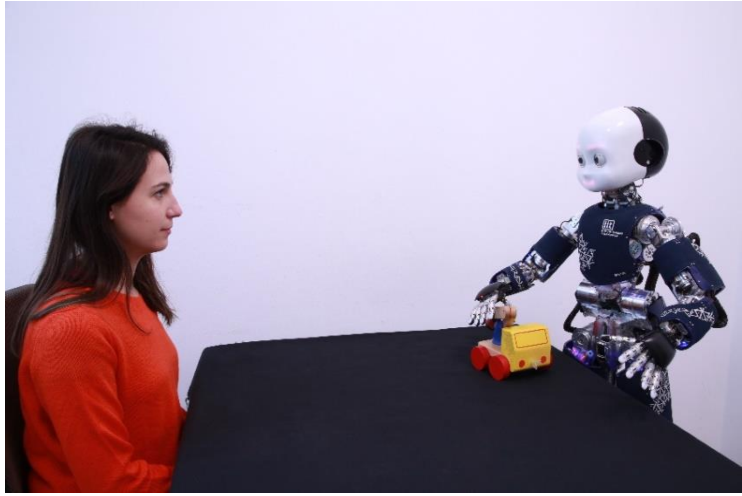

iCub muove la testa verso i maglioni dai colori luminosi.

iCub è sorpreso che ci sia una nuova persona.

M= 56.58; SD: 39.13

Item 21

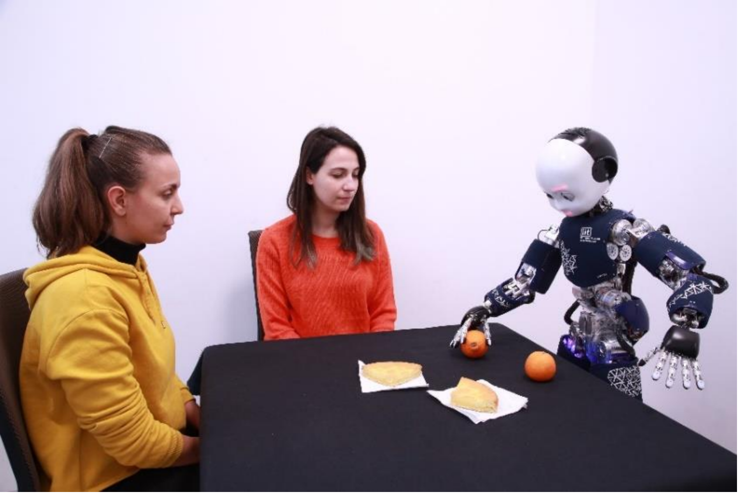

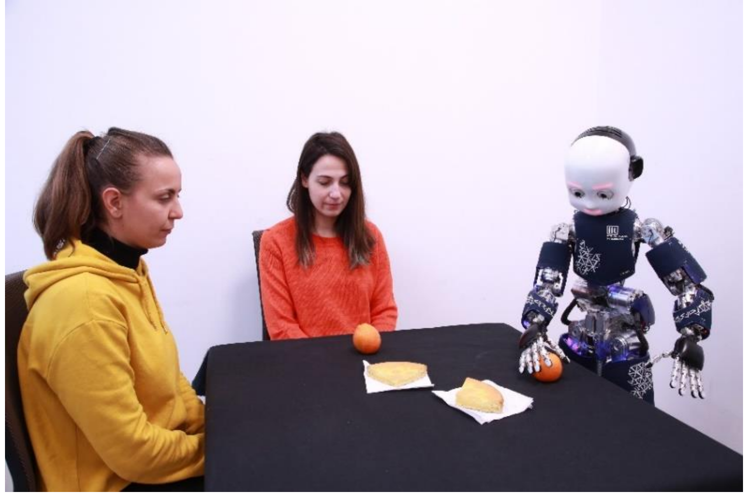

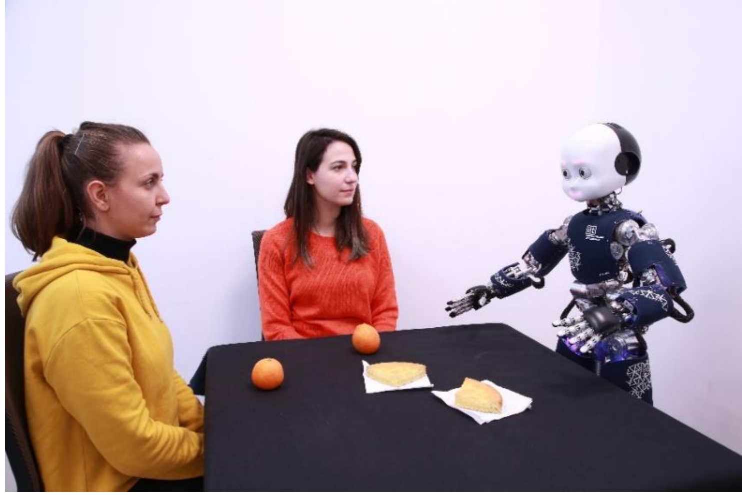

iCub pensa che le ragazze abbiano bisogno di cibo sano.

iCub può afferrare le arance più facilmente della torta.

M= 24.42; SD: 33.80

Item 22

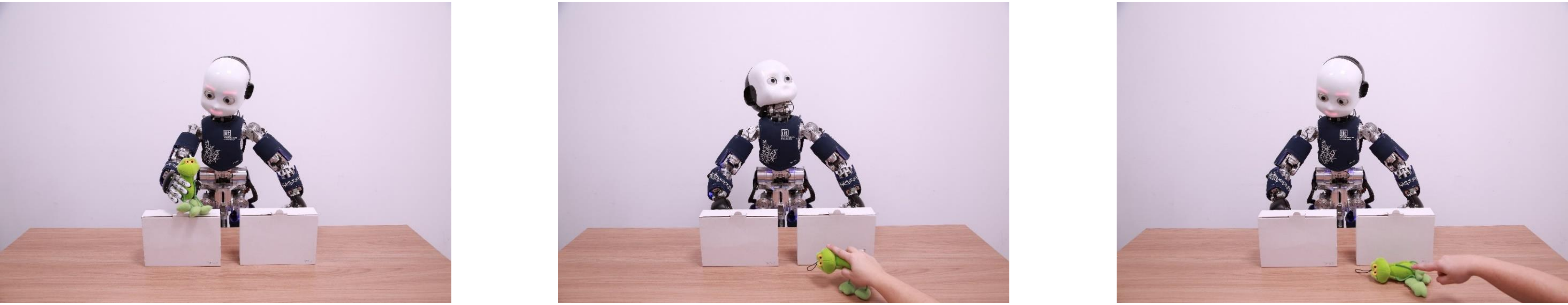

iCub riporta la testa nella posizione iniziale.

iCub non è più interessato al giocattolo.

M= 34.34; SD: 37.02

Item 23

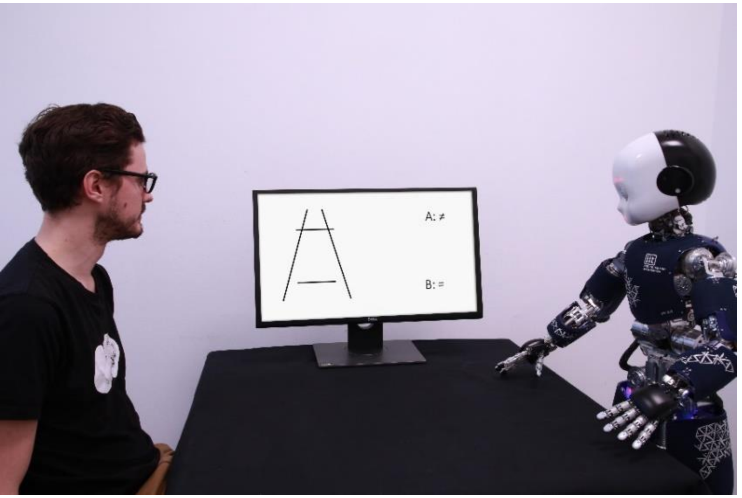

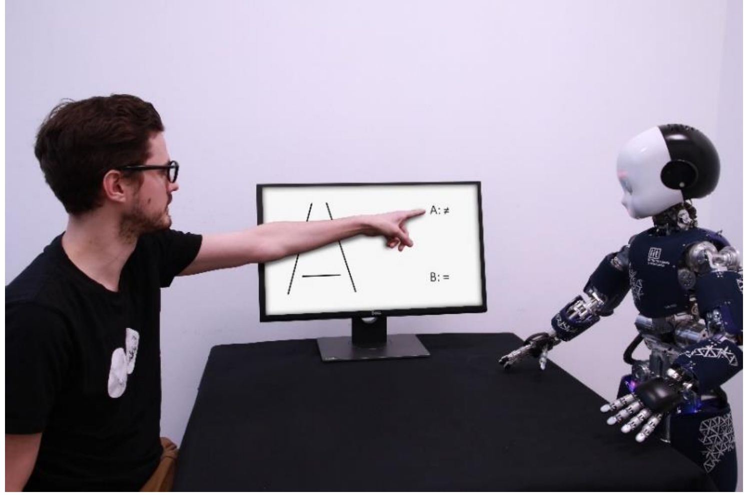

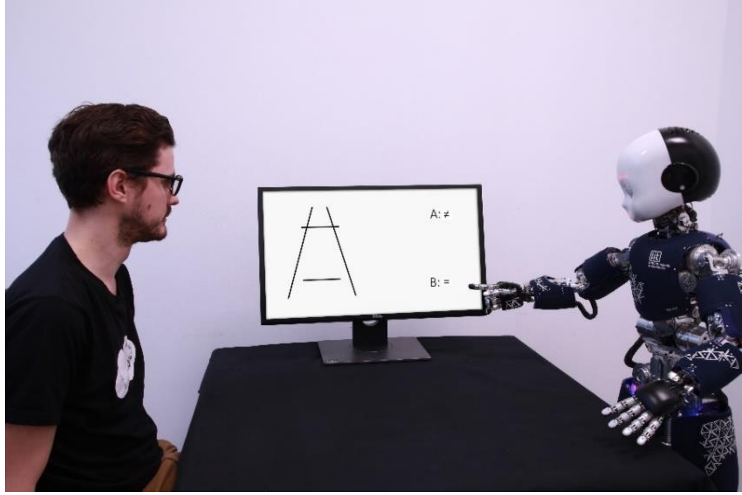

iCub sta ripetendo il gesto di indicare.

iCub sta esprimendo un’opinione.

M= 52.42; SD: 42.11

Item 24

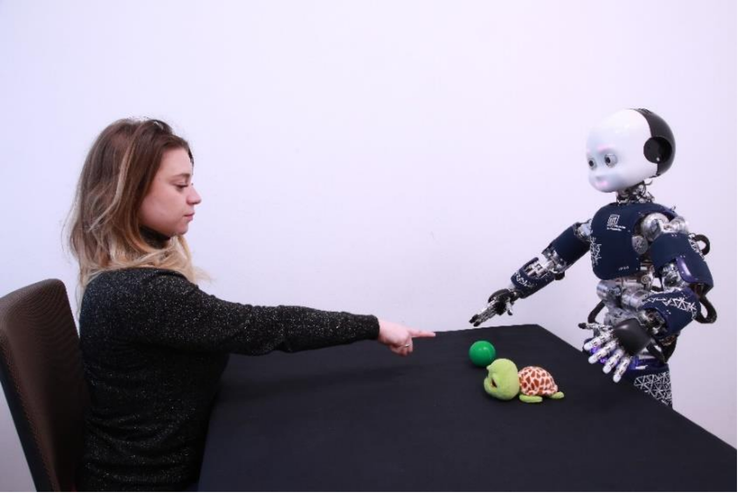

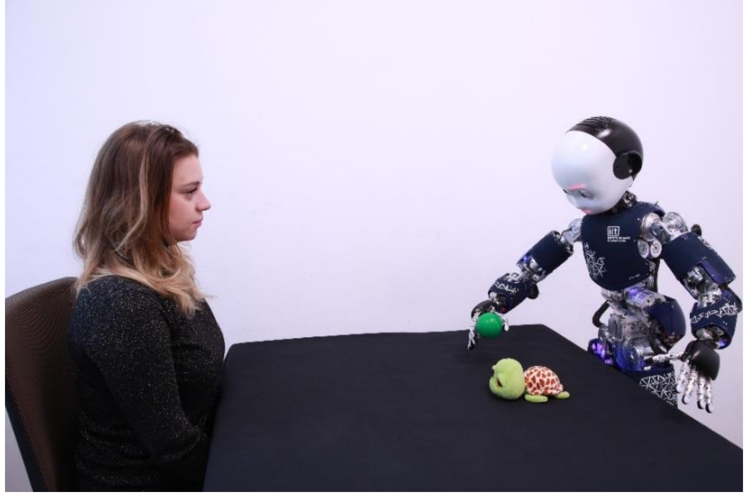

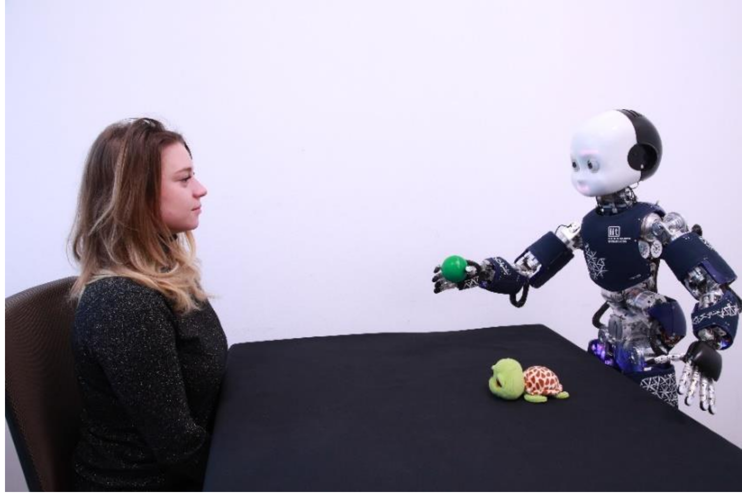

iCub traccia i movimenti della mano.

iCub ha capito che la ragazza vuole la palla.

M= 68.83; SD: 38.41

Item 25

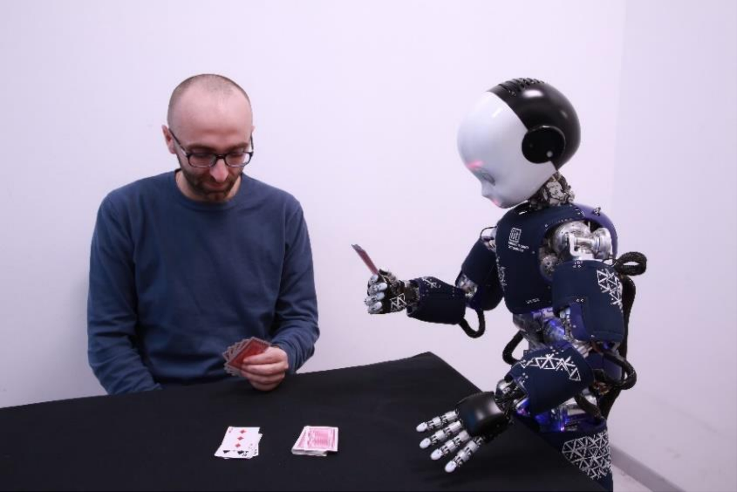

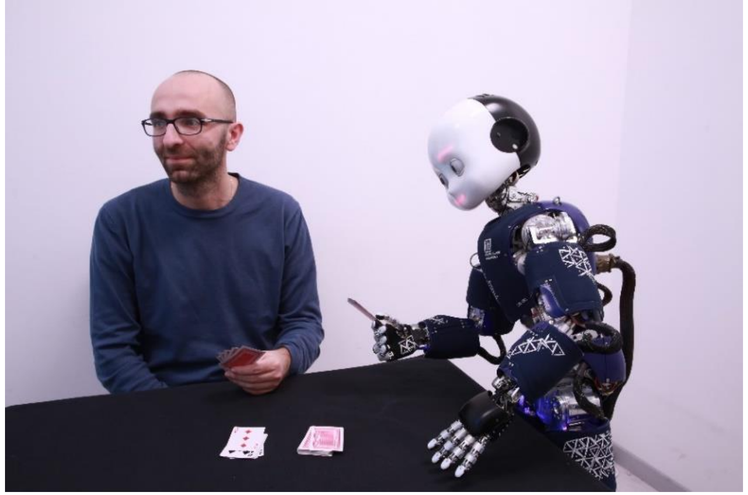

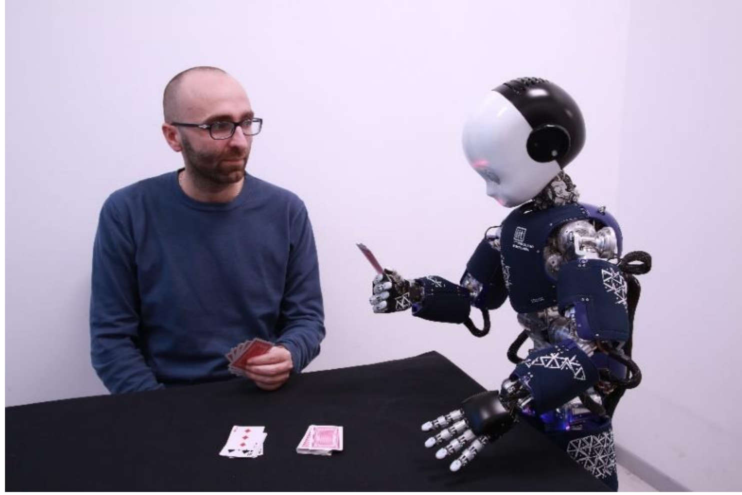

iCub bara guardando le carte dell’avversario.

iCub ha perso l'equilibrio per un momento.

M= 78.16; SD: 30.59

Item 26

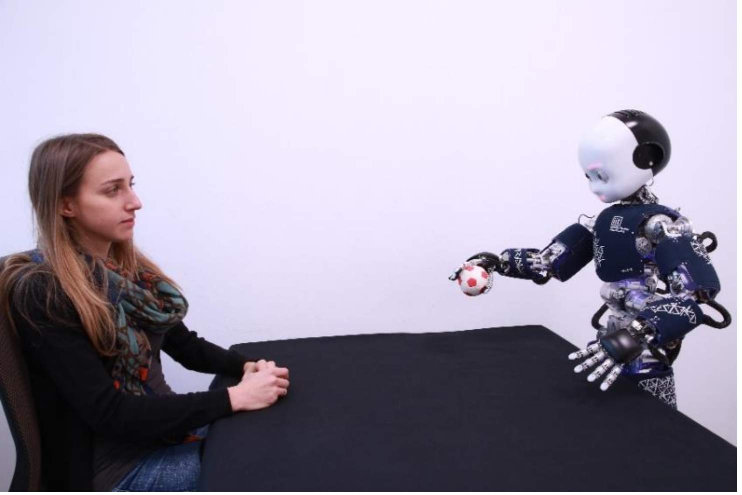

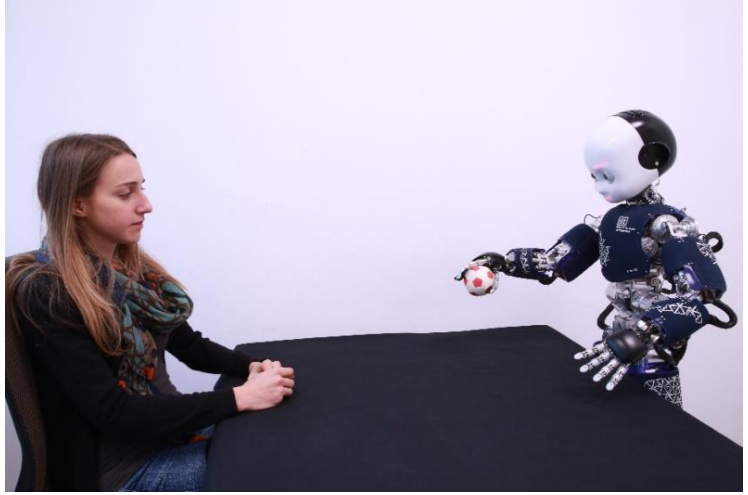

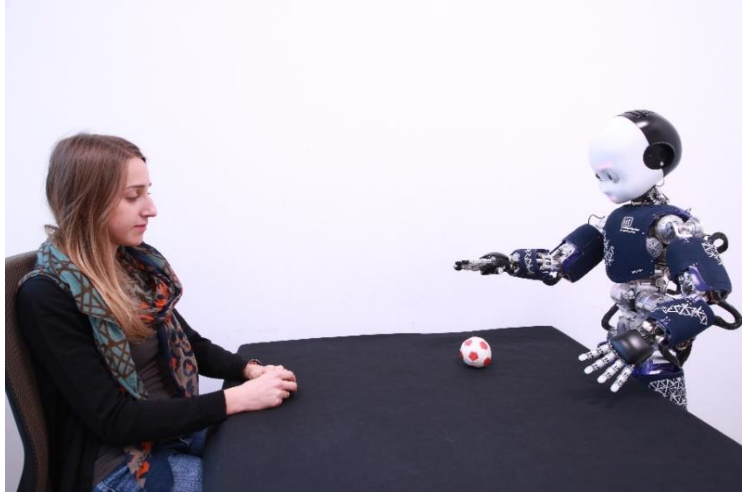

iCub ha un malfunzionamento alla mano.

iCub è deluso che la palla non rimbalzi.

M= 57.61; SD: 38.94

Item 27

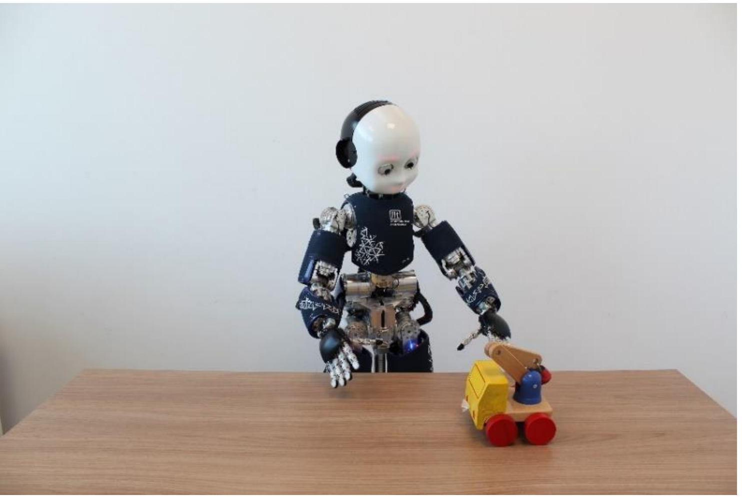

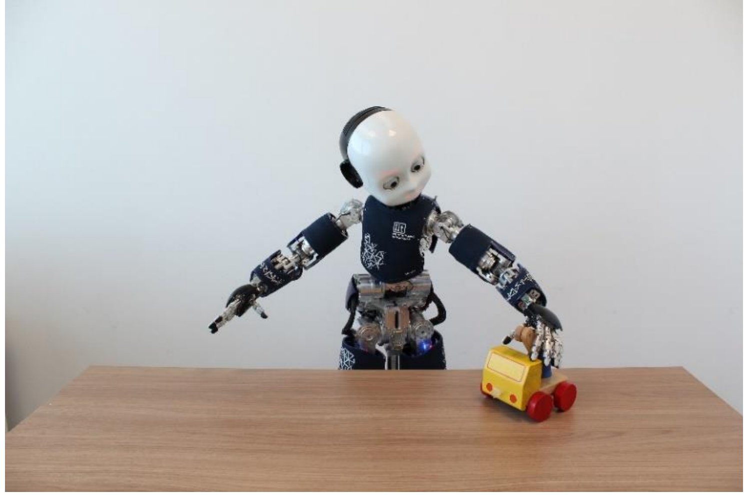

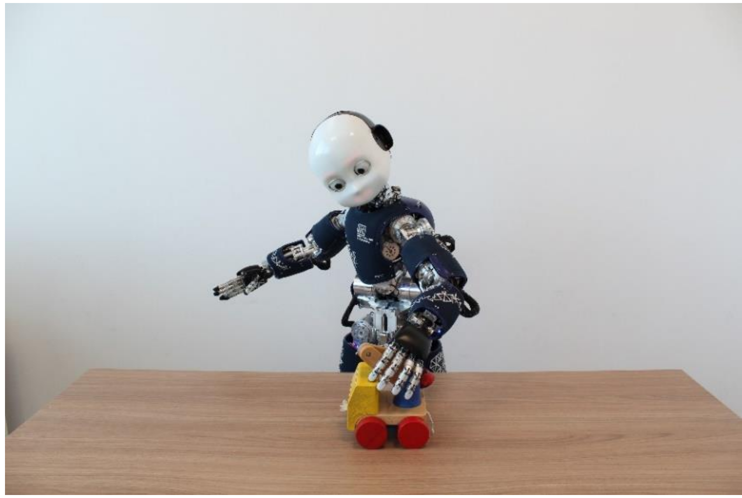

iCub allena la coordinazione testa-mano.

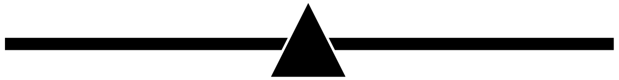

iCub si diverte a giocare con il camioncino.

M= 45.08; SD: 40.81

Item 28

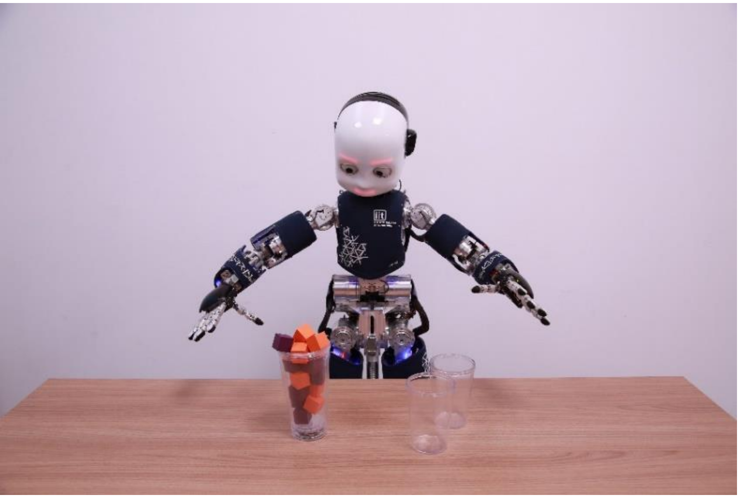

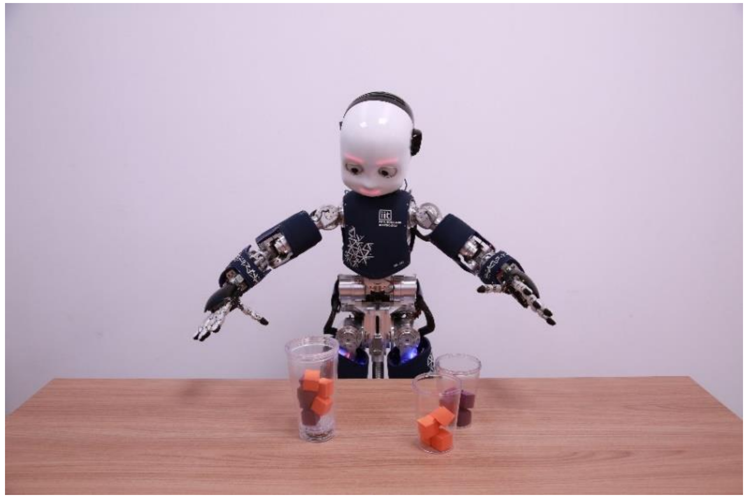

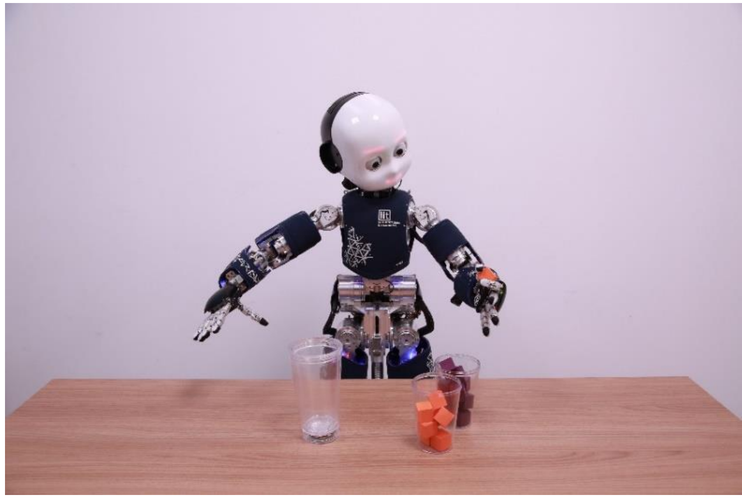

iCub classifica i cubi in base al colore.

iCub vorrebbe tenere questo cubo.

M= 10.07; SD: 20.62

Item 29

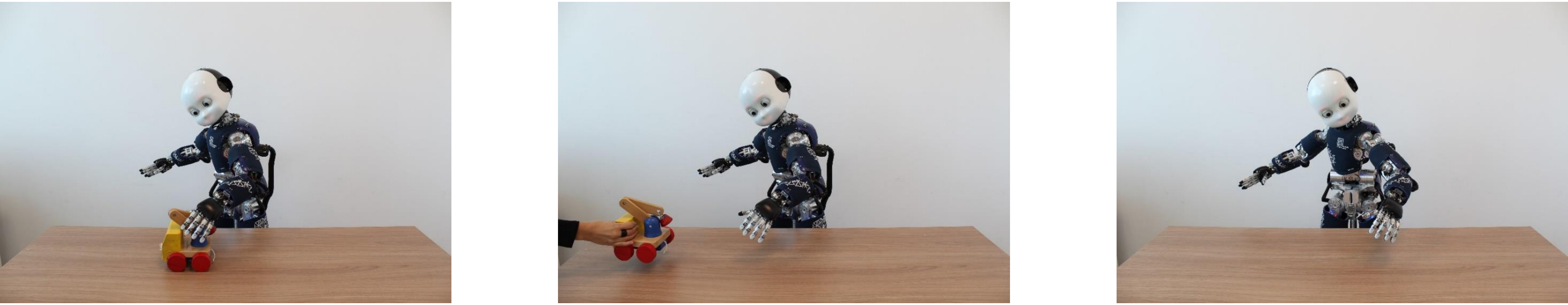

iCub è bloccato.

iCub immagina che il giocattolo sia ancora lì.

M= 48.55; SD: 41.41

Item 30

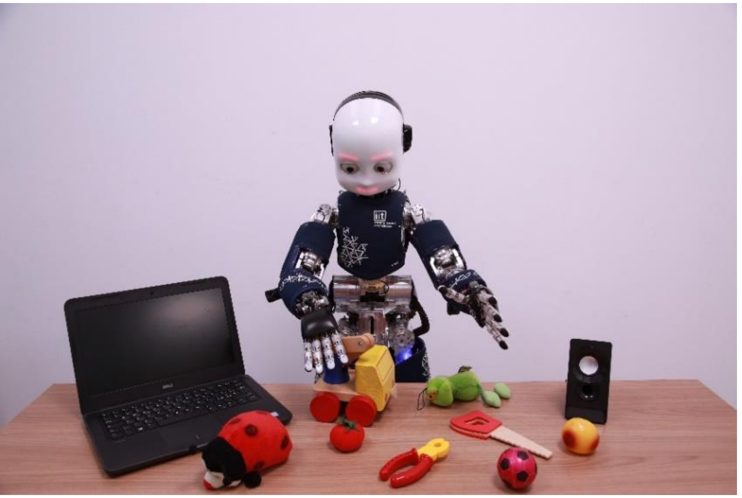

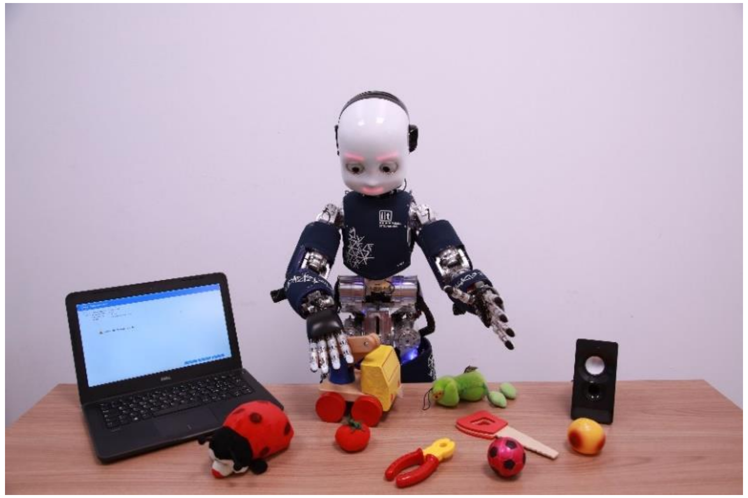

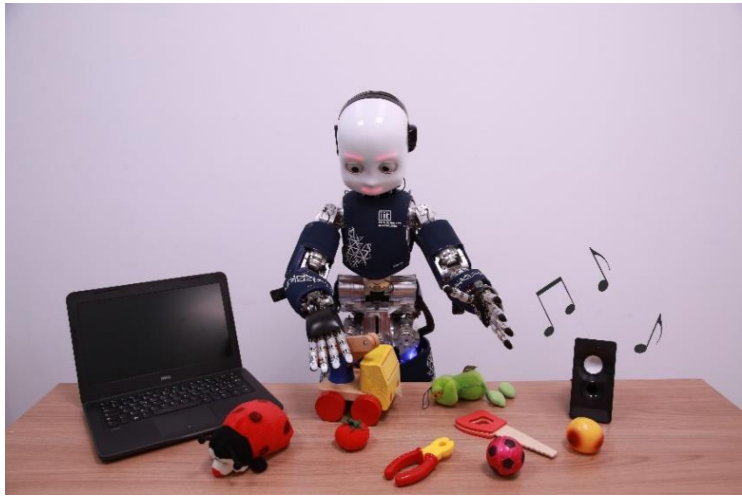

iCub è in modalità stand-by.

iCub non riesce a scegliere con quale giocattolo giocare.

M= 25.10; SD: 34.34

Item 31

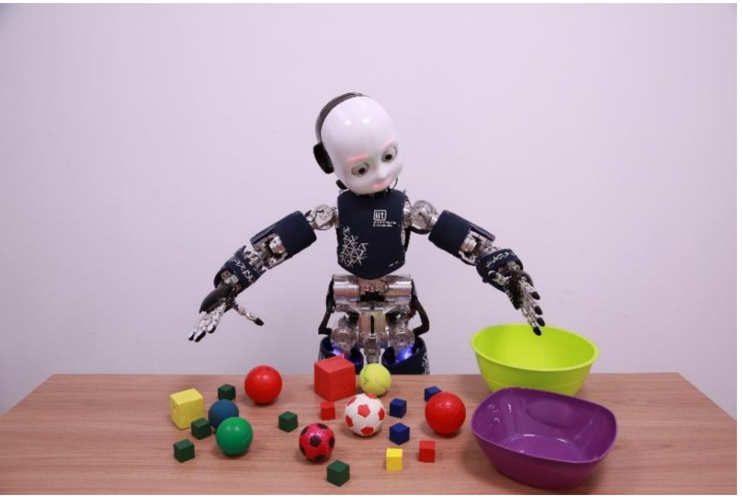

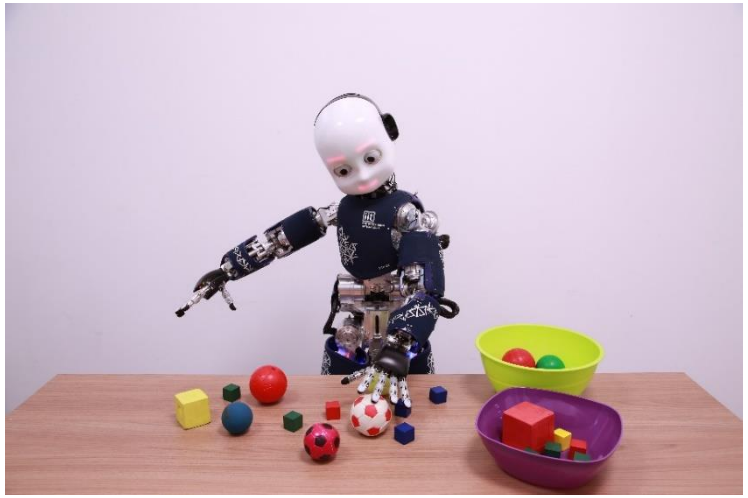

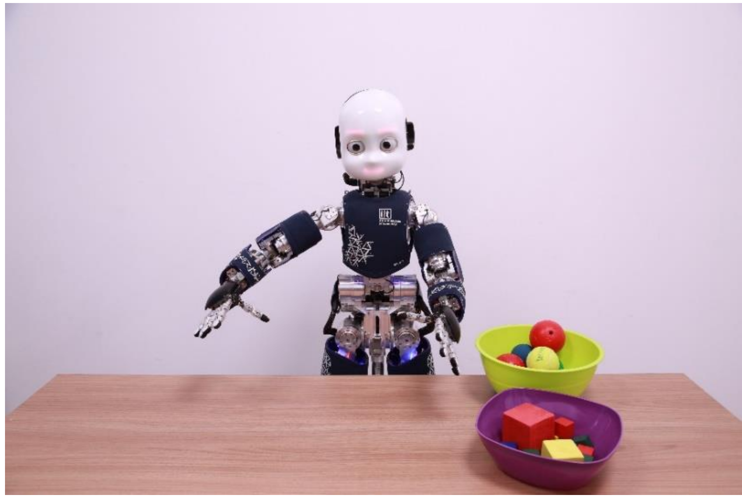

iCub mette gli oggetti nel contenitore con la forma corrispondente.

iCub sa che è giusto mettere in ordine il tavolo dopo aver giocato.

M= 11.97; SD: 25.22

Item 32

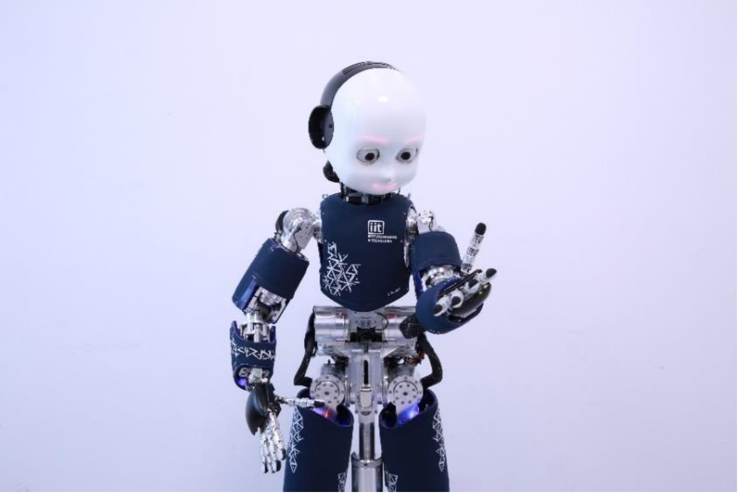

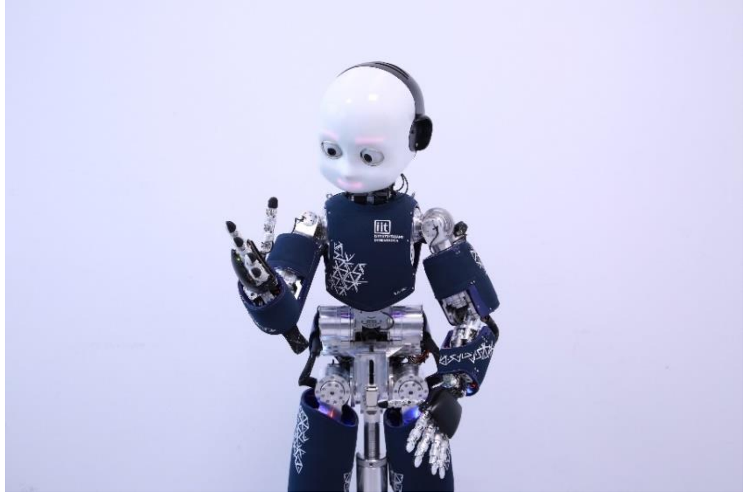

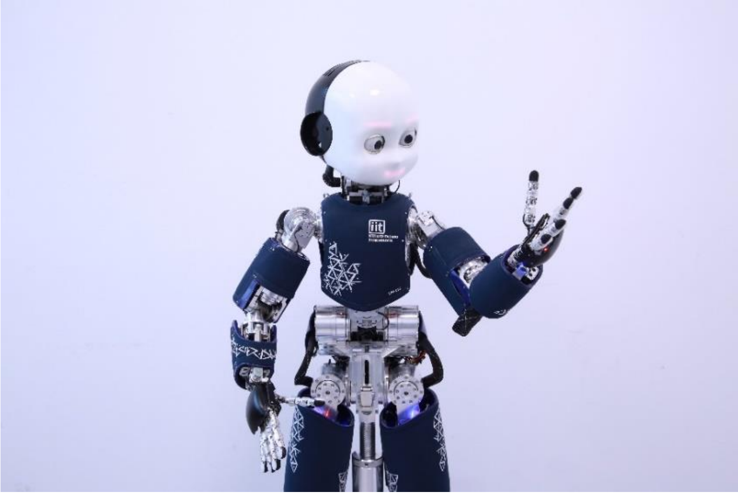

iCub calibra la posizione della testa con quella della mano.

iCub si diverte contando le dita su ciascuna mano.

M= 34.21; SD: 38.33

Item 33

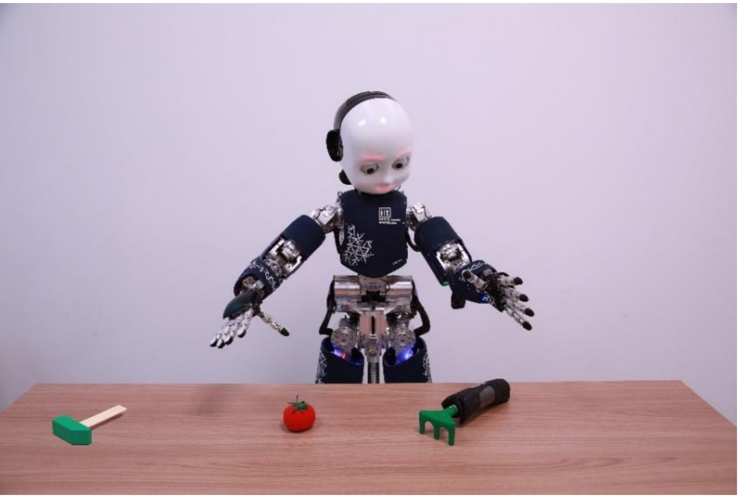
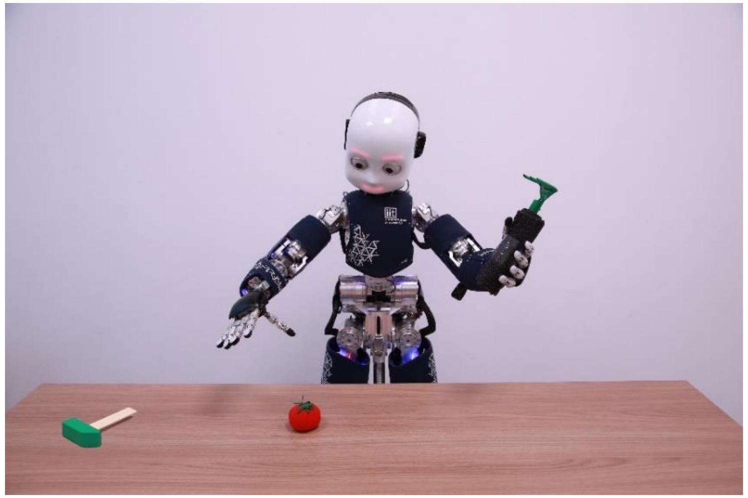
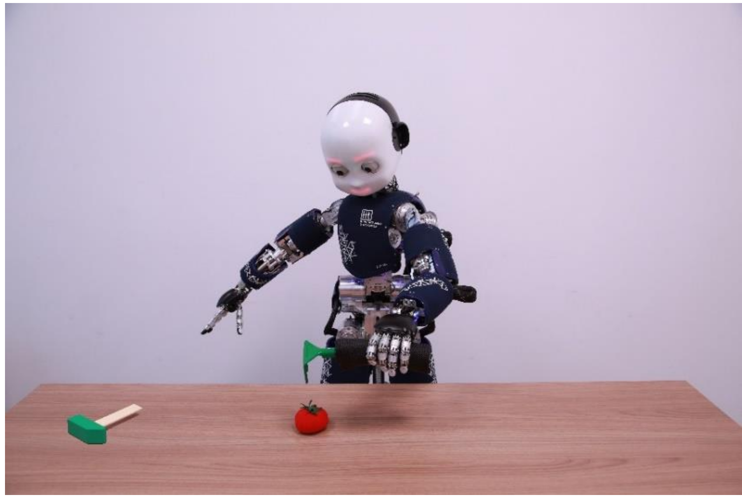

iCub afferra l’oggetto più vicino.

iCub è affascinato dall’uso degli utensili.

M= 46.40; SD: 41.53

Item 34

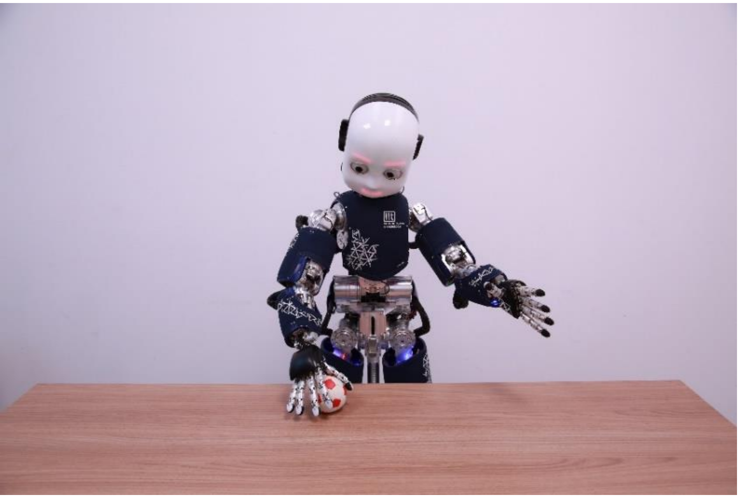

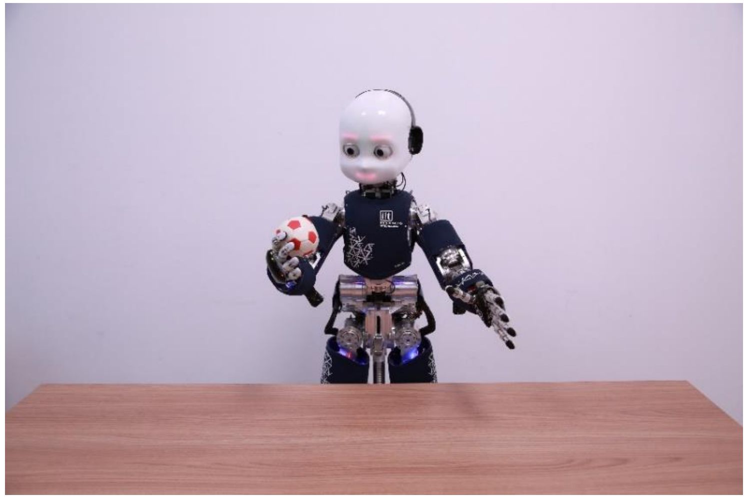

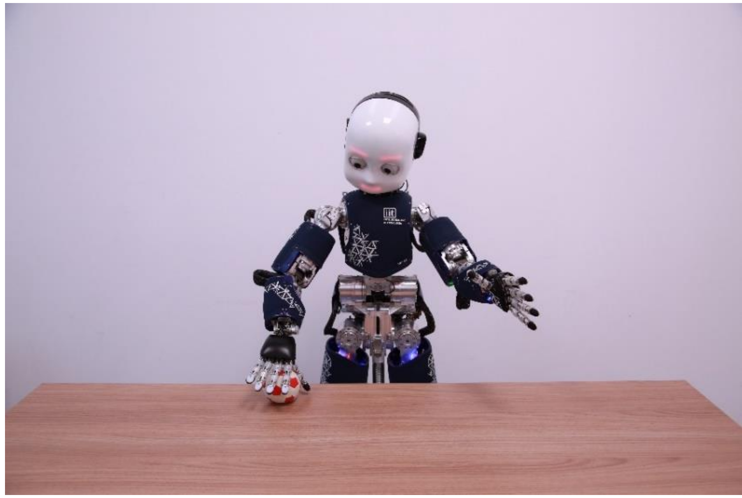

iCub aggiorna le coordinate della posizione della palla.

iCub ha deciso di non lanciare la palla.

M= 34.40; SD: 36.91

Demographics

17% completato

Prima di procedere con l'inizio del questionario ti preghiamo di rispondere ad alcune domande utili allo svolgimento dello studio.

Il questionario è totalmente anonimo. Il Codice Identificativo è necessario per mantenere tale anonimato. Puoi inserire una stringa di lettere e numeri a tua scelta.

Codice Identificativo	
Sesso (M/F)	
Età	
Numero Figli	
Numero Fratelli/Sorelle	
Occupazione	

Scolarità (In caso di formazione universitaria, per favore, indica il tipo di facoltà frequentata. Es: Ingegneria civile; Lettere; Lingue; Ingegneria gestionale; Economia etc...)

- Elementari
- Medie
- Superiori
- Laurea Triennale
- Laurea Magistrale o a Ciclo Unico
- Master Universitario di I livello
- Master Universitario di II livello
- Diploma di Specializzazione
- Dottorato di Ricerca

Anni di scolarità

Sei madrelingua Italiano?

- Si
- No

avanti

Familiarity

InStance

99% completato

Hai esperienza con i robot? Se sì, che tipo di esperienza e con quale robot?

- No
- Si, lavoro con/ programmo robot umanoidi
- Si, sono molto interessato a libri e film di robots, umanoidi e Intelligenza Artificiale
- Si, ho partecipato a studi sull'Interazione Uomo-Robot
- Si, altro (Per favore descrivila tua esperienza con i robot)

avanti

Human Control Questionnaire
Supplementary material 3 English

Item 3 Control

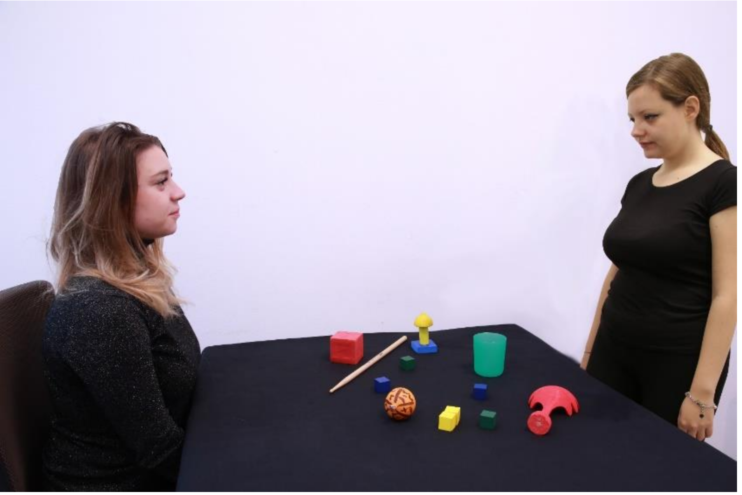

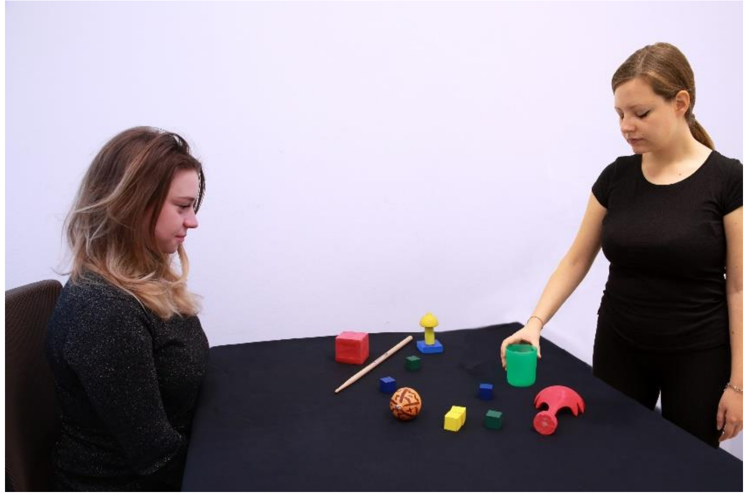

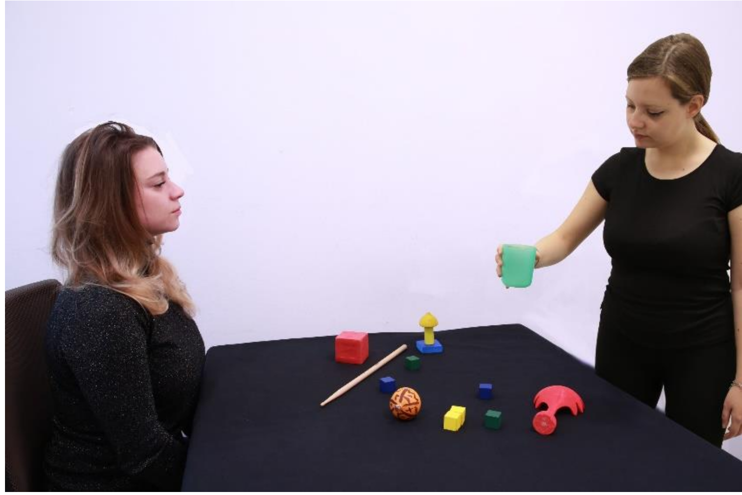

Paola grasps cylindrical objects best.

Paola believes that the girl likes the cup.

M= 52.05; SD: 41.61

Item 4 Control

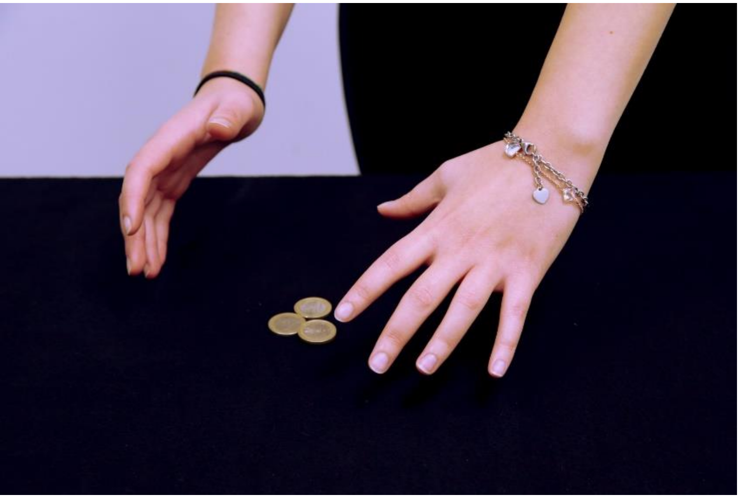

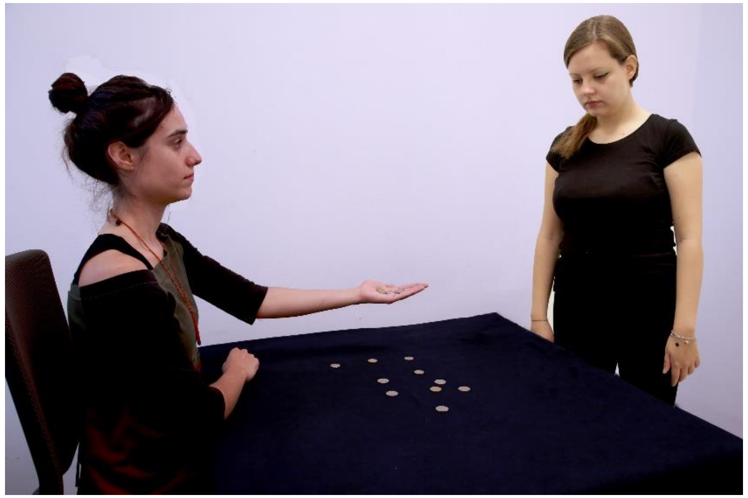

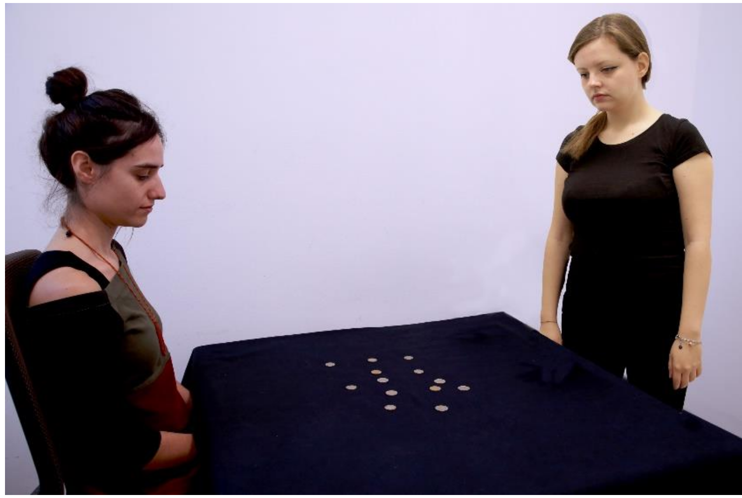

Paola is calculating the number coins.

Paola decided to give back the money.

M= 51.47; SD: 42.90

Item 8 Control

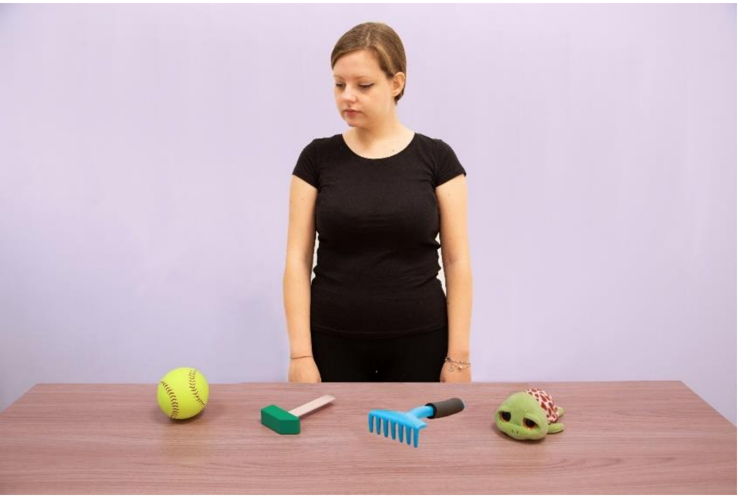

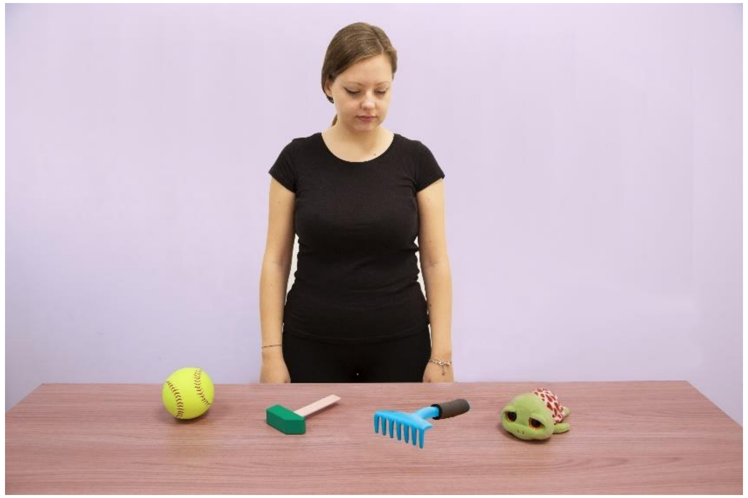

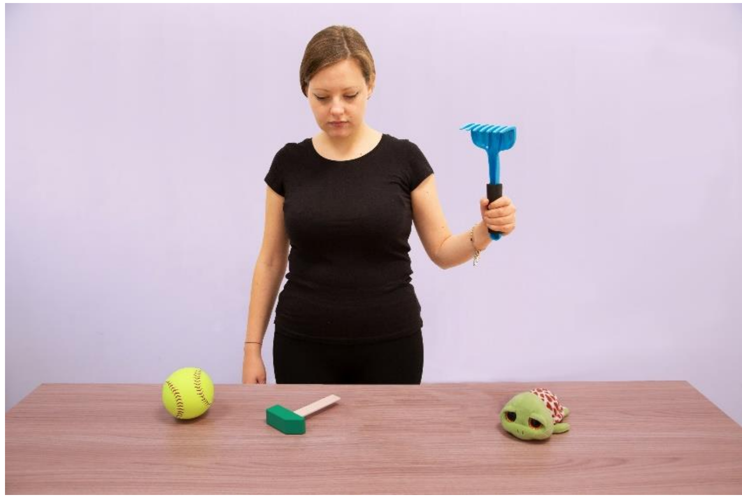

Paola adjusts the force to the weight of the object.

Paola pretends to be a gardener.

M= 42.25; SD: 40.90

Item 10 Control

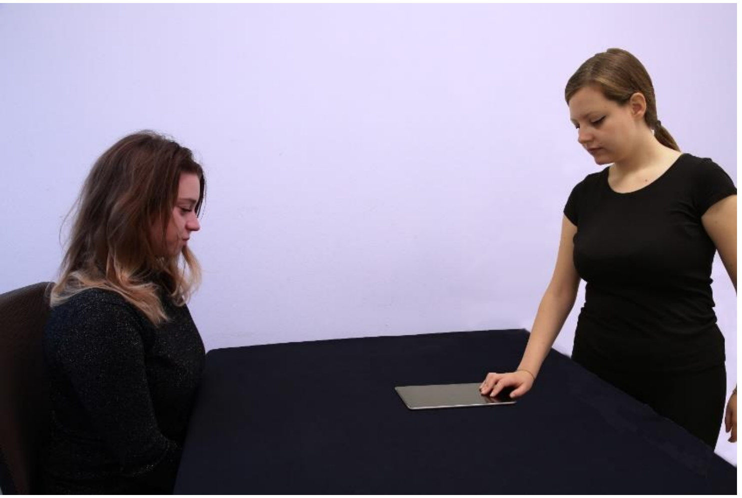

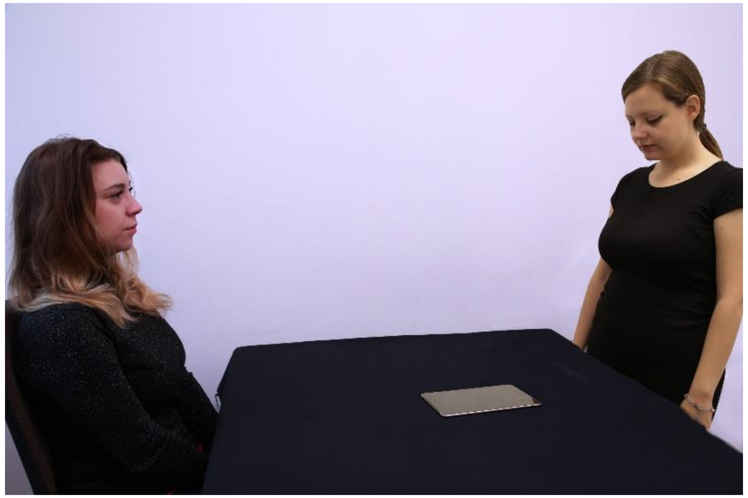

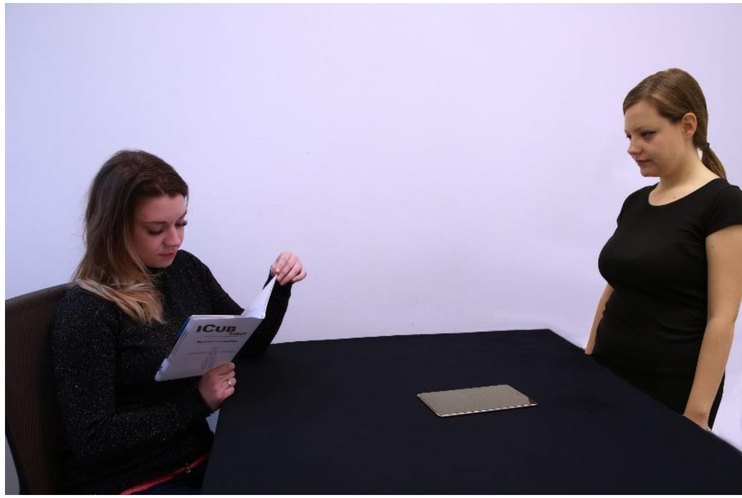

Paola detected differences between the old and the new object in the scene.

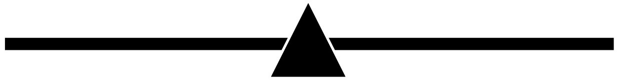

Paola wants to read from the book too.

M= 45.61; SD: 40.99

Item 11 Control

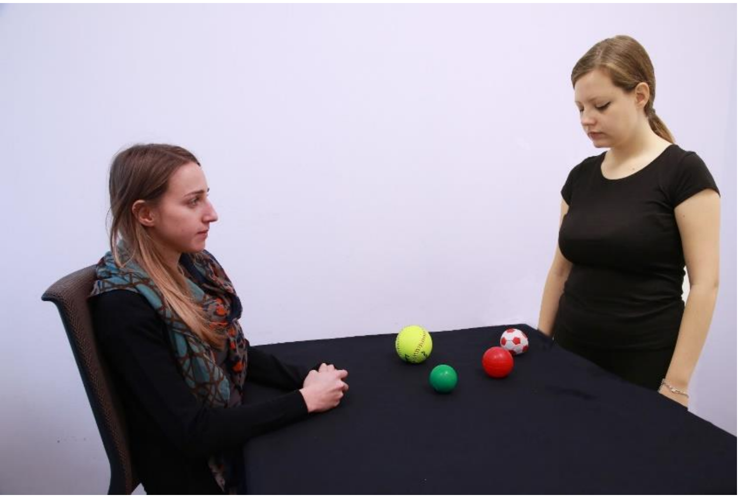

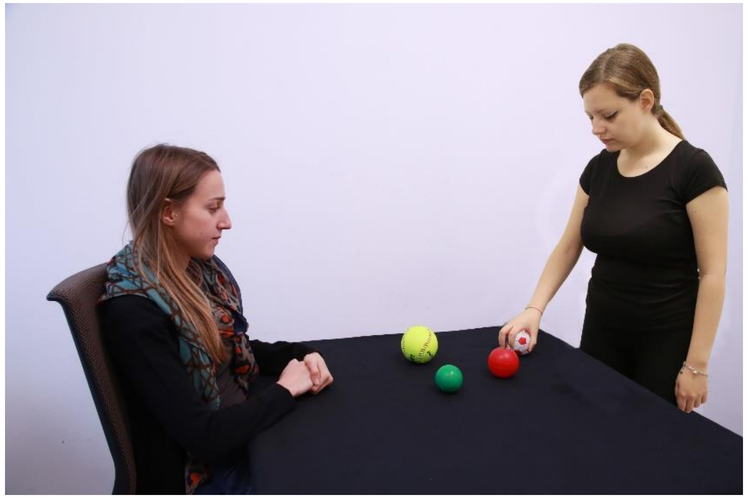

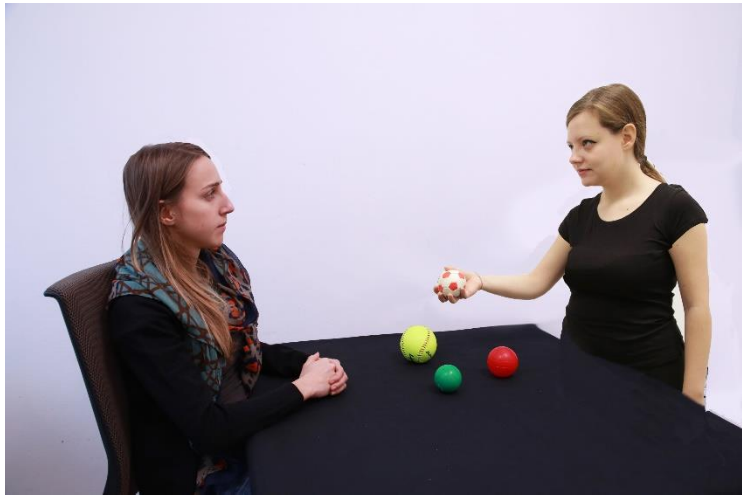

Paola calculates the weight of the balls.

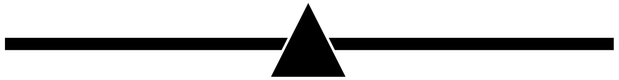

Paola wants to play with the girl.

M= 74.92; SD: 36.36

Item 13 Control

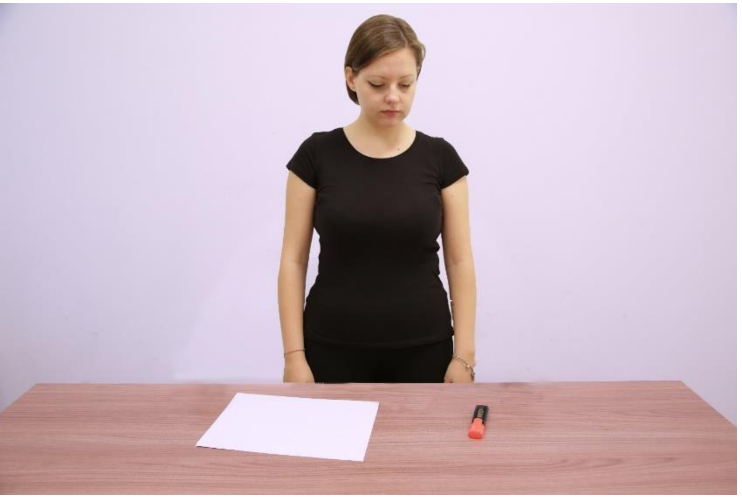
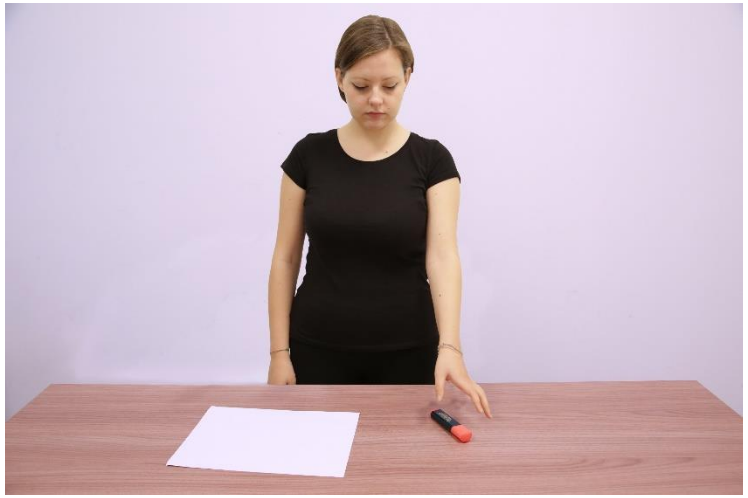
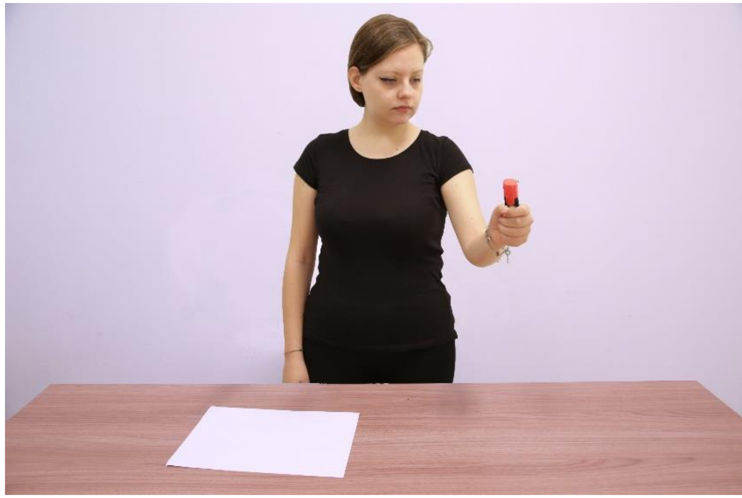

Paola optimizes grip for small objects.

Paola wants to draw something.

M= 56.47; SD: 42.87

Item 16 Control

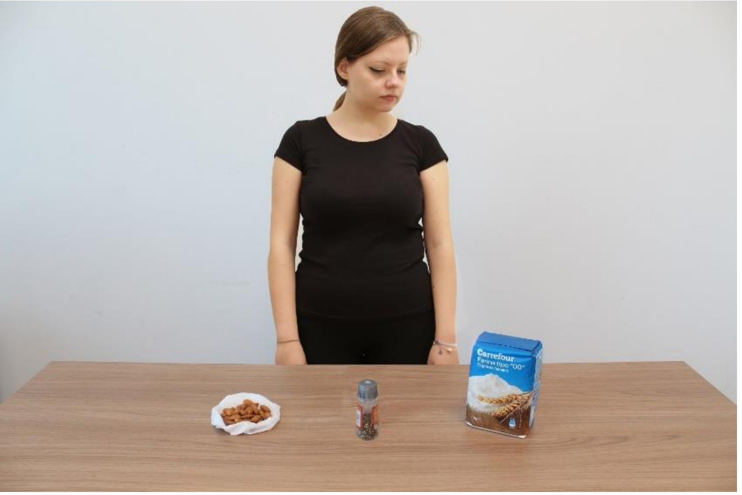

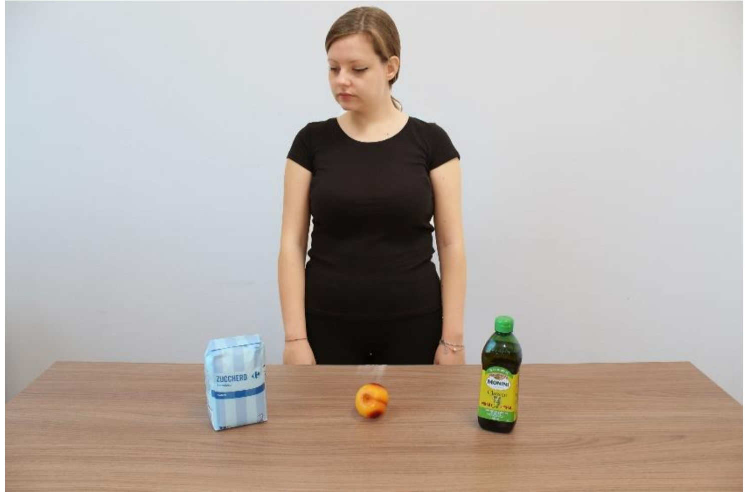

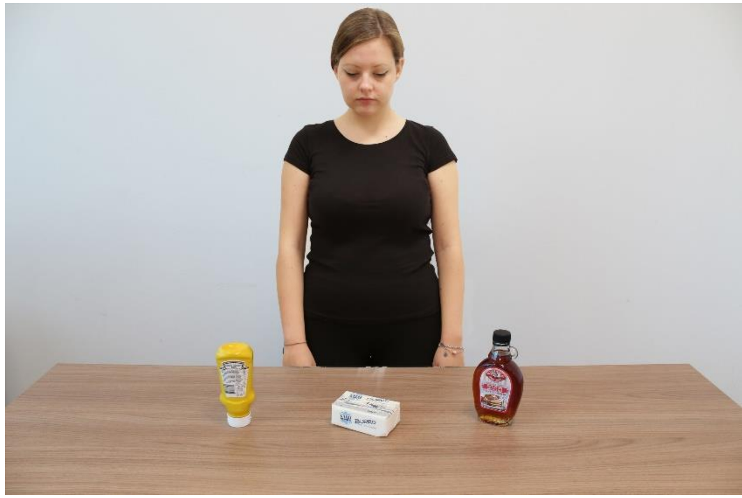

Paola follows the recipe's instructions.

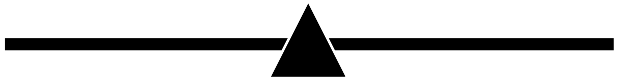

Paola has decided to bake a cake.

M= 51.97; SD: 41.55

Item 18 Control

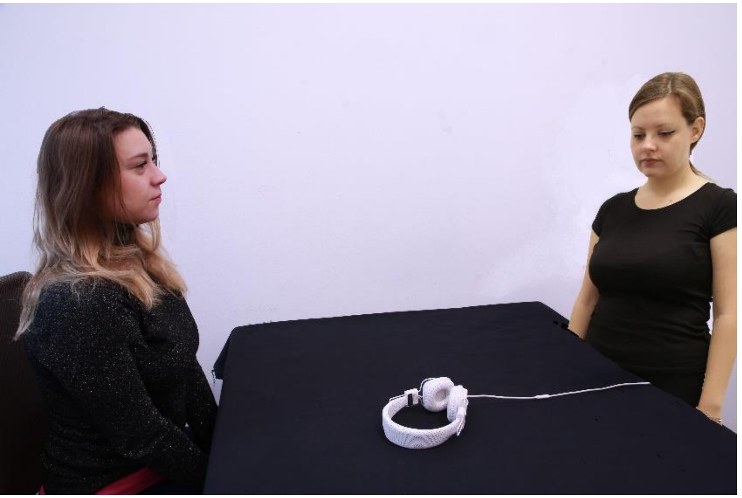

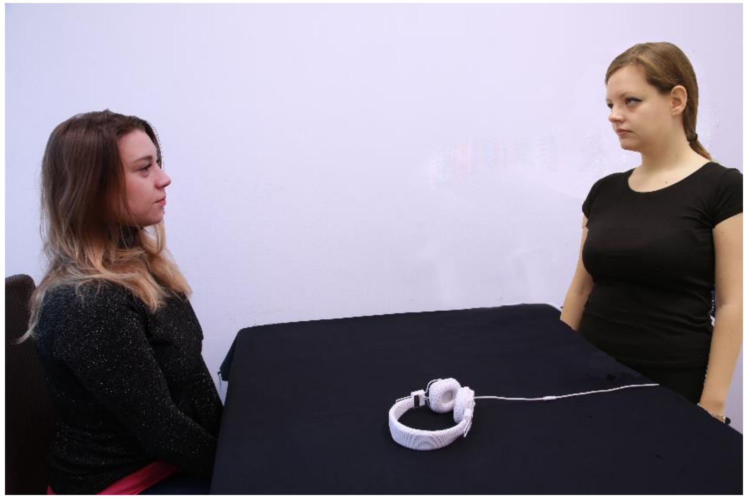

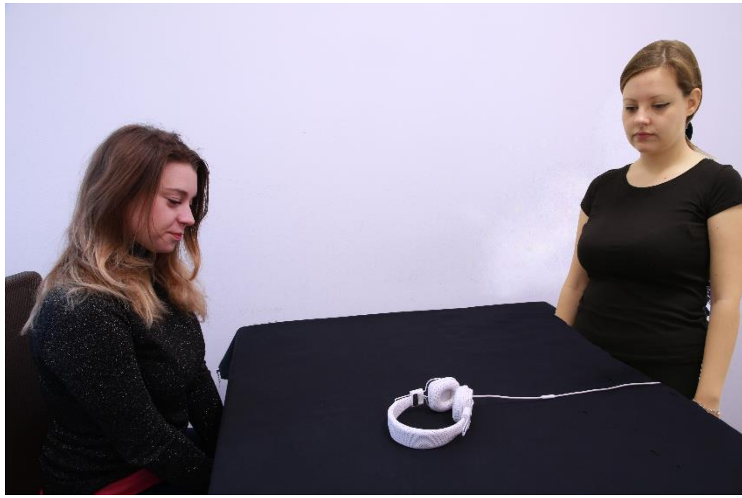

Paola measures the distance between the girl and the headphones.

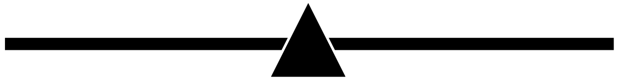

Paola expects that the girl would lend the headphones.

M= 66.82; SD: 37.46

Item 21 Control

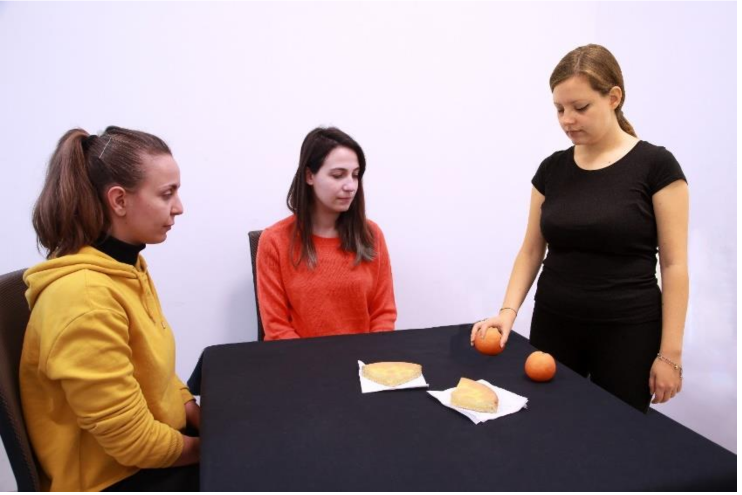

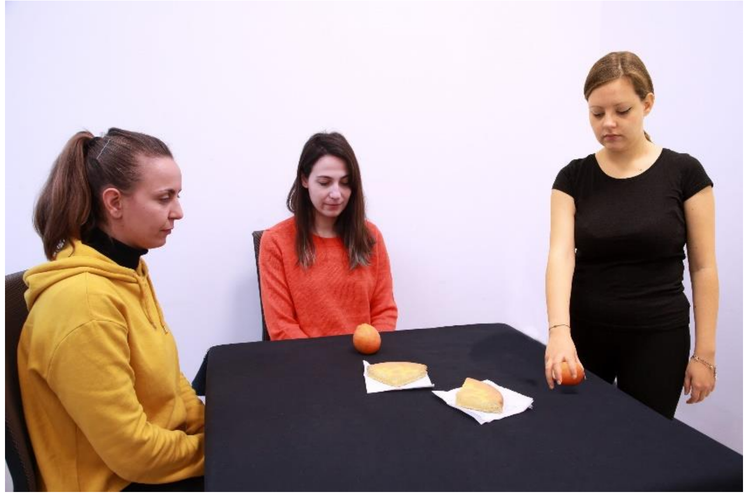

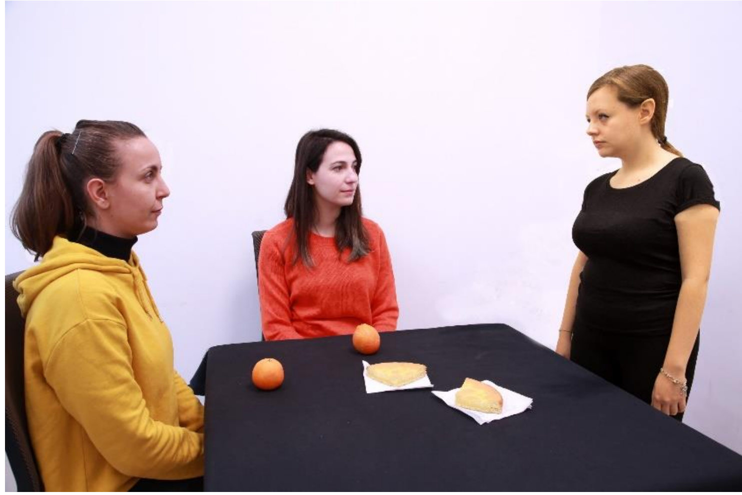

Paola can grasp oranges easier than cakes.

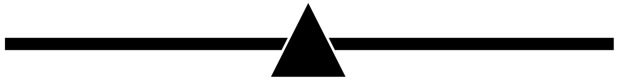

Paola thinks that the girls need some healthy food.

M= 35.99; SD: 42.48

Item 22 Control

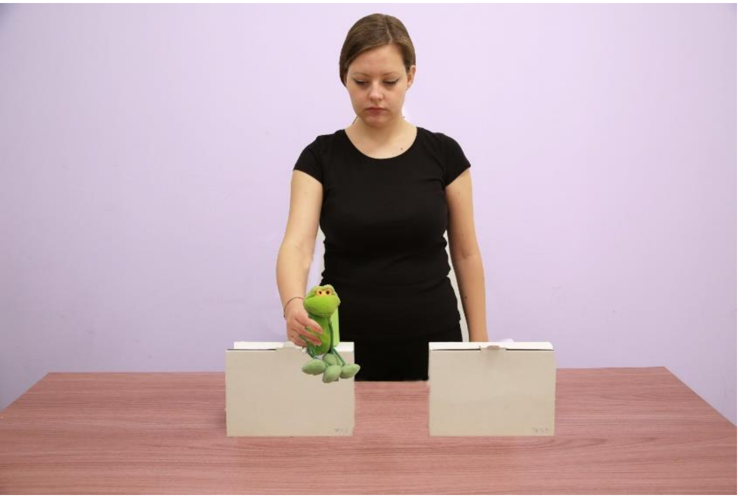
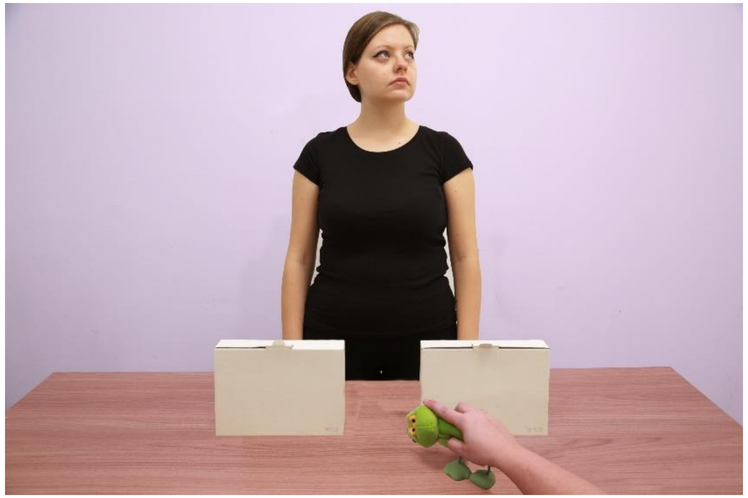
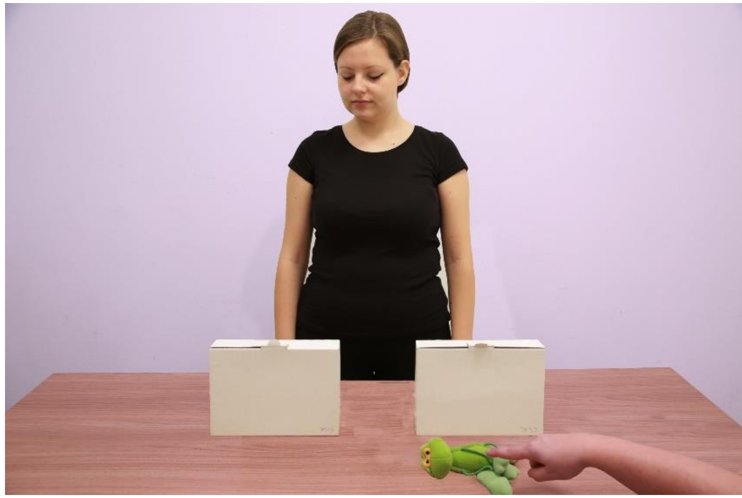

Paola is turning the head to the initial position.

Paola is not interested in the toy anymore.

M= 59.30; SD: 41.65

Item 23 Control

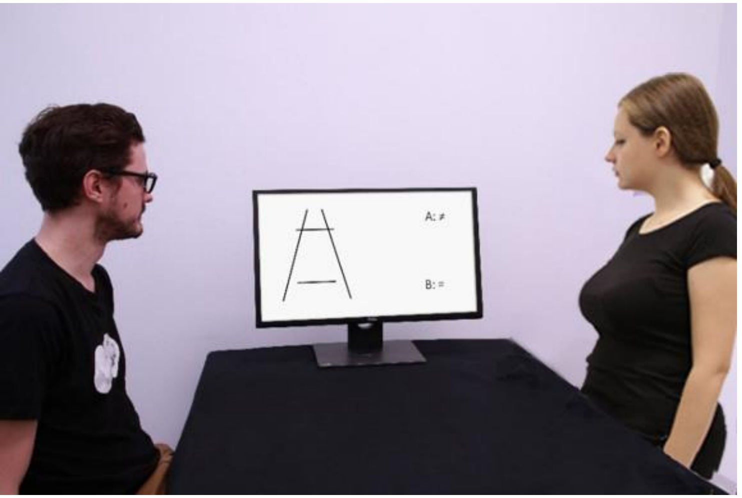

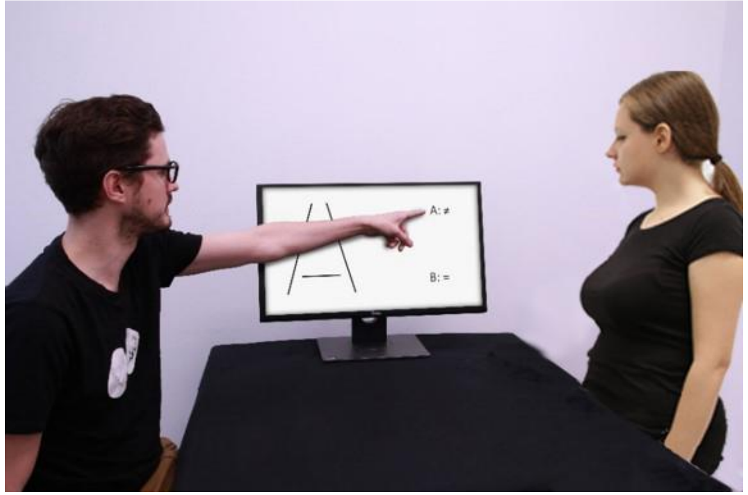

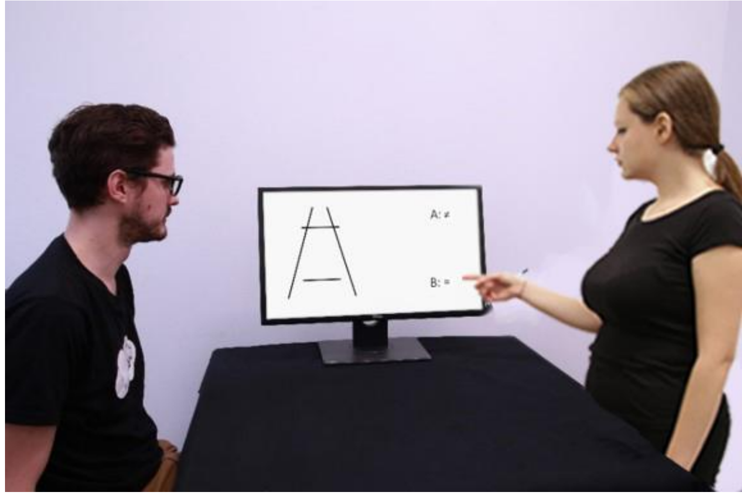

Paola is repeating the pointing movement.

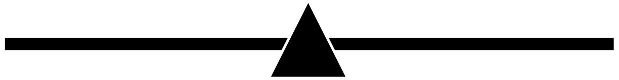

Paola is expressing an opinion.

M= 78.22; SD: 33.44

Item 25 Control

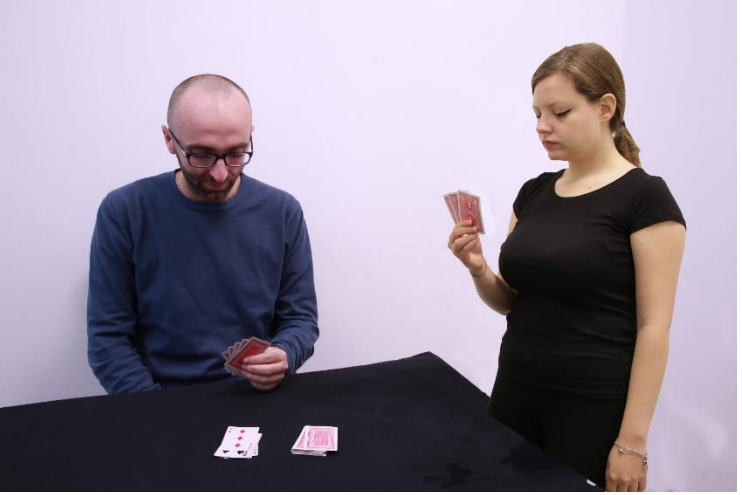

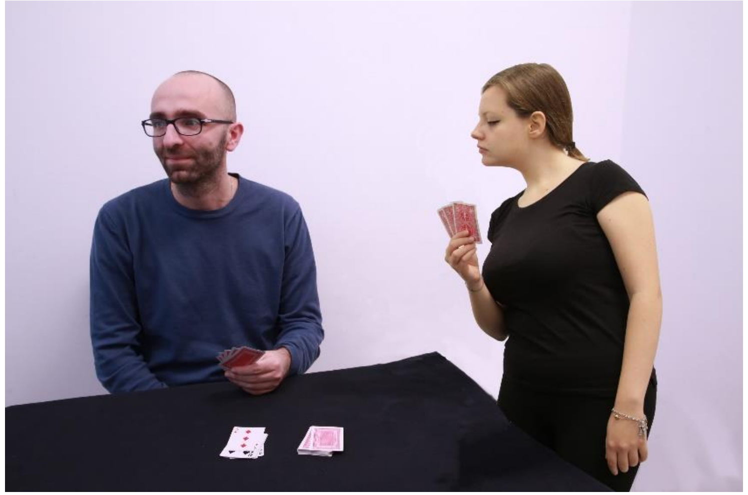

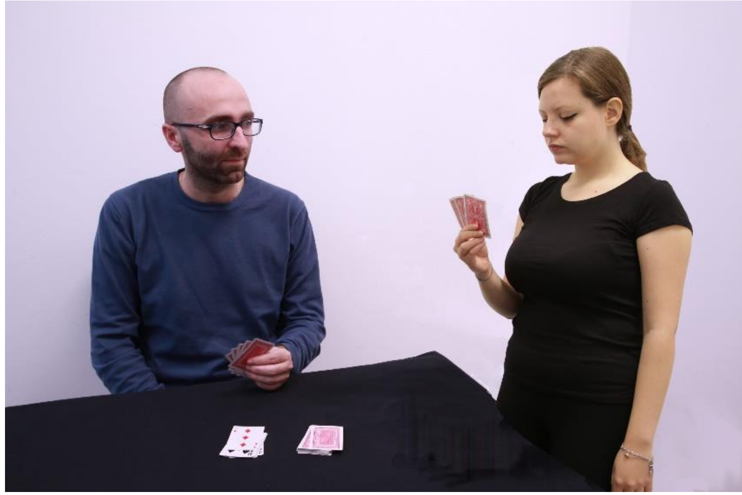

Paola was unbalanced for a moment.

Paola was trying to cheat by looking at opponent’s cards.

M= 72.58; SD: 37.44

Item 28 Control

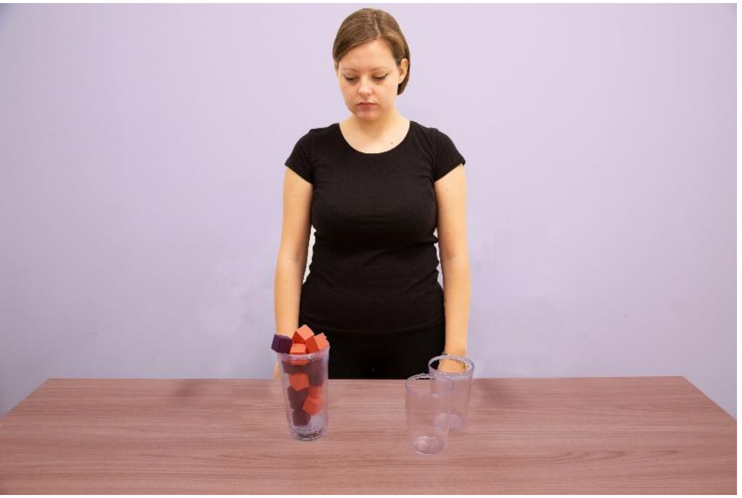

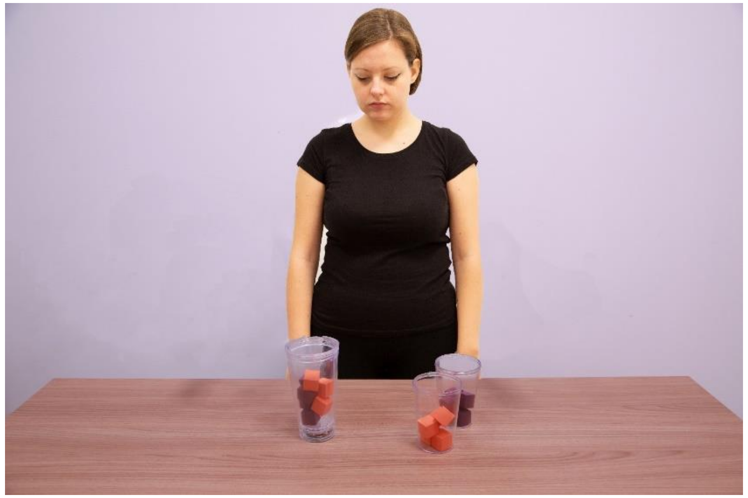

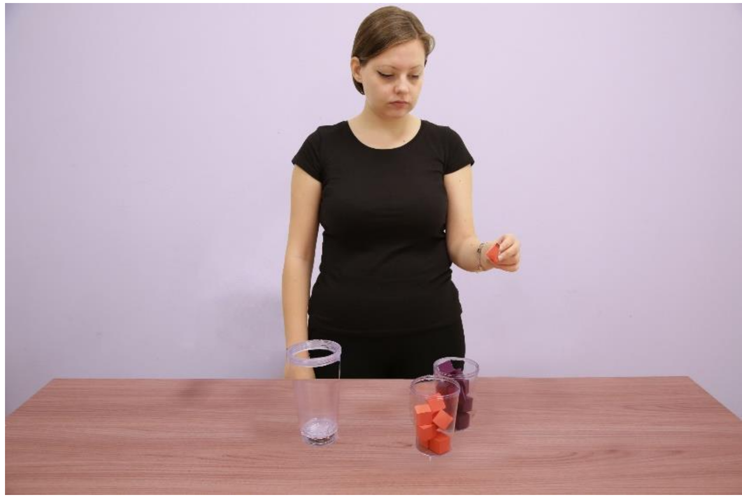

Paola classifies cubes by color.

Paola would like to keep this cube.

M= 43.53; SD: 45.63

Item 31 Control

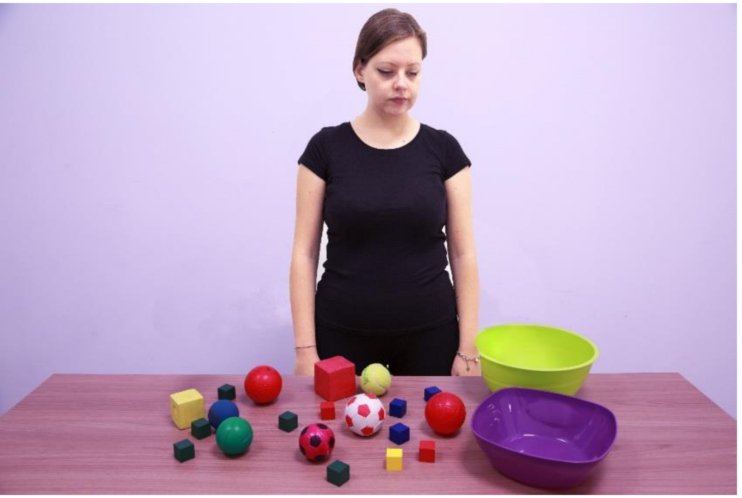

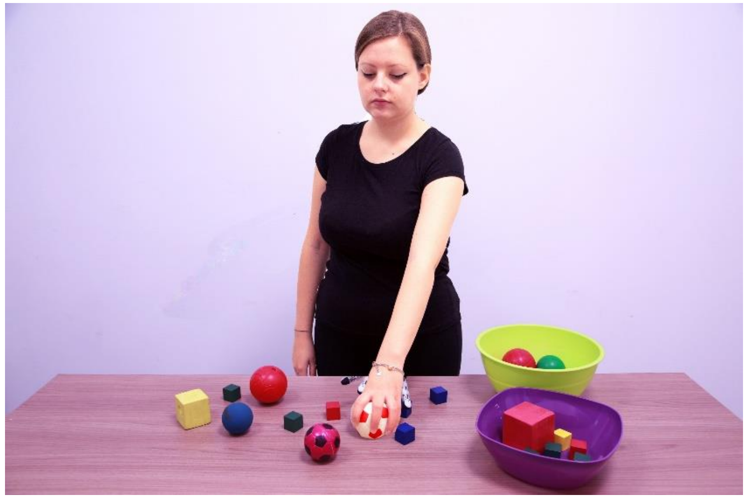

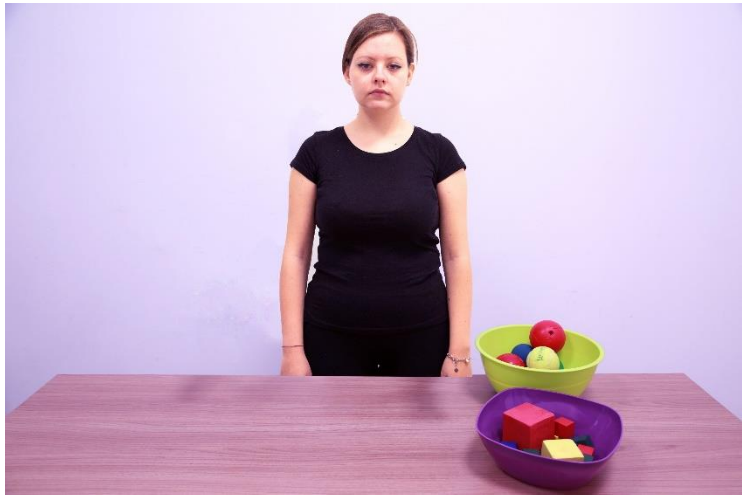

Paola places objects in bowls with corresponding shape.

Paola knows that it's better to clean the table after playing.

M= 53.76; SD: 44.95

Item 33 Control

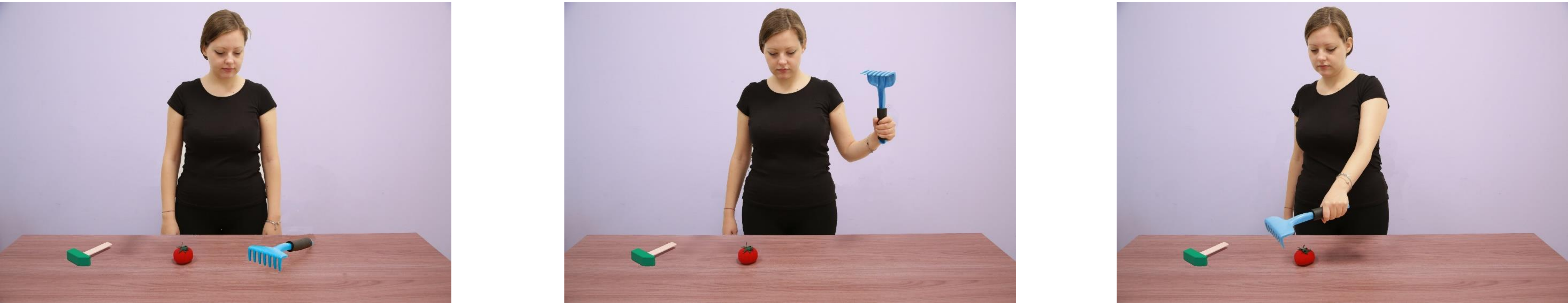

Paola grasped the closest object.

Paola was fascinated by tool use.

M= 34.33; SD: 40.75

Human Control Questionnaire Supplementary material 3 ITALIAN

Item 3 Control

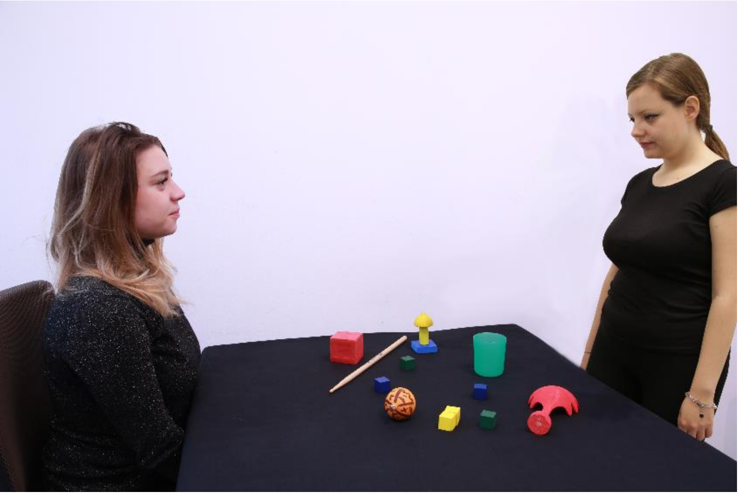

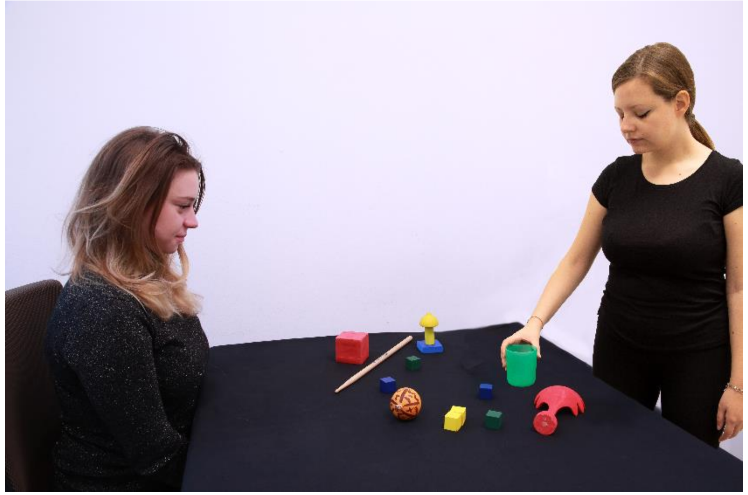

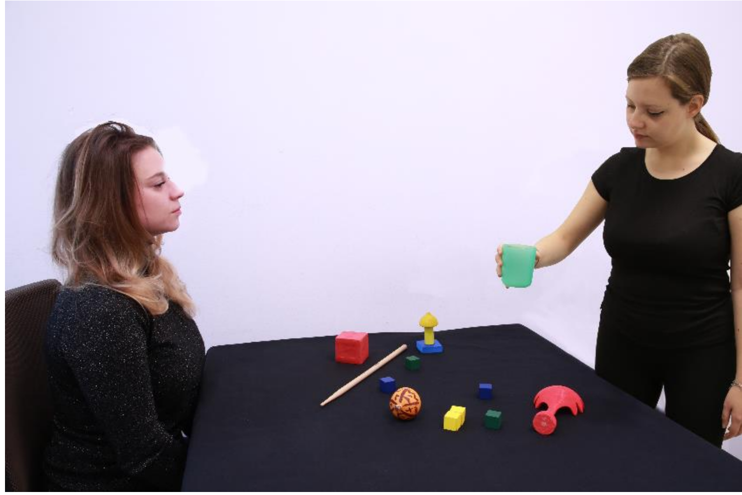

Paola afferra meglio gli oggetti cilindrici.

Paola pensa che alla ragazza piaccia il bicchiere.

M= 52.05; SD: 41.61

Item 4 Control

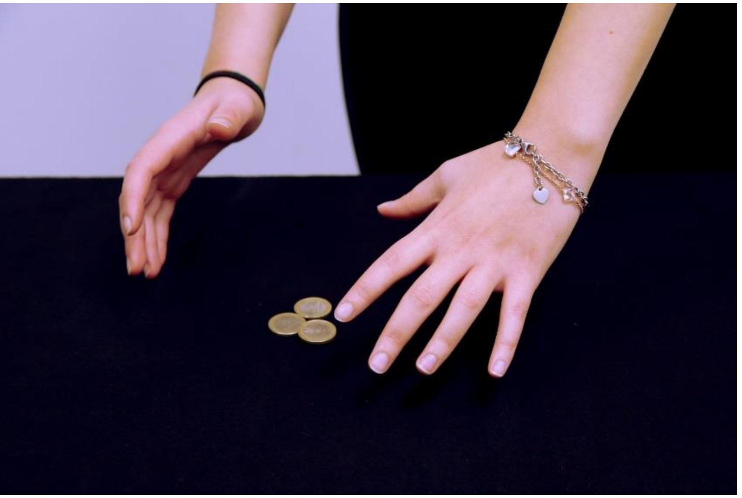

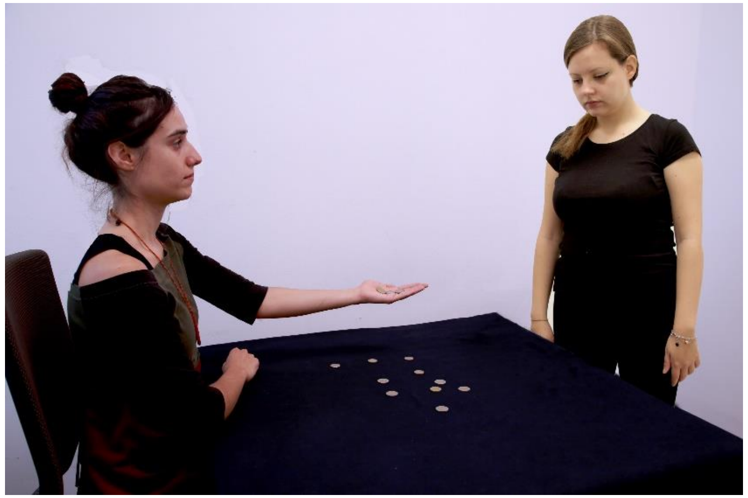

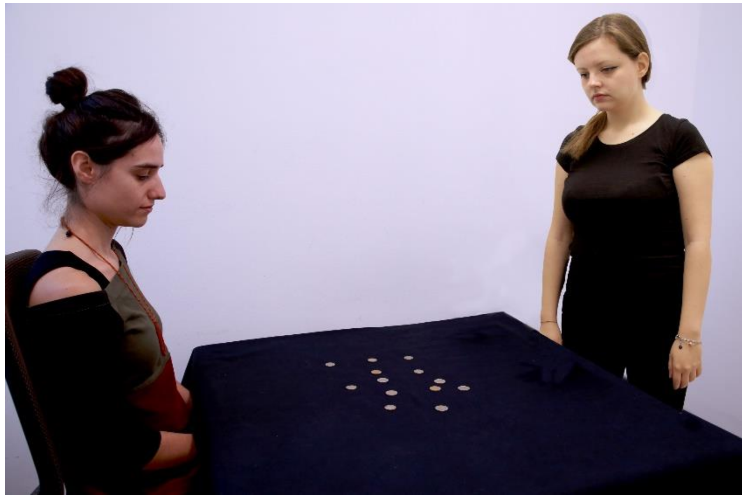

Paola calcola il numero di monete.

Paola ha deciso di restituire le monete.

M= 51.47; SD: 42.90

Item 8 Control

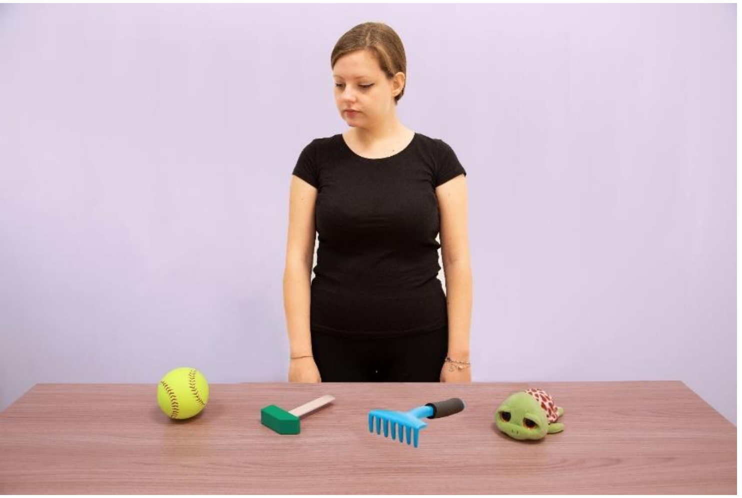

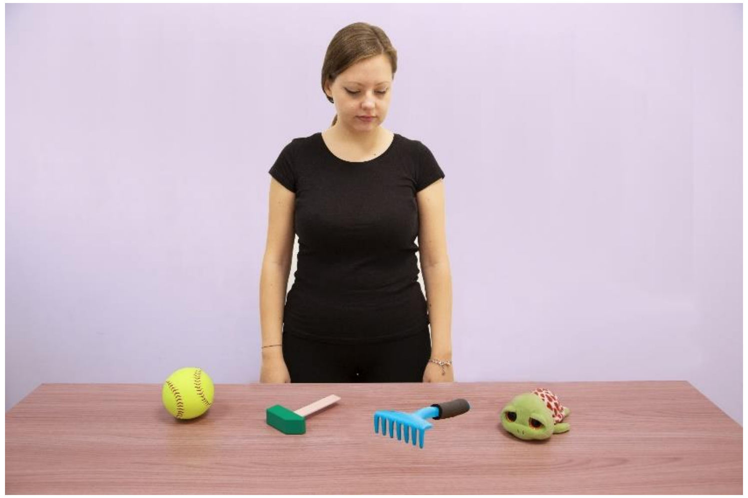

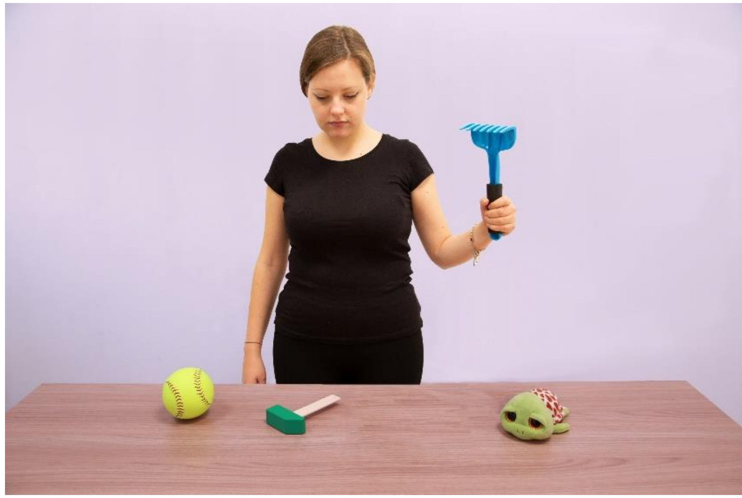

Paola regola la forza in base al peso dell'oggetto.

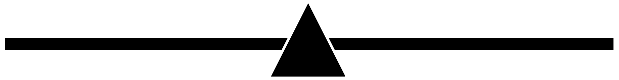

Paola finge di essere un giardiniere.

M= 42.25; SD: 40.90

Item 10 Control

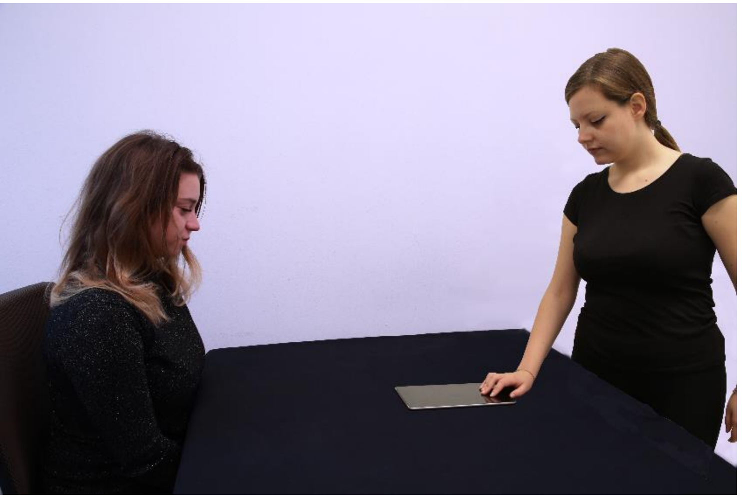

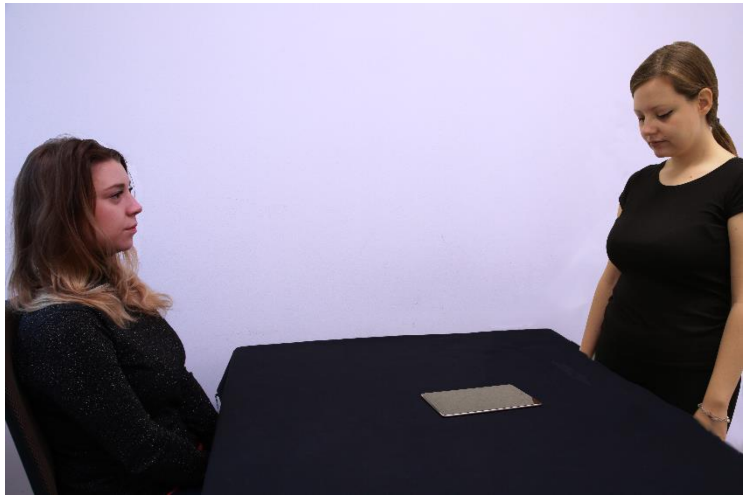

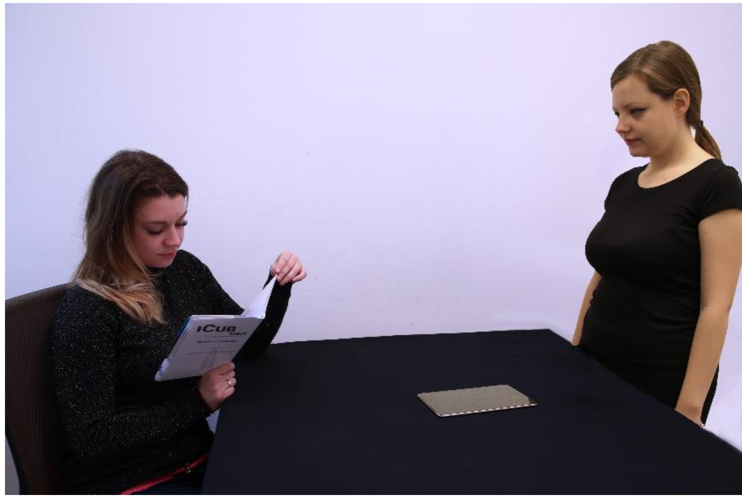

Paola rileva differenze tra il vecchio e il nuovo oggetto in scena.

Paola vuole leggere il libro.

M= 45.61; SD: 40.99

Item 11 Control

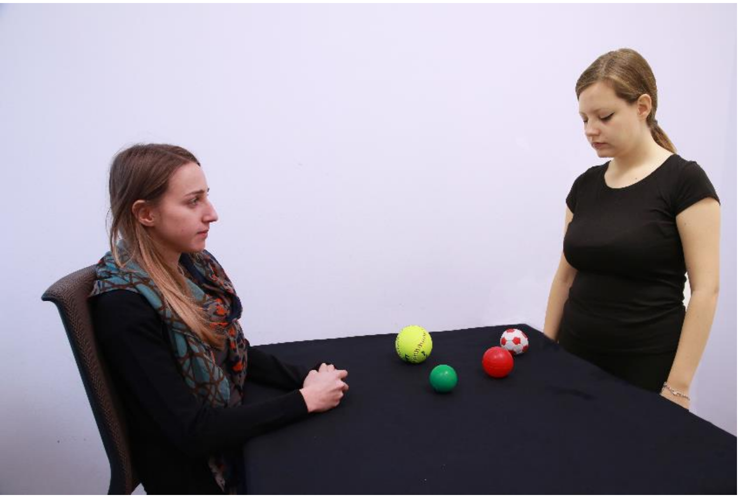

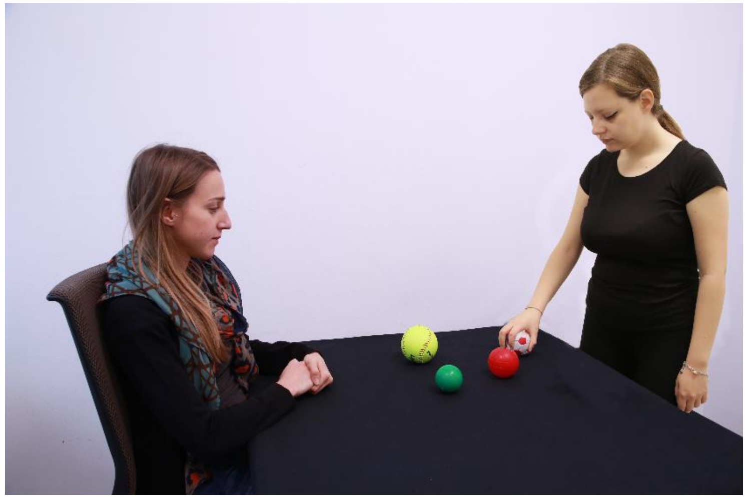

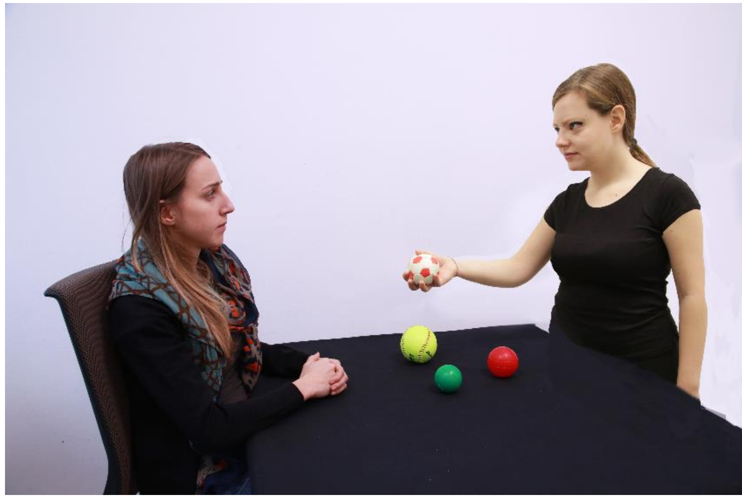

Paola calcola il peso delle palle.

Paola vuole giocare con la ragazza.

M= 74.92; SD: 36.36

Item 13 Control

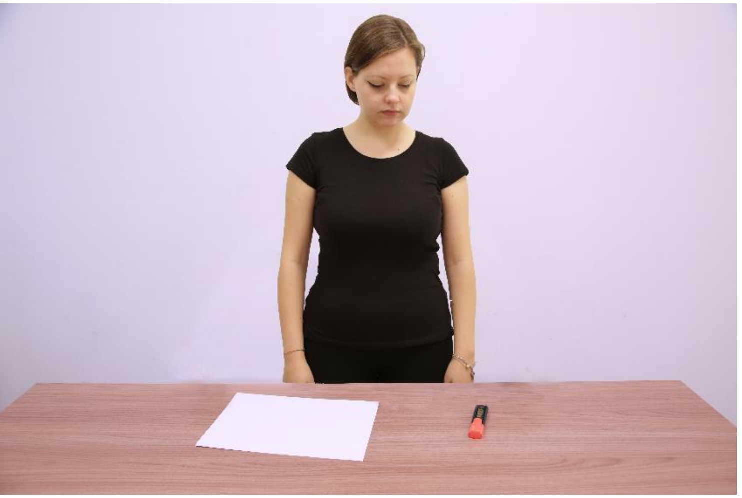

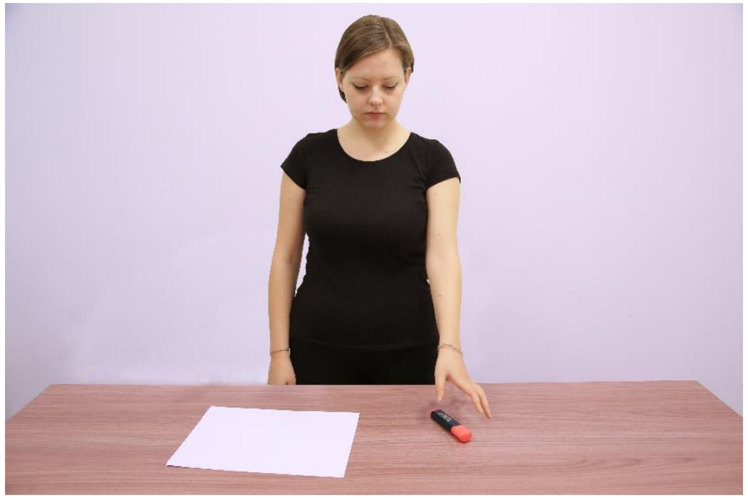

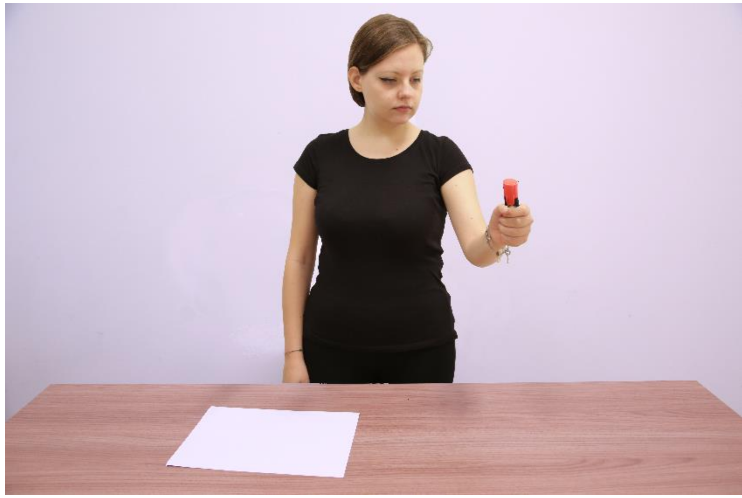

Paola ottimizza la presa gli oggetti piccoli.

Paola vuole disegnare qualcosa.

M= 56.47; SD: 42.87

Item 16 Control

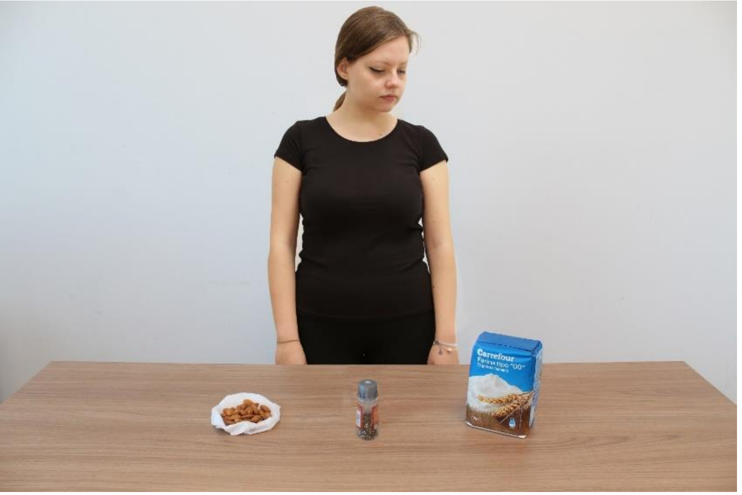

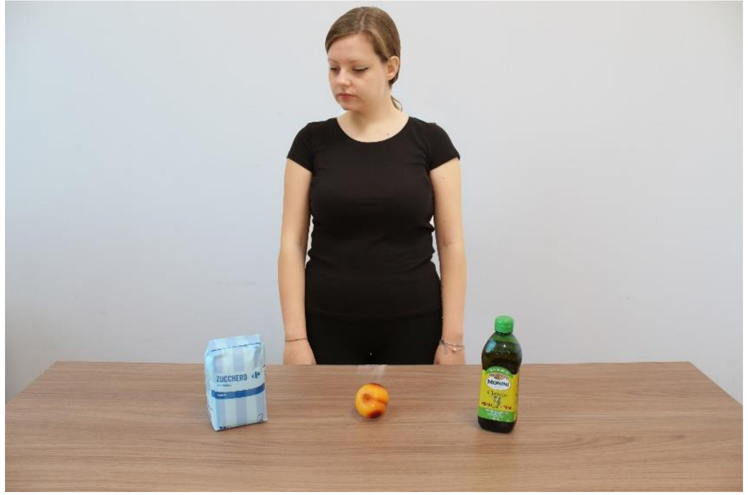

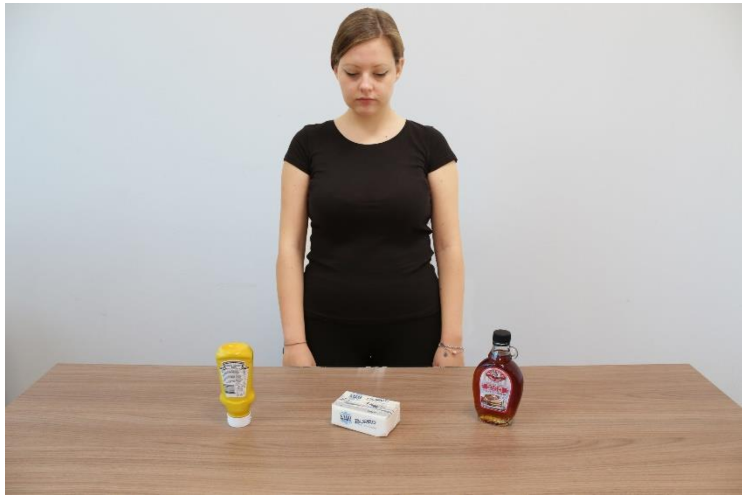

Paola segue le istruzioni della ricetta.

Paola ha deciso di cucinare una torta.

M= 51.97; SD: 41.55

Item 18 Control

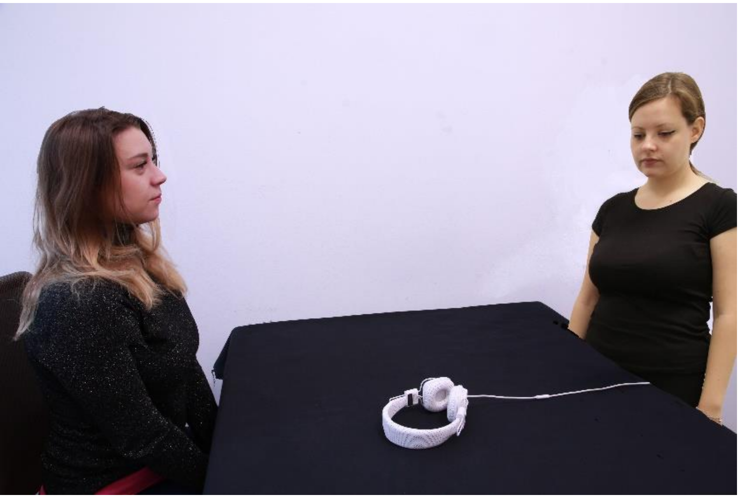

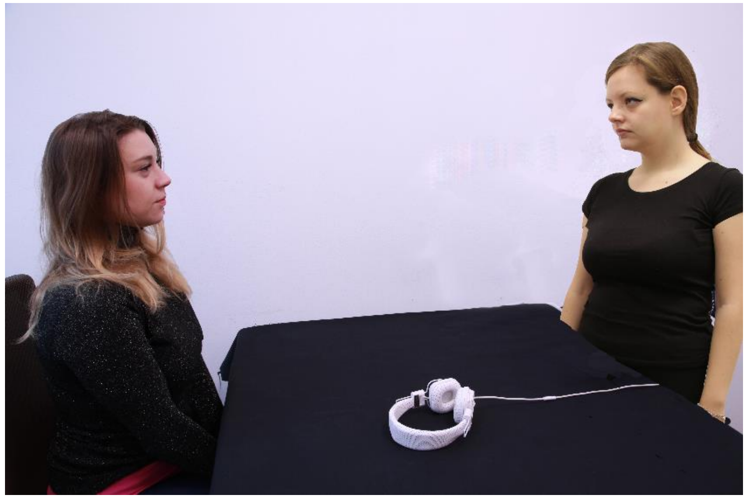

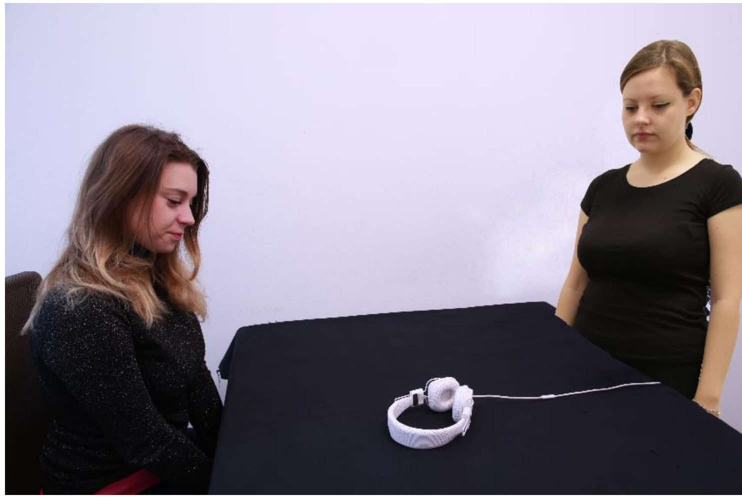

Paola misura la distanza tra la ragazza e le cuffie.

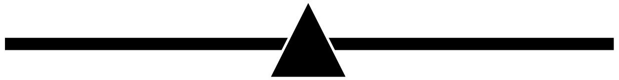

Paola si aspetta che la ragazza dia in prestito le cuffie.

M= 66.82; SD: 37.46

Item 21 Control

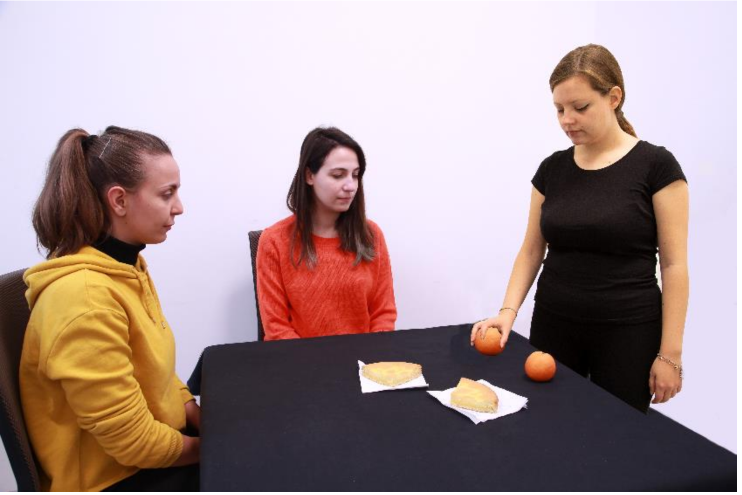

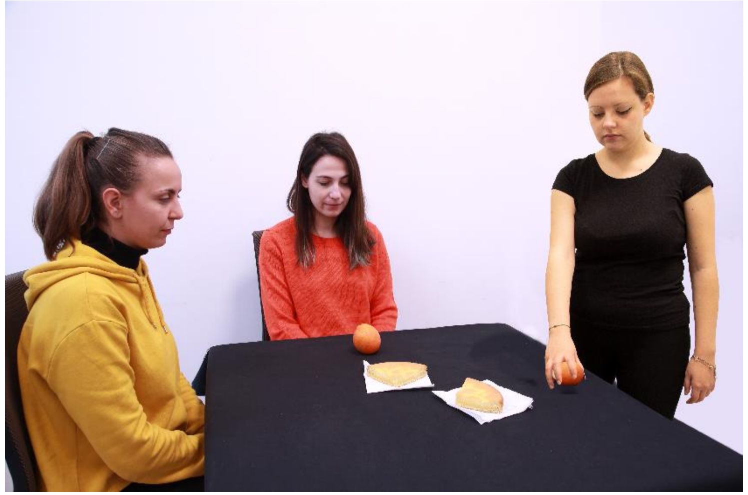

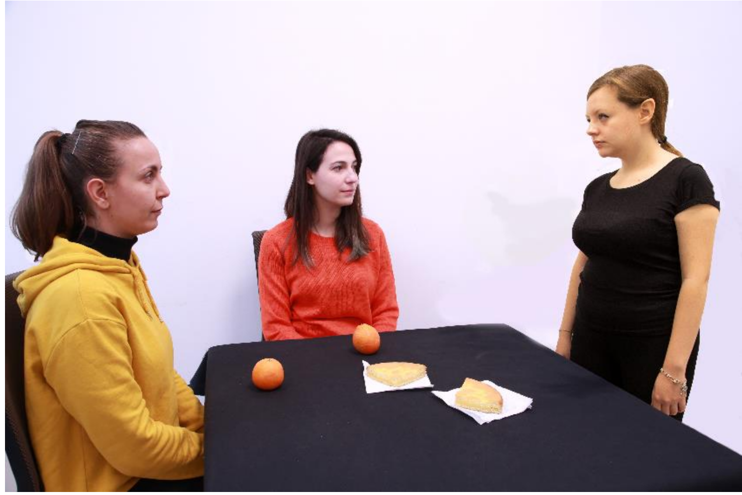

Paola pensa che le ragazze abbiano bisogno di cibo sano.

Paola può afferrare le arance più facilmente della torta.

M= 35.99; SD: 42.48

Item 22 Control

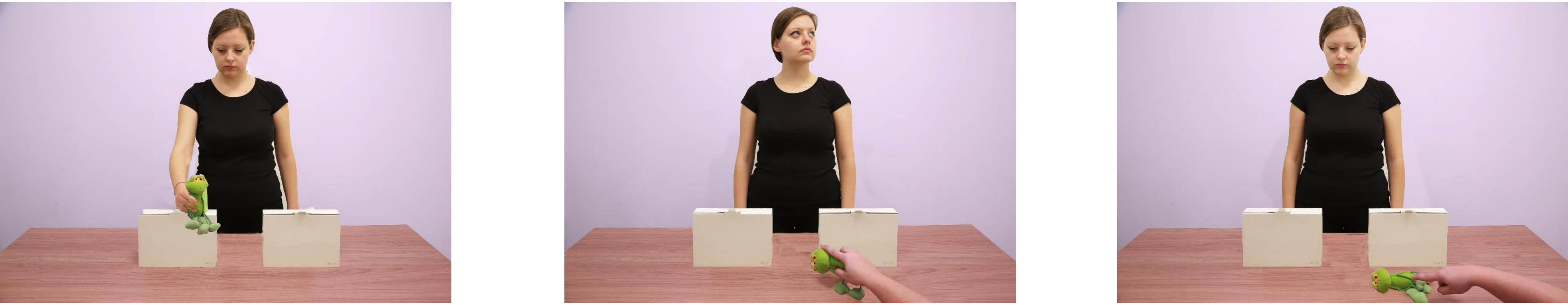

Paola riporta la testa nella posizione iniziale.

Paola non è più interessata al giocattolo.

M= 59.30; SD: 41.65

Item 23 Control

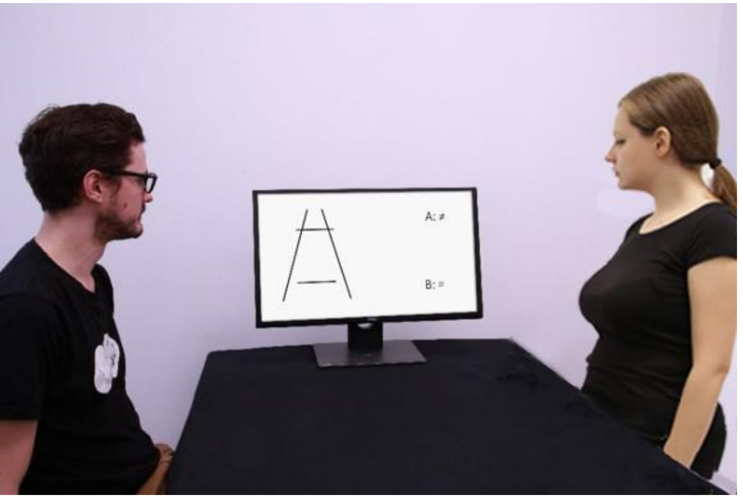

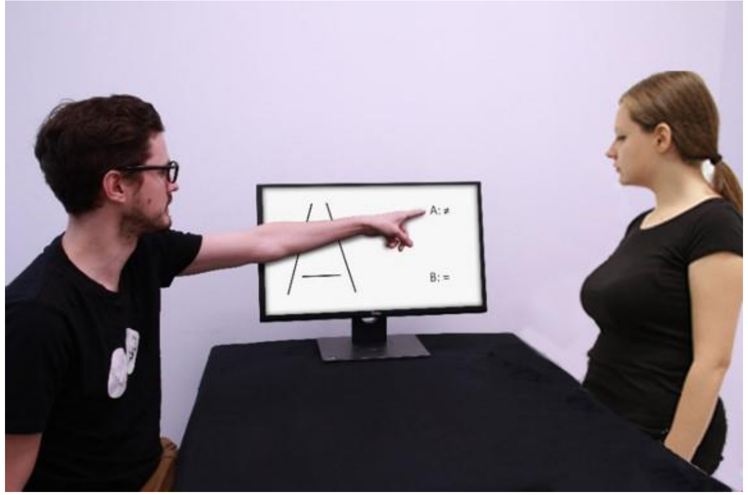

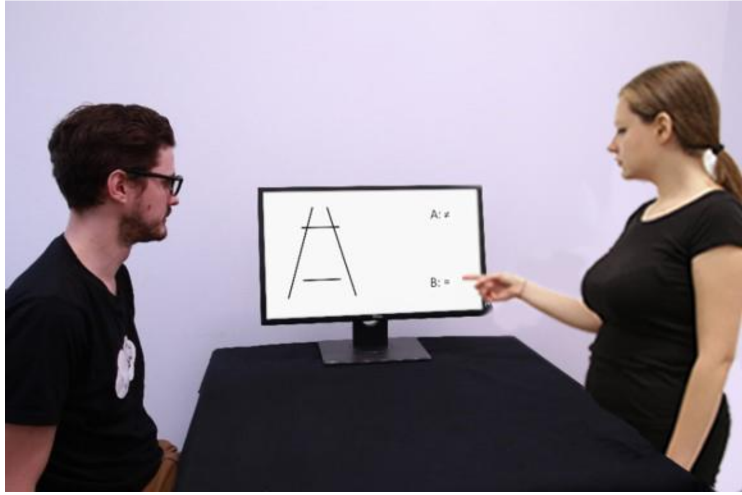

Paola sta ripetendo il gesto di indicare.		Paola sta esprimendo un’opinione.

M= 78.22; SD: 33.44

Item 25 Control

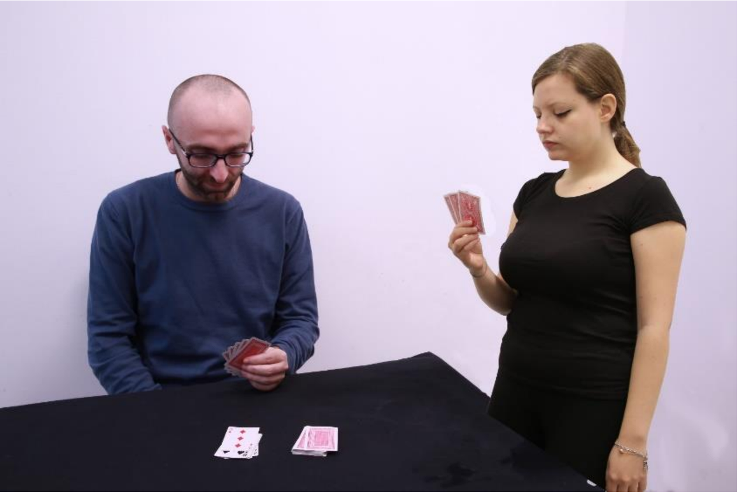
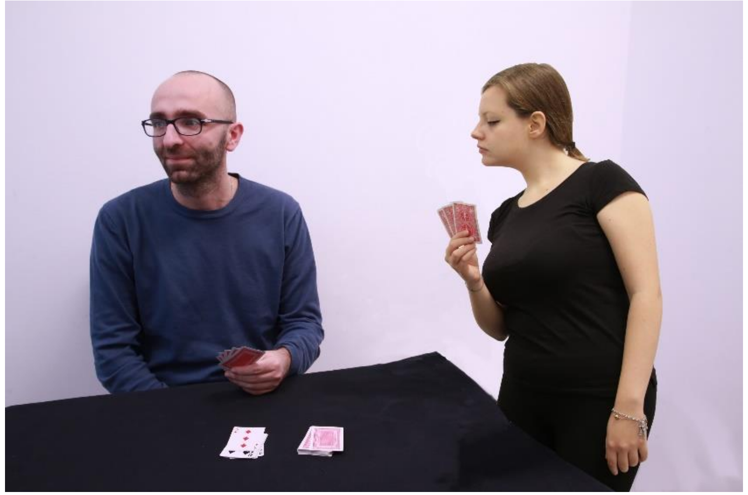
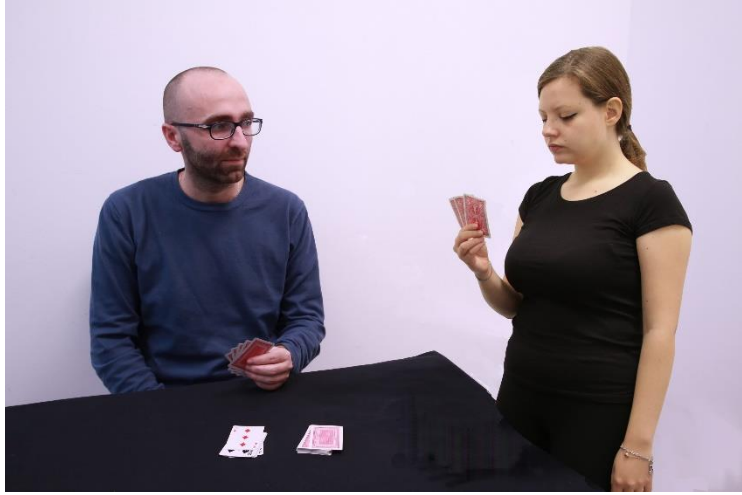

Paola bara guardando le carte dell’avversario.

Paola ha perso l'equilibrio per un momento.

M= 72.58; SD: 37.44

Item 28 Control

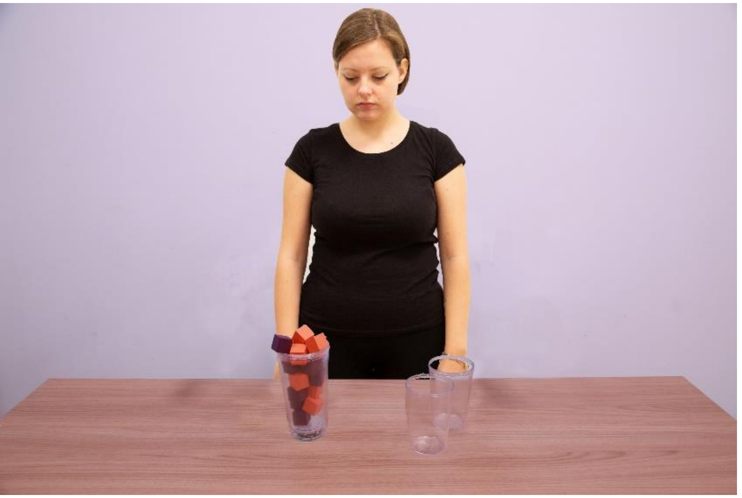
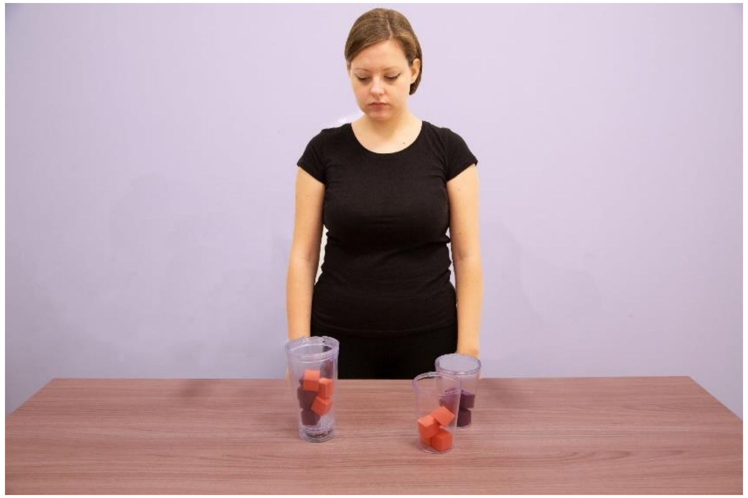
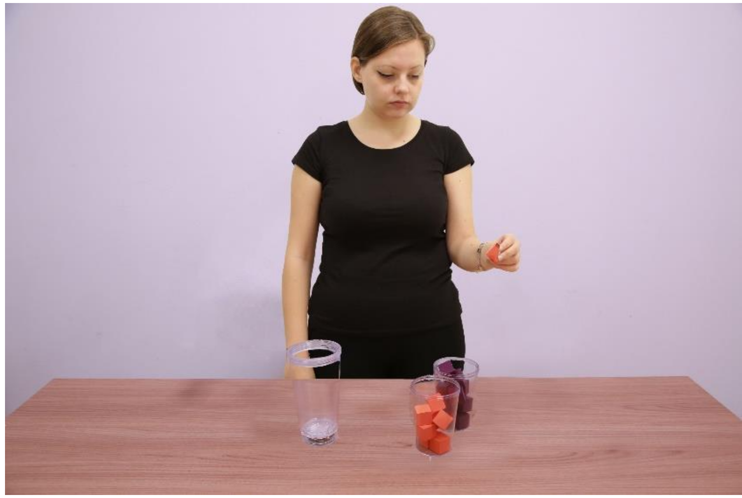

Paola classifica i cubi in base al colore.

Paola vorrebbe tenere questo cubo.

M= 43.53; SD: 45.63

Item 31 Control

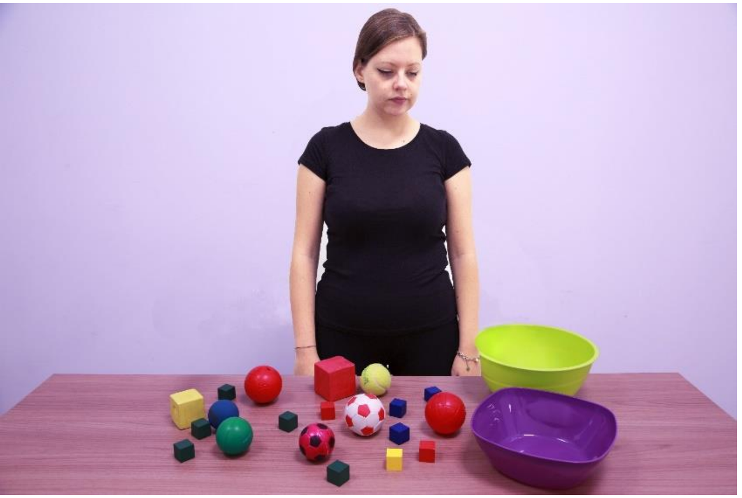

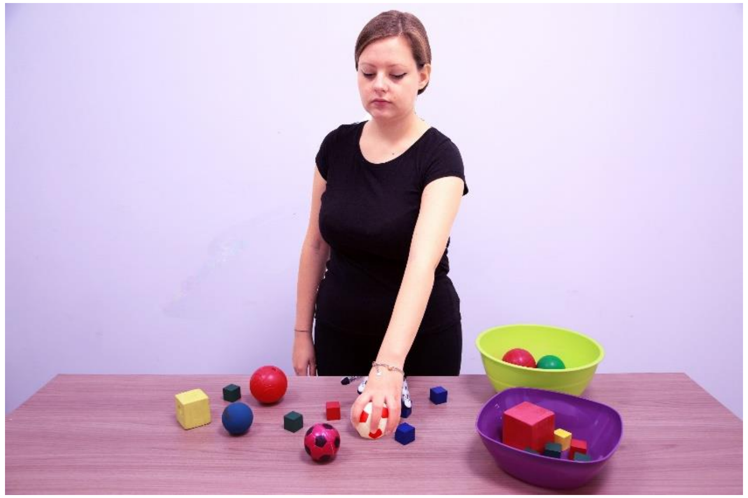

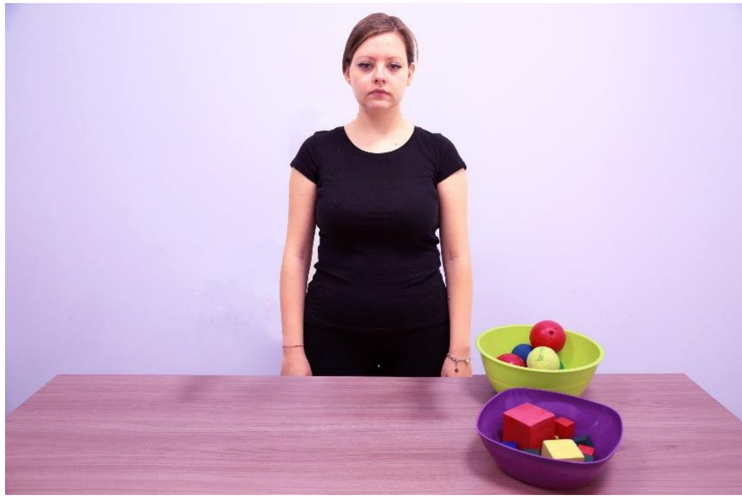

Paola mette gli oggetti nel contenitore con la forma corrispondente.

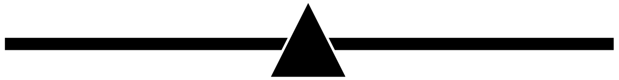

Paola sa che è giusto mettere in ordine il tavolo dopo aver giocato.

M= 53.76; SD: 44.95

Item 33 Control

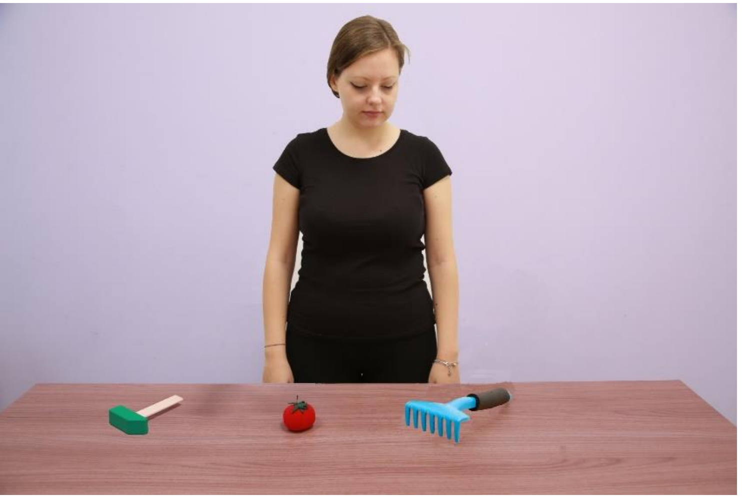 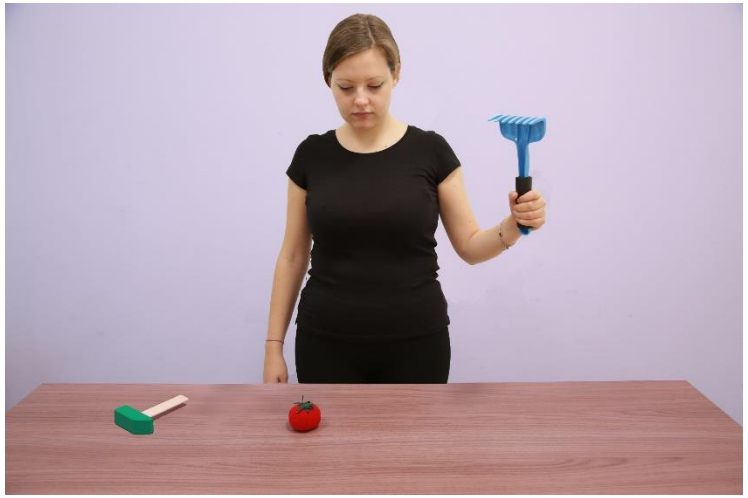 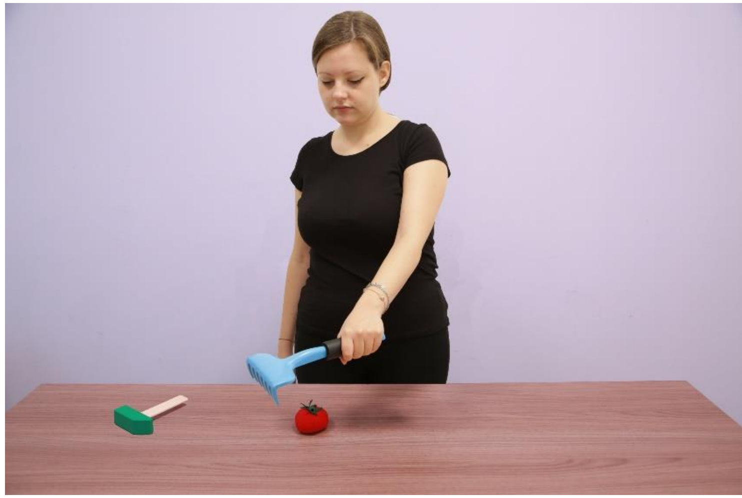

Paola afferra
l’oggetto più vicino.

Paola è affascinata
dall’uso degli utensili.

M= 34.33; SD: 40.75

Demographics

17% completato

Prima di procedere con l'inizio del questionario ti preghiamo di rispondere ad alcune domande utili allo svolgimento dello studio.

Il questionario è totalmente anonimo. Il Codice Identificativo è necessario per mantenere tale anonimato. Puoi inserire una stringa di lettere e numeri a tua scelta.

Codice Identificativo	
Sesso (M/F)	
Età	
Numero Figli	
Numero Fratelli/Sorelle	
Occupazione	

Scolarità (In caso di formazione universitaria, per favore, indica il tipo di facoltà frequentata. Es: Ingegneria civile; Lettere; Lingue; Ingegneria gestionale; Economia etc...)

- Elementari
- Medie
- Superiori
- Laurea Triennale
- Laurea Magistrale o a Ciclo Unico
- Master Universitario di I livello
- Master Universitario di II livello
- Diploma di Specializzazione
- Dottorato di Ricerca

Anni di scolarità

Sei madrelingua Italiano?

- Si
- No

avanti

Familiarity

89% completato

Hai esperienza con i robot? Se sì, che tipo di esperienza e con quale robot?

avanti
